# Development of a multidimensional disease burden assessment system for childhood neuroblastoma based on the social determinants of health theory

**DOI:** 10.3389/fonc.2026.1886324

**Published:** 2026-09-15

**Authors:** Xue Ban, Qingqing Chong, Wenshen Shao, Wenjing Liu, Mingqi Peng

**Affiliations:** Department of Nursing, Children’s Hospital of Nanjing Medical University, Nanjing, Jiangsu, China

**Keywords:** Delphi technique, disease burden, neuroblastoma, pediatric oncology, rare diseases, social determinants of health

## Abstract

**Objective:**

To construct a multidimensional disease burden assessment system for children with neuroblastoma based on the Social Determinants of Health (SDH) theory, and to provide a reference for targeted assistance for rare diseases and for the optimization of health resource allocation.

**Methods:**

An indicator pool was initially developed based on the SDH framework through a structured literature review and a structured expert panel discussion. From November to December 2025, 20 of the 21 invited experts participated in two rounds of Delphi consultation to finalize the indicators, and the Analytic Hierarchy Process was employed to calculate indicator weights.

**Results:**

The effective response rates of the 2 rounds of questionnaire surveys were 95.23% (20/21) and 100% (20/20), respectively. The expert authority coefficient was 0.885, and Kendall’s W coefficients were 0.123-0.452 and 0.118-0.236 for the 2 rounds (all P < 0.05), indicating high expert coordination. The constructed indicator system comprised 3 first-level indicators [personal disease burden (0.3325), family disease burden (0.5278), and social disease burden (0.1396)], 8 second-level indicators, and 32 third-level indicators.

**Conclusion:**

The indicator system constructed in this study is a preliminary, theory-driven framework supported by expert consensus and internally consistent weighting. It can provide a reference for health administrative departments to identify high-burden families and formulate assistance policies, thereby supporting health governance across the full care cycle for children with rare diseases.

## 1 Introduction

Neuroblastoma (NB) is a pediatric extracranial malignant solid tumor and was included in the Second List of Rare Diseases issued by the National Health Commission of China in 2023 (1). NB accounts for approximately 8-10% of childhood cancers and nearly 15% of pediatric cancer-related deaths (2). The disease is characterized by marked biological heterogeneity and strong metastatic potential (3). More than half of affected children present with metastasis at diagnosis, and prognosis varies substantially across risk groups. Children in low- and intermediate-risk groups generally have favorable outcomes, whereas the overall survival rate for high-risk patients remains poor (4). Accordingly, the burden experienced by children and families differs markedly by disease risk group.

Standard treatment for high-risk NB requires multiple sequential modalities, including induction chemotherapy, surgery, consolidation therapy, and maintenance therapy, and the entire process often lasts for several years (5). The disease mainly affects children younger than 15 years (6), and more than 90% of cases occur in children younger than 5 years (7). Because young children have limited capacity for self-care, their treatment trajectory is highly dependent on family caregiving. Beyond direct medical costs, families face long-term caregiving demands, psychological distress, educational interruption, and social disruption. These pressures can amplify the overall burden at the household and social levels (8).

Existing disease burden assessment tools are not well suited to this population. Generic caregiver burden scales, such as the Zarit Burden Interview and the Caregiver Burden Inventory, were mainly developed for caregivers of people with chronic diseases (9) and do not adequately capture uncertainty in pediatric rare disease treatment, developmental consequences, school interruption, or the family-centered nature of childhood cancer care (10). Many studies of NB have focused on direct medical expenditure, hospitalization, late effects, or quality of life as isolated dimensions (11, 12), with limited integration of family and social burden. Because of the lack of systematic assessment tools and the persistence of a predominantly biomedical model, hidden burdens such as caregiver emotional exhaustion, social support deficits, and policy-related access barriers may be overlooked (13). In addition, existing childhood cancer tools rarely incorporate macro-level health-policy indicators, such as medical insurance payment intensity, cross-provincial referral, and access to special drugs.

The Social Determinants of Health (SDH) framework emphasizes that health outcomes are shaped by interactions among biological and psychosocial factors, health care, socioeconomic position, living environment, social support, and policy context (14). This framework can help overcome the limitations of single-dimensional disease assessment and help identify upstream and downstream drivers of burden (15, 16). In this study, health insurance policy and medical access were linked to social burden, family socioeconomic status and support resources were linked to family burden, and physiological and psychological consequences were linked to personal burden. Guided by this theoretical structure, we used literature review, structured expert panel discussion, Delphi consultation, and the Analytic Hierarchy Process (AHP) to construct a multidimensional disease burden assessment system for childhood NB. The system is intended to provide an evidence-based tool for identifying high-burden populations, developing tiered intervention strategies, and optimizing allocation of health and social resources. Following its usage in rare disease and caregiver research (18–20), the term “disease burden” in this study refers to the multidimensional disease-related burden experienced by children and their families across treatment, economic, caregiving, and psychosocial dimensions, rather than to population-level health loss quantified by disability-adjusted life years (DALYs) (17). Based on the SDH framework, the system provides a theory-driven, multidimensional, and NB-specific assessment structure that links policy context, family socioeconomic circumstances, and child health outcomes.

## 2 Materials and methods

The indicator system was developed through four consecutive and traceable stages (1): structured extraction of literature evidence and mapping to the SDH theoretical framework (2); a structured expert panel discussion (3); two rounds of Delphi expert consultation; and (4) weighting with the Analytic Hierarchy Process (AHP). Details of each stage are given below; the corresponding raw materials are in **Appendix A1–A5** and the Literature Summary Table.

### 2.1 Literature review

A structured, indicator-generating literature review was performed to construct the initial indicator pool; no effect-size pooling was conducted. Chinese and English databases were searched, including CNKI, Wanfang Data, Chinese Biomedical Literature Service System (SinoMed), Ovid MEDLINE, Web of Science, EBSCO, and Embase. The search covered publications from January 1, 2001, to October 31, 2025. Search terms included combinations of neuroblastoma, olfactory neuroblastoma, pediatric, newborn, infant, disease burden, cost of illness, therapeutic burden, clinical burden, economic burden, quality of life, family burden, and social burden. Boolean operators were used to construct multidimensional search strategies. After title, abstract, and full-text screening, 81 of the 6,922 retrieved records were included. The complete search strategies and retrieval records for all seven databases are provided in **Appendix A1**, and the search report follows the PRISMA-S checklist. Structured information, including study characteristics, disease-related impact indicators, measurement tools, and indicator definitions, was extracted from each included study. The extracted concepts were inductively coded and mapped to structural determinants, intermediary determinants, cross-cutting social cohesion and social capital domains, or health and well-being outcomes according to the SDH conceptual framework (14), and candidate indicators were integrated to form a preliminary SDH-based assessment framework. The study-by-study extraction, SDH mapping, and candidate-item traceability are provided in the Literature Summary Table. (**Figures 1**, **2**).

**Figure 1:**
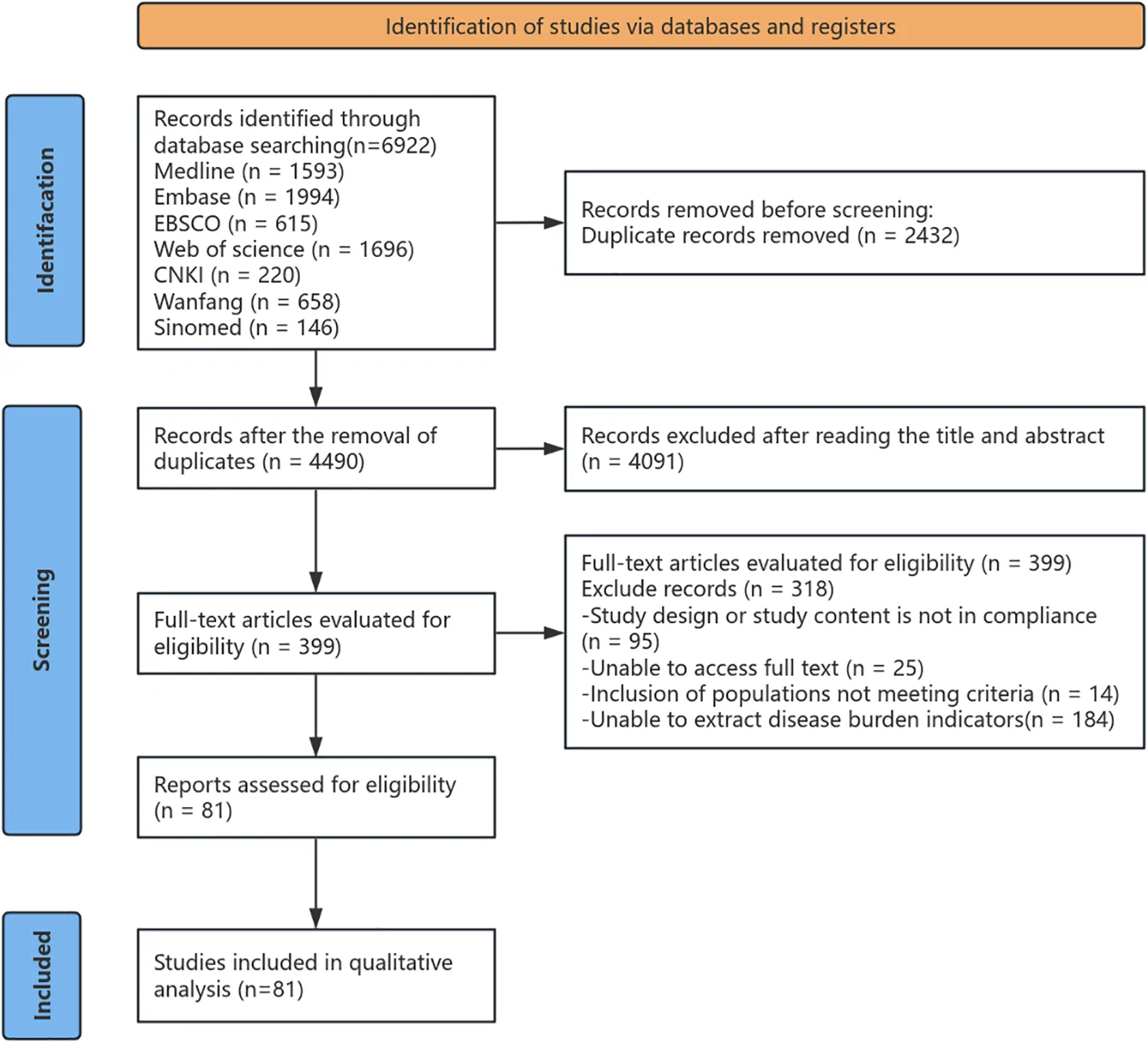
Flow diagram of literature screening. The initial database search identified 6,922 records. After title, abstract, and full-text screening, 81 studies relevant to the construction of the disease burden assessment system were included.

**Figure 2:**
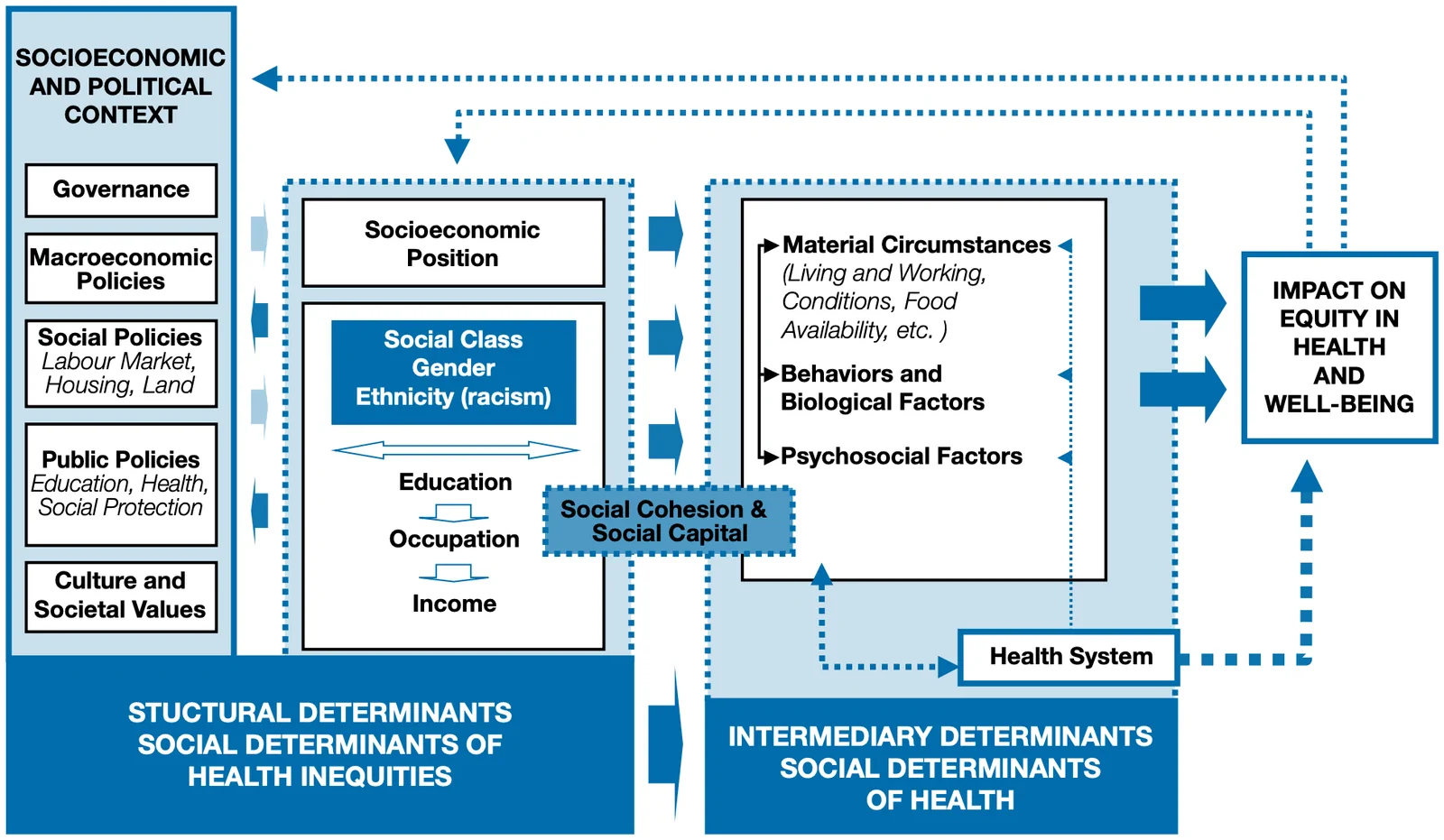
Conceptual framework for action on the social determinants of health (SDH). Reproduced from Solar O, Irwin A. A conceptual framework for action on the social determinants of health. Social Determinants of Health Discussion Paper 2 (Policy and Practice), Figure A, page 6. © World Health Organization 2010. Available at: https://iris.who.int/handle/10665/44489 (accessed September 8, 2026). Reproduced with permission of the World Health Organization. This figure is not covered by the Creative Commons licence of this article.

### 2.2 Structured expert panel discussion

A structured expert panel discussion was held in person on November 13, 2025, to review and refine the initial indicators. This process was a structured content-synthesis procedure rather than a formal qualitative focus group study. The discussion group included six members of the research team and four invited experts: two pediatric NB diagnosis and treatment experts, one pediatric NB nursing expert, and one health policy expert. The four invited experts had 15–29 years of professional experience (22.00 ± 5.94 years) and all held associate senior or senior professional titles. Before the discussion, the topic and preliminary assessment system were distributed to all participants. Participants commented on the relevance, hierarchical assignment, duplication, operability, and definitional clarity of candidate indicators. The meeting was audio-recorded in full and transcribed verbatim; two researchers also took on-site notes, and expert opinions were organized item by item after cross-checking the transcript against the notes. No formal thematic analysis or theoretical saturation assessment was performed. The meeting protocol, participant composition, and indicator change log are provided in **Appendix A2**. This process yielded a preliminary indicator system containing three first-level indicators, six second-level indicators, and 33 third-level indicators.

### 2.3 Delphi expert consultation

Two rounds of anonymous Delphi expert consultation were conducted from November to December 2025 via email and WeChat. Twenty-one experts were invited, and 20 agreed to participate (95.23%). Experts were selected according to professional balance and representativeness and included specialists in pediatric NB medicine or nursing, medical management, public health, health policy, and disease burden research. Inclusion criteria for nursing and medical management experts were an intermediate or higher professional title, a bachelor’s degree or higher, and at least 15 years of continuous work in the relevant field. Inclusion criteria for public health experts were a master’s degree or higher and at least 5 years of continuous work in the relevant field. Other experts were required to hold an associate senior or higher professional title, a master’s degree or higher, and at least 10 years of relevant experience. All experts participated voluntarily. The original questionnaires of both rounds are provided in **Appendix A3**, and the round-by-round scores and expert opinion processing are provided in **Appendix A4**.

The consultation questionnaire consisted of three parts. The first part described the study background, purpose, content, and instructions. The second part contained the SDH-based childhood NB disease burden assessment system and asked experts to rate the importance of each first-level indicator and the importance and feasibility of each second- and third-level indicator. Ratings were assigned using a 5-point Likert scale. Importance was scored from very unimportant to very important, and feasibility was scored from completely infeasible to completely feasible, with values from 1 to 5. Experts could revise wording, suggest deletion or addition of indicators, and provide comments. The third part collected general expert information, familiarity with the topic, and judgment basis. The assignment criteria for judgment basis and familiarity are shown in **Tables 1**, **2**.

**Table 1:** Expert judgment basis and degree assignment.

Judgment basis	High	Moderate	Low
Work experience	0.3	0.2	0.1
Theoretical analysis	0.5	0.4	0.3
Reference to domestic and international literature	0.1	0.1	0.1
Intuitive judgment	0.1	0.1	0.1

**Table 2:** Assignment for expert familiarity with item content.

Familiarity	Very familiar	Relatively familiar	Generally familiar	Not very familiar	Unfamiliar
Assignment	1.0	0.8	0.5	0.2	0

The two consultation rounds were separated by 2 weeks. After the first round, data cleaning and statistical analysis were performed, and quantitative ratings were combined with qualitative comments. Indicators were revised through research team discussion, including addition of necessary indicators, deletion of overlapping content, refinement of ambiguous wording, and reclassification of relevant indicators. Indicators were reconsidered if the mean importance or feasibility score was below 3.5 or if the coefficient of variation exceeded 0.25 (21). Expert enthusiasm was evaluated by questionnaire response rate and comment rate (22). Expert authority was expressed by the authority coefficient (Cr), calculated as Cr = (Ca + Cs)/2, where Ca denotes judgment basis and Cs denotes familiarity; Cr ≥ 0.70 indicates acceptable authority (23). Expert coordination was assessed using the coefficient of variation and Kendall’s coefficient of concordance (Kendall’s W), with values closer to 1 indicating stronger agreement (24).

### 2.4 Analytic hierarchy process

AHP was used to calculate indicator weights with Yaahp 12.13 software. First, a hierarchical structure model was constructed. The target layer was the development of an SDH-based multidimensional disease burden assessment system for childhood NB. The criterion layer I included three first-level indicators, criterion layer II included eight second-level indicators, and the scheme layer included 32 third-level indicators. Second, judgment matrices were constructed using the mean importance scores from the second Delphi round and Saaty’s 1–9 scale for pairwise comparisons. The detailed rule used to convert mean importance scores into Saaty scale values, together with the 12 judgment matrices and their weights and consistency ratios, is provided in **Appendix A5**. Third, weights were calculated and consistency testing was performed. A consistency ratio (CR) < 0.10 indicated acceptable logical consistency (25). Finally, global weights were calculated by multiplying the weights across hierarchical levels.

### 2.5 Statistical analysis

All data were entered into Excel with double checking and analyzed using SPSS 27.0. Descriptive statistics included means, standard deviations, coefficients of variation, and full-score rates. Kendall’s W, chi-square values, and P values were calculated to assess expert coordination. A two-sided P < 0.05 was considered statistically significant.

## 3 Results

### 3.1 Primary outcomes: composition and highest-weighted domains of the assessment system

The final assessment system comprised three first-level indicators, eight second-level indicators, and 32 third-level indicators. Among the first-level dimensions, family disease burden had the highest global weight (0.5278), followed by personal disease burden (0.3325) and social disease burden (0.1396). Among the second-level dimensions, family economic impact (0.2786), treatment-related burden (0.1755), and family caregiving burden (0.1755) ranked highest. The third-level indicators with the highest global weights were family out-of-pocket medical expenditure (0.1358), emotional stress of caregivers (0.0778), and surgery- or radiotherapy-related complications (0.0657). These figures are relative priority weights derived from the expert judgment matrices. The complete results for all 32 indicators are presented in **Table 3**. Taken together, these weights indicate that family-level determinants, particularly family economic impact and caregiving burden, were the most impactful SDH for families, whereas treatment-related burden was the most impactful domain for children.

**Table 3:** Multidimensional disease burden assessment system for children with neuroblastoma.

Dimension/indicator and operational definition	Importance mean ± SD	Importance CV	Feasibility mean ± SD	Feasibility CV	Global weight	Local weight	CR	Rank by global weight
A. Personal disease burden	4.90 ± 0.31	0.06	/	/	0.3325	0.3325	0.0516	
A1. Physiological health burden	4.85 ± 0.49	0.10	4.70 ± 0.73	0.16	0.1106	0.3325	0.0426	
A1.1 Growth and developmental status: height and weight; height-for-age Z score (HAZ), weight-for-age Z score (WAZ), and weight-for-height Z score (WHZ) evaluated using WHO standards	4.80 ± 0.52	0.11	4.80 ± 0.41	0.09	0.0239	0.2166		**13**
A1.2 Physical functioning: walking, exercise, self-care activities, fatigue, and physical symptoms assessed with the Pediatric Quality of Life Inventory (PedsQL) for children aged 2–18 years	4.80 ± 0.41	0.09	4.60 ± 0.50	0.11	0.0239	0.2166		**13**
A1.3 Tumor staging: International Neuroblastoma Staging System (INSS) assessment of primary tumor extent, midline crossing, lymph node involvement, distant metastasis, and disease progression	4.85 ± 0.37	0.08	4.60 ± 0.50	0.11	0.0343	0.3104		**11**
A1.4 Frequency and severity of pain episodes: pain episodes within 1 month, stratified as 0, 1-2, 3-4, or more episodes per month; age-adapted behavioral observation and self-report tools for pain severity	4.65 ± 0.49	0.11	4.35 ± 0.88	0.20	0.0125	0.1132		**25**
A1.5 Number of comorbidities: number of concurrent conditions such as cardiovascular or neurological diseases	4.50 ± 0.61	0.13	4.60 ± 0.60	0.13	0.0068	0.0619		**30**
A1.6 Survival status: survival outcome and prognosis of children with neuroblastoma	4.60 ± 0.60	0.13	4.40 ± 0.75	0.17	0.0090	0.0813		**27**
A2. Treatment-related burden	4.90 ± 0.31	0.06	4.60 ± 0.75	0.16	0.1755	0.5278	0.0573	
A2.1 Total treatment duration: total time from diagnosis to completion of core treatment	4.65 ± 0.49	0.11	4.65 ± 0.59	0.13	0.0236	0.1343		**15**
A2.2 Treatment complexity: number of treatment modalities in the regimen, including surgery, chemotherapy, radiotherapy, targeted therapy, immunotherapy, and stem cell transplantation	4.75 ± 0.44	0.09	4.40 ± 0.68	0.15	0.0499	0.2845		**8**
A2.3 Hospitalization frequency: number of hospital admissions due to neuroblastoma and complications within 1 year, stratified as 0, 1-2, or >=3 admissions	4.55 ± 0.60	0.13	4.75 ± 0.44	0.09	0.0127	0.0726		**24**
A2.4 Incidence of chemotherapy-related adverse reactions: nausea, vomiting, alopecia, myelosuppression, neurotoxicity, and other chemotherapy-induced adverse events	4.65 ± 0.49	0.11	4.55 ± 0.60	0.13	0.0236	0.1343		**15**
A2.5 Incidence of surgery- or radiotherapy-related complications: postoperative infection, bleeding, organ dysfunction, radiation-induced skin reactions, and related complications	4.80 ± 0.41	0.09	4.70 ± 0.47	0.10	0.0657	0.3742		3
A3. Psychosocial adaptation burden	4.65 ± 0.49	0.11	4.30 ± 0.86	0.20	0.0464	0.1396	0.0806	
A3.1 Emotional functioning: fear, sadness, sleep problems, and emotional status assessed using PedsQL for children aged 2–18 years	4.70 ± 0.57	0.12	4.30 ± 0.80	0.19	0.0224	0.4827		**17**
A3.2 Social functioning: peer interaction and social performance assessed using PedsQL for children aged 2–18 years	4.55 ± 0.60	0.13	4.40 ± 0.68	0.15	0.0128	0.2756		**23**
A3.3 Kindergarten or school enrollment status: non-enrollment, school absence, or suspension due to illness	4.45 ± 0.60	0.14	4.55 ± 0.69	0.15	0.0066	0.1412		**31**
A3.4 Kindergarten or school performance: school functioning and illness-related absence assessed with PedsQL among enrolled children	4.40 ± 0.60	0.14	4.25 ± 0.64	0.15	0.0047	0.1006		**32**
B. Family disease burden	4.95 ± 0.22	0.05	/	/	0.5278	0.5278	0.0516	
B1. Family economic impact	4.85 ± 0.37	0.08	4.60 ± 0.82	0.18	0.2786	0.5278	0.0579	
B1.1 Family out-of-pocket medical expenditure: annual out-of-pocket disease-related costs, including treatment, physician, inpatient, examination, and medication costs	4.95 ± 0.22	0.05	4.75 ± 0.44	0.09	0.1358	0.4874		1
B1.2 Transportation and accommodation costs for medical visits: travel costs calculated by visit frequency and single-visit expense; accommodation costs for cross-regional care calculated by hospitalization days and daily lodging costs; rental costs incurred for medical access	4.55 ± 0.60	0.13	4.60 ± 0.50	0.11	0.0266	0.0956		**12**
B1.3 Caregiver productivity loss: income loss due to work absence or unemployment caused by caregiving, calculated as caregiving days multiplied by caregiver daily income	4.80 ± 0.41	0.09	4.25 ± 0.64	0.15	0.0581	0.2085		**4**
B1.4 Incidence of catastrophic health expenditure: proportion of households in which out-of-pocket medical expenditure accounts for >=40% of disposable household income	4.70 ± 0.47	0.10	4.30 ± 0.66	0.15	0.0581	0.2085		**4**
B2. Family caregiving burden	4.80 ± 0.41	0.09	4.80 ± 0.41	0.09	0.1755	0.3325	0.0227	
B2.1 Average daily caregiving time: daily hours spent by caregivers on symptom management, treatment assistance, and daily care for children with neuroblastoma	4.65 ± 0.49	0.11	4.30 ± 0.73	0.17	0.0215	0.1222		**18**
B2.2 Restriction of caregivers’ independent life: assessed using the Zarit Caregiver Burden Interview, including loss of leisure, social activities, and lifestyle changes due to caregiving	4.85 ± 0.37	0.08	4.35 ± 0.75	0.17	0.0548	0.3121		**7**
B2.3 Health impact on caregivers: assessed using the Zarit Caregiver Burden Interview to determine whether caregiving affects caregiver health	4.65 ± 0.49	0.11	4.50 ± 0.51	0.11	0.0215	0.1222		**18**
B2.4 Emotional stress: assessed using the Zarit Caregiver Burden Interview, including anxiety, depression, fatigue, helplessness, emotional exhaustion, and persistent stress from long-term and complex neuroblastoma care	4.90 ± 0.31	0.06	4.20 ± 0.70	0.17	0.0778	0.4435		2
B3. Social support and resource access	4.50 ± 0.69	0.15	4.20 ± 1.01	0.24	0.0737	0.1396	0.0516	
B3.1 Socioeconomic support beyond medical insurance: availability of financial support outside medical insurance, including major disease subsidies, charitable aid, social donations, crowdfunding, and cross-regional medical subsidies	4.75 ± 0.44	0.09	4.60 ± 0.68	0.15	0.0438	0.5936		**9**
B3.2 Emotional and psychological support: professional psychological interventions, peer support networks, and support from relatives, friends, or neighbors for caregiver anxiety, depression, self-blame, and child psychological trauma	4.55 ± 0.60	0.13	4.05 ± 0.76	0.19	0.0184	0.2493		**21**
B3.3 Unmet social support needs: unmet material, emotional, or informational support needs	4.35 ± 0.67	0.15	4.10 ± 0.85	0.21	0.0116	0.1571		**26**
C. Social disease burden	4.40 ± 0.75	0.17	/	/	0.1396	0.1396	<0.0001	
C1. Accessibility of healthcare services	4.65 ± 0.59	0.13	4.40 ± 0.75	0.17	0.1047	0.7500	0.0516	
C1.1 Access to treatment and medications: whether tumor-specific treatments such as chemotherapy, immunotherapy, or drug therapy were not received, and reasons for non-receipt	4.75 ± 0.55	0.12	4.35 ± 0.75	0.17	0.0553	0.5278		**6**
C1.2 Cross-provincial referral: referral across provinces because diagnosis could not be confirmed or relevant treatment was unavailable; classified as active or passive referral, with diagnostic or treatment need as the core reason	4.35 ± 0.67	0.15	4.40 ± 0.68	0.15	0.0146	0.1396		**22**
C1.3 Diagnostic delay: interval from first symptom to confirmed diagnosis; based on prior literature, a diagnostic interval of >=1 month was defined as diagnostic delay	4.55 ± 0.69	0.15	4.55 ± 0.69	0.15	0.0348	0.3325		**10**
C2. Socioeconomic impact	4.30 ± 0.73	0.17	4.15 ± 0.75	0.18	0.0349	0.2500	<0.0001	
C2.1 Average annual total diagnostic and treatment cost: total annual medical expenditure incurred by the patient due to disease	4.65 ± 0.59	0.13	4.55 ± 0.83	0.18	0.0070	0.2000		**28**
C2.2 Intensity of medical insurance fund payment: proportion of total treatment costs paid by the medical insurance fund, calculated as fund payment divided by total treatment cost multiplied by 100%	4.85 ± 0.49	0.10	4.60 ± 0.82	0.18	0.0209	0.6000		**20**
C2.3 Proportion of insurance expenditure for special drugs and treatments: proportion of reimbursement for costly drugs or special treatments such as stem cell transplantation, immunotherapy, and DFMO (difluoromethylornithine)	4.65 ± 0.59	0.13	4.40 ± 0.99	0.23	0.0070	0.2000		**28**

CV, coefficient of variation; CR, consistency ratio; NB, neuroblastoma; PedsQL, Pediatric Quality of Life Inventory; WHO, World Health Organization; INSS, International Neuroblastoma Staging System; DFMO, difluoromethylornithine. Global weight denotes the weight of an indicator in the overall system, whereas local weight denotes the relative weight within its immediate parent indicator. The three third-level indicators with the highest global weights are shown in bold and shaded; the last column ranks all third-level indicators by global weight.

### 3.2 Expert characteristics

Of the 21 experts invited, 20 participated in the two Delphi rounds. Among them, 6 were male and 14 were female. Ages ranged from 35 to 64 years (47.65 ± 7.94 years), and years of professional experience ranged from 7 to 41 years (23.70 ± 10.07 years). The expert group covered clinical medicine, pediatric hematology and oncology, rare diseases, pediatric hematology and oncology nursing, public health, disease burden, health policy, and medical management (**Table 4**).

**Table 4:** General characteristics of consulting experts (n=20).

Item	Category	n	%	Item	Category	n	%
Age	30–39 years	2	10.00	Sex	Male	6	30.00
	40–49 years	10	50.00		Female	14	70.00
	>=50 years	8	40.00	Years of work	<10 years	1	5.00
Education	Bachelor’s degree	8	40.00		10–19 years	5	25.00
	Master’s degree	4	20.00		20–29 years	8	40.00
	Doctoral degree	8	40.00		>=30 years	6	30.00
Professional field	Clinical medicine (pediatric hematology/oncology and rare diseases)	6	30.00	Professional title	Intermediate	2	10.00
	Nursing (pediatric hematology/oncology)	7	35.00		Associate senior	9	45.00
	Public health and disease burden	3	15.00		Senior	9	45.00
	Health policy and management	4	20.00				

### 3.3 Expert enthusiasm and authority

The effective questionnaire response rates for the two rounds were 95.23% (20/21) and 100.00% (20/20), respectively. The expert comment rates were 75.00% and 25.00%, respectively, indicating high participation enthusiasm. Across the two rounds, the expert judgment coefficient (Ca) was 0.940, the familiarity coefficient (Cs) was 0.830, and the authority coefficient (Cr) was 0.885, exceeding the commonly accepted threshold of 0.700. These results indicate good expert authority and support the credibility of the consultation process.

### 3.4 Concentration and coordination of expert opinions

In the first Delphi round, Kendall’s W values for the importance ratings of second- and third-level indicators were 0.452 and 0.123, respectively (both P < 0.05). For feasibility ratings, the corresponding values were 0.186 and 0.188, respectively (both P < 0.05). In the second round, Kendall’s W values for importance ratings of second- and third-level indicators were 0.203 and 0.118, respectively (both P < 0.05), and those for feasibility ratings were 0.236 and 0.121, respectively (both P < 0.05). The statistically significant W values indicate acceptable coordination among experts across the two rounds (**Table 5**).

**Table 5:** Kendall’s coefficient of concordance in two rounds of expert consultation.

Round	Indicator level	Number of indicators	Importance W	Importance χ²	Importance P	Feasibility W	Feasibility χ²	Feasibility P
Round 1	Second-level indicators	6	0.452	43.409	<0.001	0.186	18.631	0.002
Round 1	Third-level indicators	33	0.123	78.804	<0.001	0.188	120.630	<0.001
Round 2	Second-level indicators	8	0.203	28.357	<0.001	0.236	33.029	0.002
Round 2	Third-level indicators	32	0.118	72.852	<0.001	0.121	75.248	<0.001

### 3.5 Indicator screening and final assessment system

After the first Delphi round, the research team added three second-level indicators and six third-level indicators, including treatment-related burden and tumor staging; deleted one second-level indicator and five third-level indicators, such as child education and social support, and distress caused by insufficient disease cognition; reclassified eight third-level indicators, such as assigning treatment complexity to treatment-related burden; and modified the wording of three second-level indicators and 17 third-level indicators. For example, personal time loss was revised to restriction of caregivers’ independent life, and frequency and duration of medical visits was revised to hospitalization frequency. Some operational definitions were also refined. For instance, diagnostic delay was defined as an interval of at least 1 month from first symptom onset to confirmed diagnosis based on previous literature (26, 27).

After the second Delphi round, no indicators were added or deleted. Three second-level indicators and one third-level indicator were revised for clarity; for example, psychosocial functional impact was revised to psychosocial adaptation burden. The definition of cross-provincial referral was refined as referral across provinces due to failure to confirm diagnosis or inability to obtain relevant treatment, classified as active or passive referral, with the core referral reason specified as diagnostic or treatment need. The final assessment system included three first-level indicators, eight second-level indicators, and 32 third-level indicators (**Table 3**).

### 3.6 Indicator weights

The CR for the target layer was 0.0516, and CR values for all criterion layers ranged from <0.0001 to 0.0806, all below the 0.10 threshold and indicating acceptable consistency of the judgment matrices. In addition to the highest-weighted indicators summarized above, caregiver productivity loss (global weight 0.0581), catastrophic health expenditure (0.0581), and access to treatment and medications (0.0553) ranked fourth to sixth among the third-level indicators. The complete global and local weights for all indicators are presented in **Table 3**, (**Figure 3**).

**Figure 3:**
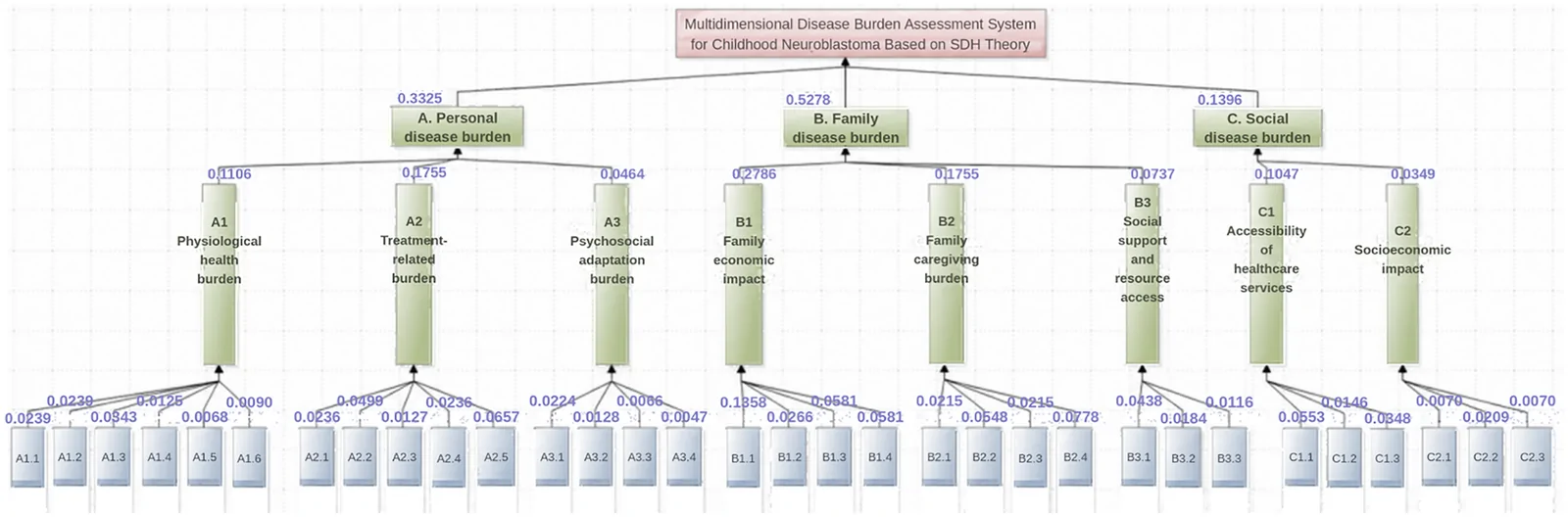
Hierarchical structure model of the multidimensional disease burden assessment system. The target layer was the SDH-based assessment system for childhood neuroblastoma; criterion layer I included personal, family, and social disease burden; criterion layer II included eight domains; and the scheme layer included 32 third-level indicators.

## 4 Discussion

### 4.1 Integration with the SDH framework

This study developed a multidimensional disease burden assessment system for childhood NB using the SDH framework. The system integrates policy environment, family socioeconomic context, and child health status into a single assessment structure. Social disease burden primarily corresponds to policy and healthcare access determinants, such as medical insurance fund payment intensity. Family disease burden primarily corresponds to household socioeconomic conditions and social support, such as caregiver productivity loss, emotional stress, and socioeconomic support beyond medical insurance. Personal disease burden primarily corresponds to direct physiological and psychosocial consequences, such as pain, tumor staging, and emotional functioning. Recent evidence from pediatric oncology is consistent with this mapping: a systematic review and meta-analysis reported associations between social determinants of health and outcomes in childhood, adolescent, and young adult cancer (28), and a Children’s Oncology Group analysis found that Black and Hispanic children with high-risk NB experienced inferior overall survival despite standardized frontline treatment (29). This mapping supports the applicability of the SDH framework in pediatric oncology and extends disease burden assessment beyond conventional clinical indicators (14, 31). As noted above, “disease burden” in this system refers to the multidimensional disease-related burden experienced at the patient and family levels, rather than population-level health loss in the DALY/QALY sense (17, 18).

The weight distribution underscores the central role of family disease burden. The global weight of family disease burden was 0.5278, which exceeded those of the personal and social disease burden dimensions. In line with this pattern, household poverty has been associated with inferior event-free and overall survival among children with high-risk NB receiving immunotherapy in cooperative group trials (30). This result is consistent with the SDH perspective that socioeconomic context and support structures exert decisive influence on health outcomes (32). Children with NB often require intensive, long-term, and complex treatment, and the family is the primary unit that absorbs financial costs, care labor, medical decision-making pressure, and psychosocial stress. Therefore, an assessment framework that underestimates the family dimension would fail to capture the true burden experienced in childhood NB.

### 4.2 Interpretation of high-weight family indicators

Caregiver emotional stress had the highest local weight within the family caregiving burden dimension. This finding suggests that psychological and emotional strain may be as consequential as direct economic expenditure. NB is one of the most clinically challenging pediatric tumors, given its aggressive course, recurrence risk, prolonged treatment, and potential pain burden. Caregivers may experience persistent anxiety, helplessness, sleep disturbance, self-blame, and trauma-related symptoms. If emotional exhaustion leads caregivers to withdraw from care or become unable to coordinate treatment, treatment continuity may be threatened. This result supports the SDH view that insufficient social and psychological support at the meso level can severely constrain household coping capacity (14).

The high weights assigned to family out-of-pocket medical expenditure, caregiver productivity loss, and catastrophic health expenditure further indicate that economic indicators remain essential. However, the system does not reduce burden to medical costs alone. Instead, it jointly captures direct medical spending, indirect income loss, caregiving time, psychological pressure, and unmet support needs. This multidimensional approach is particularly relevant for rare pediatric diseases, where families frequently encounter high uncertainty, prolonged care trajectories, and limited access to disease-specific resources (33, 34).

### 4.3 Comprehensiveness and specialty-specific characteristics

The final system contained three first-level, eight second-level, and 32 third-level indicators. Compared with generic burden tools, the system incorporates clinical, economic, caregiving, psychosocial, health service, and policy dimensions. This structure reflects the transition from a purely biomedical model to a biopsychosocial and policy-informed model. It also preserves specialty-specific features of NB. For example, tumor staging, treatment complexity, chemotherapy-related adverse reactions, surgery- or radiotherapy-related complications, and access to special drugs and treatments are closely linked to NB treatment pathways and resource consumption. These indicators help distinguish NB burden from that of general chronic diseases or adult cancers. These features correspond to three innovations of the system (1): it is grounded in the SDH framework, explicitly linking policy determinants to family and individual outcomes (2); it integrates specialty-specific clinical indicators, such as tumor staging, treatment complexity, and access to special drugs and treatments, with the economic and caregiving domains; and (3) its operational definitions were designed for the clinical reality of pediatric oncology in China, facilitating future adaptation for use by clinicians and health administrators.

The system also includes school or kindergarten enrollment status, school performance, and psychosocial adaptation. Because NB occurs predominantly in early childhood, disease and treatment may affect growth and development, emotional regulation, peer interaction, and education. These long-term developmental consequences are not fully reflected by survival outcomes or cost data. The system therefore provides a more comprehensive profile of disease burden across the life course of affected children.

### 4.4 Clinical and policy implications

The assessment system is designed to support a three-stage pathway of screening, intervention, and policy optimization. At the screening stage, clinicians and nurses can use high-weight family indicators, such as catastrophic health expenditure, caregiver productivity loss, and emotional stress, to identify families at risk of economic collapse or psychological exhaustion at admission or follow-up. This approach expands risk recognition from a single economic dimension to a combined economic-psychological model, reducing the likelihood of overlooking hidden high-burden families.

At the intervention stage, the system can guide individualized and tiered resource allocation. Families with prominent economic burden may be referred to medical social workers for charity assistance, major disease subsidies, secondary reimbursement, or temporary community relief. Families with high caregiver burden may benefit from respite care, psychological counseling, and peer support groups. Children with high personal burden, especially pain or psychosocial adaptation problems, may require multidisciplinary pain management and pediatric psychological care. Medical, nursing, social work, and community resources can thus be integrated into a closed-loop care model.

At the policy optimization stage, social burden indicators, such as medical insurance fund payment intensity and the proportion of insurance expenditure for special drugs and treatments, can help health administrators monitor whether insurance coverage and payment reforms effectively reduce household burden. If multicenter data show that certain targeted therapies remain associated with high catastrophic expenditure despite inclusion in insurance schemes, reimbursement ratios or special assistance funds may need adjustment. The system may also support evaluation of regional pediatric oncology center construction and guide resource allocation toward the most burden-intensive stages of care (35).

### 4.5 Limitations and future directions

This study has several limitations. First, although experts were recruited from multiple professional fields, their geographic distribution was relatively concentrated in eastern China (Jiangsu, Shanghai, and Zhejiang), which may limit representativeness. Regional differences in medical insurance policies and resource accessibility may affect the feasibility of certain indicators. Second, Delphi consultation inevitably involves subjective judgment, even though expert authority and coordination were acceptable. To mitigate subjectivity, we used anonymous rounds, quantitative inclusion criteria (importance mean ≥ 3.5 and coefficient of variation ≤ 0.25), and the AHP, which structures pairwise comparisons. Third, the system has not yet undergone large-scale empirical validation, and some operational definitions require further refinement according to local clinical pathways, information systems, and policy contexts. Future studies should conduct multicenter validation, enroll patients and caregivers to complete item refinement and scoring-system development, and test reliability, validity, responsiveness, and measurement invariance in real-world populations before examining whether the system predicts clinical, economic, and psychosocial outcomes.

## 5 Conclusion

This study constructed an SDH-based multidimensional disease burden assessment system for childhood NB using literature review, structured expert panel discussion, Delphi consultation, and AHP. The final system contains personal, family, and social burden dimensions, with family burden receiving the highest global weight. The system is theory-grounded and based on expert consensus; it is a preliminary content model to inform future tool development. Before clinical or policy application, item reduction, scoring-system development, and psychometric and multicenter validation in children and families are required.

## Data Availability

The original contributions presented in the study are included in the article/**Supplementary Material**. Further inquiries can be directed to the corresponding author.
