# Supplementary material for: Development of a multidimensional disease burden assessment system for childhood neuroblastoma based on the social determinants of health theory
Source: Front Oncol. 2026 Sep 15;16:1886324. doi: 10.3389/fonc.2026.1886324 (PMC13619433; doi:10.3389/fonc.2026.1886324)
Supplement: Supplementary file 1 [file DataSheet1.pdf]

Appendix1

search report

一、Reference Sources for Search Strategy 2

二、Summary of Retrieval Results 2

三、Search Strategies by Database 3

Database 1 OVID MEDLINE（R） ALL 3

Database 2 Embase 4

Database 3 EBSCO 5

Database 4 Web of Science 6

Database 5 Wanfang Data 7

Database 6 CNKI 8

Database 7 SinoMed 10

一、Reference Sources for Search Strategy

1. YALçIN B, KREMER L C M, VAN DALEN E C. High- dose chemotherapy and autologous haematopoietic stem cell rescue for children with high- risk neuroblastoma [J]. Cochrane Database of Systematic Reviews, 2015, (10).DOI:10.1002/14651858.CD006301.pub4.

2. PEINEMANN F, VAN DALEN E C, ENK H, et al. Anti- GD2 antibody- containing immunotherapy postconsolidation therapy for people with high- risk neuroblastoma treated with autologous haematopoietic stem cell transplantation [J]. Cochrane Database of Systematic Reviews, 2019, (4).DOI:10.1002/14651858.CD012442.pub2.

3.BAJWAH S, OLUYASE A O, YI D, et al. The effectiveness and cost- effectiveness of hospital- based specialist palliative care for adults with advanced illness and their caregivers [J]. Cochrane Database of Systematic Reviews, 2020, (9).DOI:10.1002/14651858.CD012780.pub2.

二、Summary of Retrieval Results(Search period: 2001 to October 31, 2025; N = 6922)

No.	Database	Records Retrieved
1	OVID MEDLINE（R）ALL	1593
2	Embase	1994
3	EBSCO	615
4	Web of Science	1696
5	wanfang	658
6	CNKI	220
7	SinoMed	146

三、Search Strategies by Database

Database 1: OVID MEDLINE（R）ALL

Date: <2001 to October 31, 2025>

#	Search terms	Results
1	exp Infant, Newborn/ or exp Infant/ or exp Infant, Large for Gestational Age/ or exp Infant, Low Birth Weight/ or exp Infant, Small for Gestational Age/ or exp Infant, Very Low Birth Weight/ or exp Infant, Postmature/ or exp Infant, Premature/ or exp Infant, Extremely Premature/ or exp Infant, Extremely Low Birth Weight/ or exp Child/ or exp Child, Preschool/ or exp Adolescent/ or exp Pediatrics/ or exp Neonatology/	4243510
2	(Neonat* or Newborn* or Infant* or Child* or Teen* or Teenager* or Youth* or Pediatric* or Neonatolog* or Adolescen* or toddler* or schoolchild* or kid* or juvenil* or bab* or preteen* or "pre teen*" or son or daughter).ab,kw,ti.	3743363
3	(("High Birth" or LGA or "Large for" or "Low Birth") adj2 Weight*).ab,kw,ti.	26196
4	1 or 2 or 3	5840816
5	exp neuroblastoma/ or exp Esthesioneuroblastoma, Olfactory/ or exp Ganglioneuroblastoma/	33621
6	exp neuroblastoma/ or exp Esthesioneuroblastoma Olfactory/ or exp Ganglioneuroblastoma/	33621
7	(neuroblastoma* or Ganglioneuroblastoma* or "Ganglio neuroblastoma*" or Esthesioneuroblastoma* or "Esthesio neuroblastoma*" or Aesthesioneuroblastoma* or "Aesthesio neuroblastoma*" or neuroepitheliom* or schwannian).abkwti.	46314
8	(Neuroblast* adj1 (neoplasm* or tumor* or tumour* or cancer* or carcinoma* or malignant*)).abkwti.	3226
9	6 or 7 or 8	53351
10	5 and 9	18788
11	letter/ or editorial/ or news/ or exp historical article/ or Anecdotes as Topic/ or comment/ or case report/ or (letter or comment* or "case stud*" or "case report*").ti.	3502869
12	exp randomized controlled trial/ or random*.tiab.	1819697
13	11 not 12	3471119
14	(animals/ not humans/) or exp animals laboratory/ or exp animal experimentation/ or exp models animal/ or exp rodentia/ or (rat or rats or mouse or mice).ti.	5961221
15	13 or 14	9314230
16	10 not 15	16785
17	limit 16 to english language	14721
18	limit 17 to yr="2001-Current"	9417
19	((disease* or illness or sickness or Therapeutic or pill* or clinical or treatment or symptom* or nurs*) adj1 cost*).abkwti.	17577
20	exp "Cost of Illness"/	39134
21	burden*.abkwti.	418034
22	19 or 20 or 21	447787
23	18 and 22	186
24	(incidence or prevalence or mortality or "survival rate" or epidemiology or "direct costs" or "indirect costs" or "medical expenses" or "disease costs" or "disease expenditure" or "social costs" or "quality of life" or QoL or HRQoL or "disability-adjusted life years" or DALY or "years of life lost"	3487139

	or YLL or "years lived with disability" or YLD or "medical security").abkwti.	
25	18 and 24	1681
26	25 not 23	1593

Database 2: Embase

Date: <2001 to October 31, 2025>

#	Search terms	Results
1	'juvenile'/exp OR 'pediatrics'/exp OR 'child'/exp OR 'preschool child'/exp OR 'newborn'/exp OR 'small for gestational age'/exp	5,004,491
2	neonat*:ti,ab,kw OR newborn*:ti,ab,kw OR infant*:ti,ab,kw OR child*:ti,ab,kw OR teen*:ti,ab,kw OR teenager*:ti,ab,kw OR youth*:ti,ab,kw OR pediatric*:ti,ab,kw OR neonatolog*:ti,ab,kw OR adolescen*:ti,ab,kw OR toddler*:ti,ab,kw OR schoolchild*:ti,ab,kw OR kid*:ti,ab,kw OR juvenil*:ti,ab,kw OR bab*:ti,ab,kw OR preteen*:ti,ab,kw OR 'pre teen*':ti,ab,kw	5,036,486
3	(('high birth' OR lga OR 'large for' OR 'low birth' OR 'very low birth') NEAR/2 weight*):ti,ab,kw	51,972
4	(premature:ti,ab,kw OR postmature:ti,ab,kw OR 'extremely preterm':ti,ab,kw) AND near:ti,ab,kw AND infant*:ti,ab,kw	1,037
5	#1 OR #2 OR #3 OR #4	7,004,947
6	'neuroblastoma'/exp	46,412
7	'esthesioneuroblastoma, olfactory'/exp	2,987
8	'ganglioneuroblastoma'/exp	46,412
9	'congenital neuroblastoma'/exp	37
10	'olfactory neuroepithelioma'/exp OR 'neuroepithelioma'/exp	4,937
11	'neuroectoderm tumor'/exp	19,070
12	#6 OR #7 OR #8 OR #9 OR #10 OR #11	71,238
13	'neuroganglioblastoma':ti,ab,kw OR 'sympathicoblastoma':ti,ab,kw OR 'sympathicogonioma':ti,ab,kw OR 'sympathiconeuroblastoma':ti,ab,kw OR 'sympathoblastoma':ti,ab,kw OR 'sympathoma':ti,ab,kw OR 'sympaticoblastoma':ti,ab,kw OR neuroblastoma*:ti,ab,kw OR ganglioneuroblastoma*:ti,ab,kw OR esthesioneuroblastoma*:ti,ab,kw OR aesthesioneuroblastoma*:ti,ab,kw OR neuroepitheliom*:ti,ab,kw OR schwannian:ti,ab,kw OR ppnet:ti,ab,kw OR pnet*:ti,ab,kw	70,334
14	((neuroblast* OR neuroectoderma*) NEAR/1 (neoplasm* OR tumor* OR tumour* OR cancer* OR carcinoma* OR malignant*)):ti,ab,kw	10,655
15	#12 OR #13 OR #14	102,693
16	'disease burden'/exp	112,856
17	((disease* OR illness OR sickness OR therapeutic OR pill* OR clinical OR treatment OR symptom* OR nurs*) NEAR/1 cost*):ti,ab,kw	28,011
18	burden*:ti,ab,kw OR epidemiology:ti,ab,kw OR 'direct costs':ti,ab,kw OR 'indirect costs':ti,ab,kw OR 'medical expenses':ti,ab,kw OR 'disease expenditure':ti,ab,kw OR 'social costs':ti,ab,kw OR 'quality of life':ti,ab,kw OR qol:ti,ab,kw OR hrqol:ti,ab,kw OR 'medical security':ti,ab,kw	1,754,885
19	#16 OR #17 OR #18	5,661,295
20	#5 AND #15 AND #19	7,857

21	'letter'/exp OR 'editorial'/exp OR 'news'/exp OR 'anecdotes as topic'/exp OR 'case report'/exp OR letter:ti OR comment*:ti OR 'historical article':ti	5,513,523
22	'randomized controlled trial'/exp OR random*:ti,ab	2,684,292
23	#21 NOT #22	5,451,194
24	'animals'/exp NOT 'humans'/exp OR 'animals,laboratory'/exp OR 'animal experimentation'/exp OR 'models, animal'/exp OR rat:ti OR rats:ti OR mouse:ti OR mice:ti OR rodential:ti	7,601,236
25	#23 OR #24	12,954,719
26	#20 NOT #25	6,746
27	#26 AND [english]/lim	6,372
28	#27 AND (2001:py OR 2002:py OR 2003:py OR 2004:py OR 2005:py OR 2006:py OR 2007:py OR 2008:py OR 2009:py OR 2010:py OR 2011:py OR 2012:py OR 2013:py OR 2014:py OR 2015:py OR 2016:py OR 2017:py OR 2018:py OR 2019:py OR 2020:py OR 2021:py OR 2022:py OR 2023:py OR 2024:py OR 2025:py)	1,994

Database 3: EBSCO

Date: 2025 .10. 31

#	Search terms	Results
S4	S2 AND S3 AND S4	615
S3	SU (((disease* or illness or sickness or Therapeutic or pill* or clinical or treatment or symptom* or nurs*) near/1 cost*)) OR SU ((burden* or epidemiology OR “direct costs ” OR "indirect costs" OR "medical expenses" OR "disease expenditure" OR "social costs" OR "quality of life" OR QoL OR HRQoL OR "medical security"))	2,995,286
S2	SU ((neuroganglioblastoma OR sympathicoblastoma OR sympathicogonioma OR sympathiconeuroblastoma OR sympathoblastoma OR sympathoma OR sympaticoblastoma or neuroblastoma* or Ganglioneuroblastoma* or Esthesioneuroblastoma* or Aesthesioneuroblastoma* or neuroepitheliom* or schwannian or pPNET OR PNET*)) OR SU (((Neuroblast* or Neuroectoderma*) near/3 (neoplasm* or tumor* or tumour* or cancer* or carcinoma* or malignant*)))	45,475
S1	SU ((Neonat* or Newborn* or Infant* or Child* or Teen* or Teenager* or Youth* or Pediatric* or Neonatolog* or Adolescen* or toddler* or schoolchild* or kid* or juvenil* or bab* or preteen* or "pre teen*" or son or daughter)) OR SU ((("High Birth" or LGA or "Large for" or "Low Birth") near/2 Weight*)) OR SU (((Premature or Postmature or "Extremely Preterm") near/1 infant*))	7,535,038

Database 4： Web of Science

Date: 2001– 2025 .10 .31

#	Search terms	Results
1	TS=((Neonat* or Newborn* or Infant* or Child* or Teen* or Teenager* or Youth* or Pediatric* or Neonatolog* or Adolescen* or toddler* or schoolchild* or kid* or juvenil* or bab* or preteen* or "pre teen*" or son or daughter))	3,180,460
2	TS=((("High Birth" or LGA or "Large for" or "Low Birth") NEAR/2 Weight*))	35,922
3	TS=((Premature or Postmature or "Extremely Preterm") NEAR/1 infant*)	22,558
4	#1 OR #2 OR #3	3,185,753
5	TS=((neuroblastoma* or Ganglioneuroblastoma* or "Ganglio neuroblastoma*" or Esthesioneuroblastoma* or "Esthesio neuroblastoma*" or Aesthesioneuroblastoma* or "Aesthesio neuroblastoma*" or neuroepitheliom* or schwannian))	42,852
6	TS=(Neuroblast* NEAR/3 (neoplasm* or tumor* or tumour* or cancer* or carcinoma* or malignant*))	6,042
7	#5 OR #6	43,067
8	TS=((disease* or illness or sickness or Therapeutic or pill* or clinical or treatment or symptom* or nurs*) NEAR/3 cost*)	71,310
9	TS=(burden*)	451,945
10	#8 OR #9	512,125
11	#10 AND #7 AND #4	222
12	PY=(2001-2025)	44,883,097
13	TI=(letter or editorial or news or “historical article” or “Anecdotes as Topic” or comment or “case report”)	448,938
14	TS=("randomized controlled trial" or random*)	2,064,025
15	#13 NOT #14	441,478
16	TS=(animal* not human*)	843,789
17	TI=(rodential or rat* or mouse or mice)	1,658,826
18	#16 OR #17	2,353,989
19	#15 OR #18	2,788,316
20	#12 AND #11	218
21	#20 NOT #19	204
22	TS=(incidence OR prevalence OR mortality OR "survival rate" OR epidemiology OR "direct costs" OR "indirect costs" OR "medical expenses" OR "disease costs" OR "disease expenditure" OR "social costs" OR "quality of life" OR QoL OR HRQoL OR "disability-adjusted life years" OR DALY OR "years of life lost" OR YLL OR "years lived with disability" OR YLD OR "medical security")	3,544,546
23	#4 AND #7 AND #22	1948
24	LA=(English)	45,979,779
25	#24 AND #23 AND #12	1,720
26	#25 NOT #19	1,699
27	#26 NOT #21	1,696

Database 5: Wanfang Data

Date： 2025 . 10. 31

(主题:(儿童 or 小儿 or 婴幼儿 or 儿科 or 新生儿 or 新生儿 or 婴儿 or 高出生体重婴儿 or 大于胎龄儿 or 低出生体重儿 or 小于胎龄儿 or 极低出生体重儿 or 过期产儿 or 早产儿 or 极度早产儿 or 超低出生体重儿 or 学龄前儿童 or 青少年 or 新生儿学 or 幼儿 or 学龄儿童) and 主题:(神经母细胞瘤 or 神经节神经母细胞瘤 or 嗅觉神经母细胞瘤 or 嗅觉神经上皮瘤 or 神经上皮瘤 or 神经外胚层肿瘤 or 神经母细胞瘤病 or NB or 外周原始神经外胚层肿瘤 or PNET or 颅外原始神经外胚层肿瘤 or 外周神经外胚层肿瘤 or 神经节母细胞瘤 or 外周原始神经外胚层瘤 or 颅外原始神经外胚层瘤 or 周围神经外胚层瘤) and 主题:(负担 or 疾病负担 or 患病负担 or 发病率 or 患病率 or 死亡率 or 生存率 or 流行病学 or 经济负担 or 医疗费用 or 直接成本 or 间接成本 or 医疗保障 or 生活质量 or 生命质量 or QoL or HRQoL or 照护负担 or 家庭负担 or 疾病经济负担 or 疾病成本 or 疾病花费 or 治疗负担 or 用药负担 or 症状负担 or 临床负担 or 社会负担 or 照顾者负担 or 家庭经济负担 or 照护负担 or 心理负担 or 社会成本 or 残疾调整生命年 or DALY or 寿命损失年 or YLL or 健康寿命损失年 or YLD or 生存质量)) and 发表时间:2001-* 【期刊论文、学位论文】

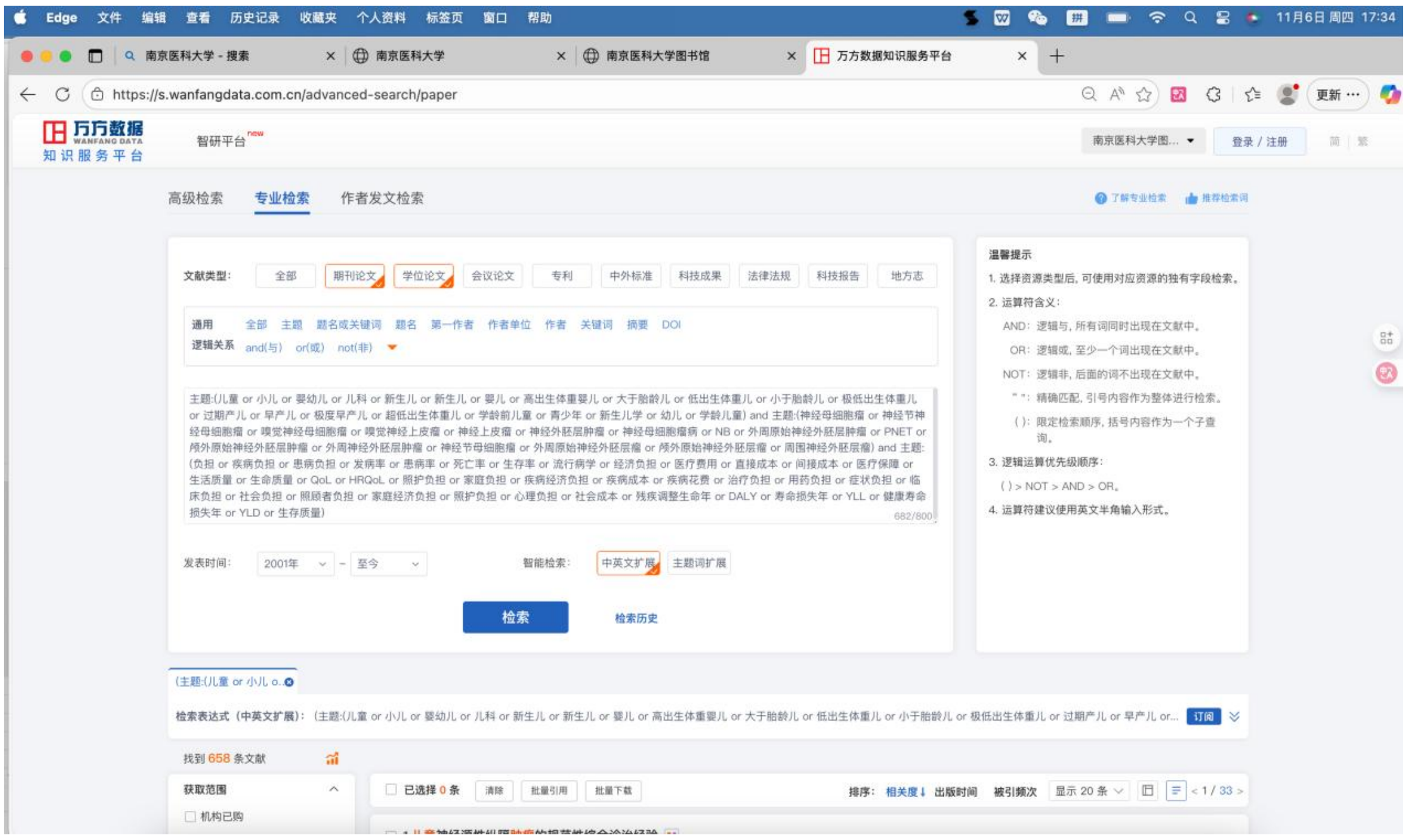

Database 6: C N K I

Date: 2025.11.6

SU=儿童 + 小儿 + 婴幼儿 + 儿科 + 新生儿 + 新生儿 + 婴儿 + 高出生体重婴儿 + 大于胎龄儿 + 低出生体重儿 + 小于胎龄儿 + 极低出生体重儿 + 过期产儿 + 早产儿 + 极度早产儿 + 超低出生体重儿 + 学龄前儿童 + 青少年 + 新生儿学 + 幼儿 + 学龄儿童 and SU=神经母细胞瘤 + 神经节神经母细胞瘤 + 嗅觉神经母细胞瘤 + 嗅觉神经上皮瘤 + 神经上皮瘤 + 神经外胚层肿瘤 + 神经母细胞瘤病 + NB + 外周原始神经外胚层肿瘤 + PNET + 颅外原始神经外胚层肿瘤 + 外周神经外胚层肿瘤 + 神经节母细胞瘤 + 外周原始神经外胚层瘤 + 颅外原始神经外胚层瘤 + 周围神经外胚层瘤 and SU=负担 + 疾病负担 + 患病负担 + 发病率 + 患病率 + 死亡率 + 生存率 + 流行病学 + 经济负担 + 医疗费用 + 直接成本 + 间接成本 + 医疗保障 + 生活质量 + 生命质量 + QoL + HRQoL + 照护负担 + 家庭负担 + 疾病经济负担 + 疾病成本 + 疾病花费 + 治疗负担 + 用药负担 + 症状负担 + 临床负担 + 社会负担 + 照顾者负担 + 家庭经济负担 + 照护负担 + 心理负担 + 社会成本 + 残疾调整生命年 + DALY + 寿命损失年 + YLL + 健康寿命损失年 + YLD + 生存质量 + 预后 【学术期刊+学位论文】

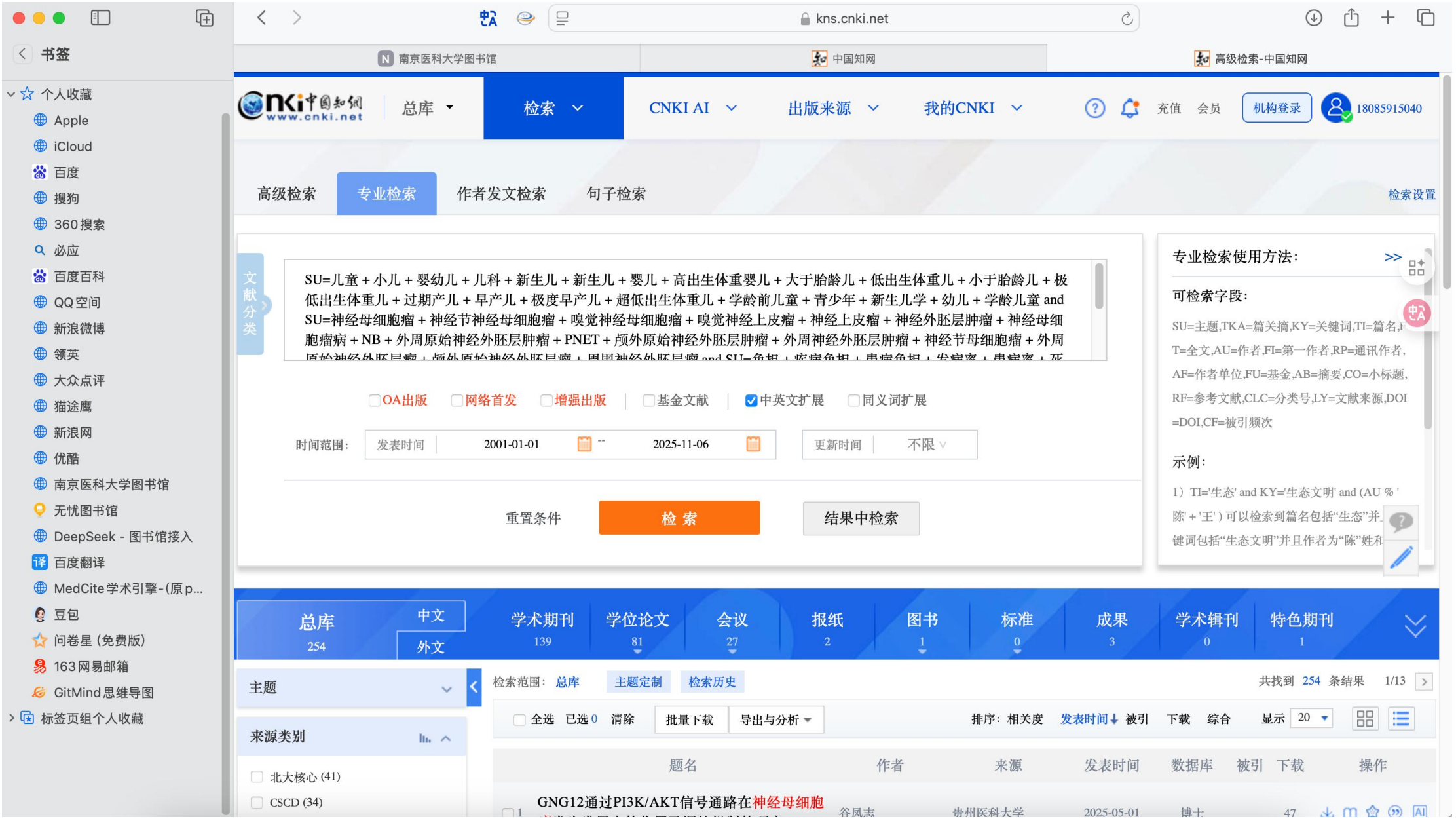
kns.cnki.net
书签
个人收藏
Apple
iCloud
百度
搜狗
360搜索
必应
百度百科
QQ空间
新浪微博
领英
大众点评
猫途鹰
新浪网
优酷
南京医科大学图书馆
无忧图书馆
DeepSeek - 图书馆接入
百度翻译
豆包
问卷星 (免费版)
163网易邮箱
GitMind思维导图
标签页组个人收藏
南京医科大学图书馆
中国知网
高级检索-中国知网
总库
检索
CNKI AI
出版来源
我的CNKI
充值
会员
机构登录
18085915040
高级检索
专业检索
作者发文检索
句子检索
检索设置
文献分类
SU=儿童 + 小儿 + 婴幼儿 + 儿科 + 新生儿 + 新生儿 + 婴儿 + 高出生体重婴儿 + 大于胎龄儿 + 低出生体重儿 + 小于胎龄儿 + 极低出生体重儿 + 过期产儿 + 早产儿 + 极度早产儿 + 超低出生体重儿 + 学龄前儿童 + 青少年 + 新生儿学 + 幼儿 + 学龄儿童 and SU=神经母细胞瘤 + 神经节神经母细胞瘤 + 嗅觉神经母细胞瘤 + 嗅觉神经上皮瘤 + 神经上皮瘤 + 神经外胚层肿瘤 + 神经母细胞瘤病 + NB + 外周原始神经外胚层肿瘤 + PNET + 颅外原始神经外胚层肿瘤 + 外周神经外胚层肿瘤 + 神经节母细胞瘤 + 外周
OA出版
网络首发
增强出版
基金文献
中英文扩展
同义词扩展
时间范围:
发表时间
2001-01-01
2025-11-06
更新时间
不限
重置条件
检 索
结果中检索
专业检索使用方法:
可检索字段:
示例:
总库
254
中文
外文
学术期刊
139
学位论文
81
会议
27
报纸
2
图书
1
标准
0
成果
3
学术辑刊
0
特色期刊
1
主题
来源类别
北大核心 (41)
CSCD (34)
检索范围:
总库
主题定制
检索历史
共找到
254
条结果
1/13
全选
已选
0
清除
批量下载
导出与分析
排序:
相关度
发表时间
被引
下载
综合
显示
20
题名
作者
来源
发表时间
数据库
被引
下载
操作
GNG12通过PI3K/AKT信号通路在神经母细胞
2025-05-01
47

Database 7：SinoMed

Date： 2024.7.30

((("儿童"[核心字段:智能] OR "小儿"[核心字段:智能] OR "婴幼儿"[核心字段:智能] OR "儿科"[核心字段:智能] OR "新生儿"[核心字段:智能] OR "新生儿"[核心字段:智能] OR "婴儿"[核心字段:智能] OR "高出生体重婴儿"[核心字段:智能] OR "大于胎龄儿"[核心字段:智能] OR "低出生体重儿"[核心字段:智能] OR "小于胎龄儿"[核心字段:智能] OR "极低出生体重儿"[核心字段:智能] OR "过期产儿"[核心字段:智能] OR "早产儿"[核心字段:智能] OR "极度早产儿"[核心字段:智能] OR "超低出生体重儿"[核心字段:智能] OR "学龄前儿童"[核心字段:智能] OR "青少年"[核心字段:智能] OR "新生儿学"[核心字段:智能] OR "幼儿"[核心字段:智能] OR "学龄儿童"[核心字段:智能]) AND("神经母细胞瘤"[核心字段:智能] OR "神经节神经母细胞瘤"[核心字段:智能] OR "嗅觉神经母细胞瘤"[核心字段:智能] OR "嗅觉神经上皮瘤"[核心字段:智能] OR "神经上皮瘤"[核心字段:智能] OR "神经外胚层肿瘤"[核心字段:智能] OR "神经母细胞瘤病"[核心字段:智能] OR "NB"[核心字段:智能] OR "外周原始神经外胚层肿瘤"[核心字段:智能] OR "PNET"[核心字段:智能] OR "颅外原始神经外胚层肿瘤"[核心字段:智能] OR "外周神经外胚层肿瘤"[核心字段:智能] OR "神经节母细胞瘤"[核心字段:智能] OR "外周原始神经外胚层瘤"[核心字段:智能] OR "颅外原始神经外胚层瘤"[核心字段:智能] OR "周围神经外胚层瘤"[核心字段:智能])) AND("负担"[核心字段:智能] OR "疾病负担"[核心字段:智能] OR "患病负担"[核心字段:智能] OR "发病率"[核心字段:智能] OR "患病率"[核心字段:智能] OR "死亡率"[核心字段:智能] OR "生存率"[核心字段:智能] OR "流行病学"[核心字段:智能] OR "经济负担"[核心字段:智能] OR "医疗费用"[核心字段:智能] OR "直接成本"[核心字段:智能] OR "间接成本"[核心字段:智能] OR "医疗保障"[核心字段:智能] OR "生活质量"[核心字段:智能] OR "生命质量"[核心字段:智能] OR "QoL"[核心字段:智能] OR "HRQoL"[核心字段:智能] OR "照护负担"[核心字段:智能] OR "家庭负担"[核心字段:智能] OR "疾病经济负担"[核心字段:智能] OR "疾病成本"[核心字段:智能] OR "疾病花费"[核心字段:智能] OR "治疗负担"[核心字段:智能] OR "用药负担"[核心字段:智能] OR "症状负担"[核心字段:智能] OR "临床负担"[核心字段:智能] OR "社会负担"[核心字段:智能] OR "照顾者负担"[核心字段:智能] OR "家庭经济负担"[核心字段:智能] OR "照护负担"[核心字段:智能] OR "心理负担"[核心字段:智能] OR "社会成本"[核心字段:智能] OR "残疾调整生命年"[核心字段:智能] OR "DALY"[核心字段:智能] OR "寿命损失年"[核心字段:智能] OR "YLL"[核心字段:智能] OR "健康寿命损失年"[核心字段:智能] OR "YLD"[核心字段:智能] OR "生存质量"[核心字段:智能])) AND (人类[特征词]) AND 2000-2025[日期

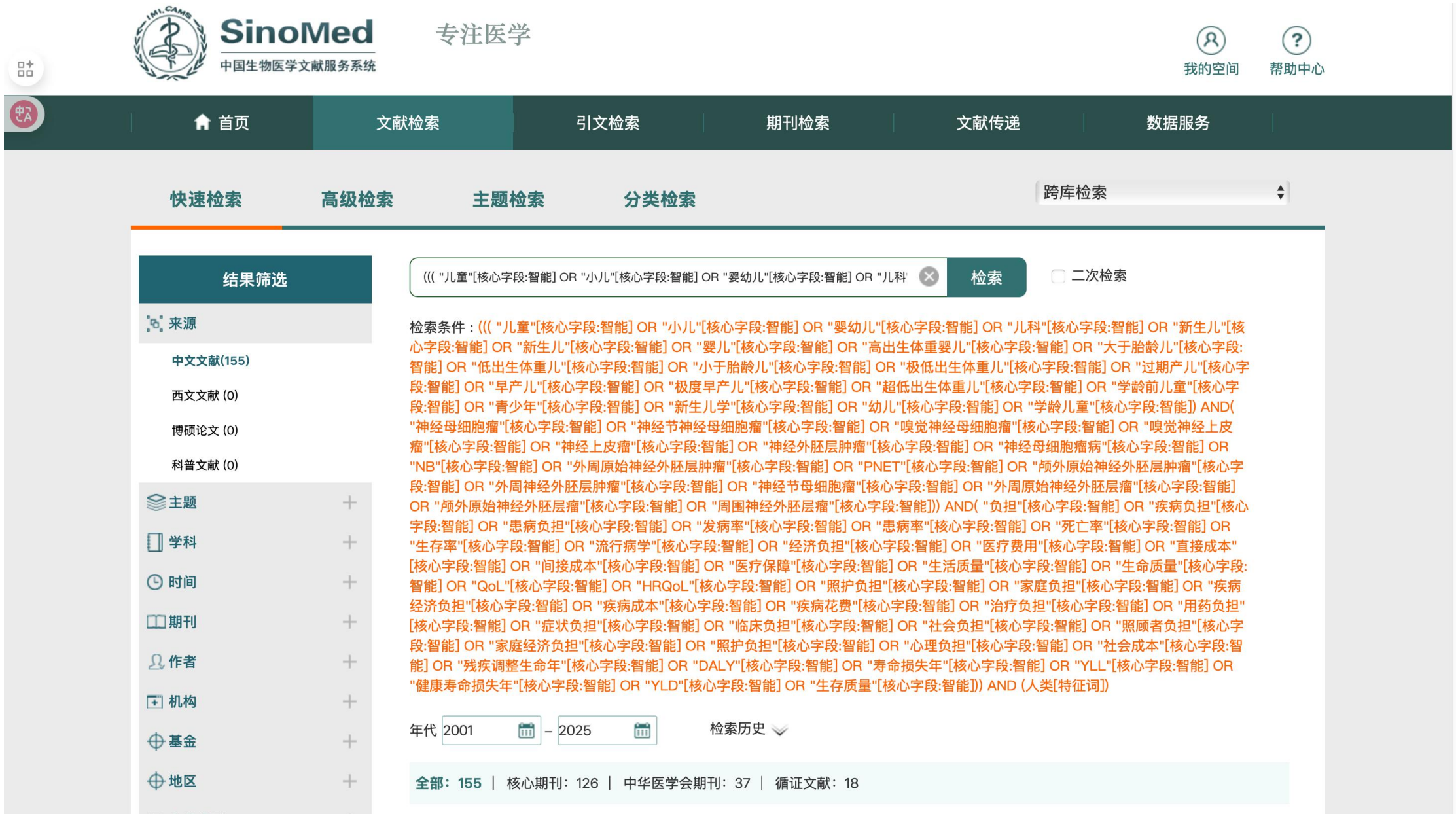

SinoMed
中国生物医学文献服务系统
专注医学
我的空间
帮助中心
首页
文献检索
引文检索
期刊检索
文献传递
数据服务
快速检索
高级检索
主题检索
分类检索
跨库检索
结果筛选
来源
中文文献(155)
西文文献 (0)
博硕论文 (0)
科普文献 (0)
主题
学科
时间
期刊
作者
机构
基金
地区
检索
二次检索
检索条件：((("儿童"[核心字段:智能] OR "小儿"[核心字段:智能] OR "婴幼儿"[核心字段:智能] OR "儿科"[核心字段:智能] OR "新生儿"[核心字段:智能] OR "新生儿"[核心字段:智能] OR "婴儿"[核心字段:智能] OR "高出生体重婴儿"[核心字段:智能] OR "大于胎龄儿"[核心字段:智能] OR "低出生体重儿"[核心字段:智能] OR "小于胎龄儿"[核心字段:智能] OR "极低出生体重儿"[核心字段:智能] OR "过期产儿"[核心字段:智能] OR "早产儿"[核心字段:智能] OR "极度早产儿"[核心字段:智能] OR "超低出生体重儿"[核心字段:智能] OR "学龄前儿童"[核心字段:智能] OR "青少年"[核心字段:智能] OR "新生儿学"[核心字段:智能] OR "幼儿"[核心字段:智能] OR "学龄儿童"[核心字段:智能]) AND("神经母细胞瘤"[核心字段:智能] OR "神经节神经母细胞瘤"[核心字段:智能] OR "嗅觉神经母细胞瘤"[核心字段:智能] OR "嗅觉神经上皮瘤"[核心字段:智能] OR "神经上皮瘤"[核心字段:智能] OR "神经外胚层肿瘤"[核心字段:智能] OR "神经母细胞瘤病"[核心字段:智能] OR "NB"[核心字段:智能] OR "外周原始神经外胚层肿瘤"[核心字段:智能] OR "PNET"[核心字段:智能] OR "颅外原始神经外胚层肿瘤"[核心字段:智能] OR "外周神经外胚层肿瘤"[核心字段:智能] OR "神经节母细胞瘤"[核心字段:智能] OR "外周原始神经外胚层瘤"[核心字段:智能] OR "颅外原始神经外胚层瘤"[核心字段:智能] OR "周围神经外胚层瘤"[核心字段:智能])) AND("负担"[核心字段:智能] OR "疾病负担"[核心字段:智能] OR "患病负担"[核心字段:智能] OR "发病率"[核心字段:智能] OR "患病率"[核心字段:智能] OR "死亡率"[核心字段:智能] OR "生存率"[核心字段:智能] OR "流行病学"[核心字段:智能] OR "经济负担"[核心字段:智能] OR "医疗费用"[核心字段:智能] OR "直接成本"[核心字段:智能] OR "间接成本"[核心字段:智能] OR "医疗保障"[核心字段:智能] OR "生活质量"[核心字段:智能] OR "生命质量"[核心字段:智能] OR "QoL"[核心字段:智能] OR "HRQoL"[核心字段:智能] OR "照护负担"[核心字段:智能] OR "家庭负担"[核心字段:智能] OR "疾病经济负担"[核心字段:智能] OR "疾病成本"[核心字段:智能] OR "疾病花费"[核心字段:智能] OR "治疗负担"[核心字段:智能] OR "用药负担"[核心字段:智能] OR "症状负担"[核心字段:智能] OR "临床负担"[核心字段:智能] OR "社会负担"[核心字段:智能] OR "照顾者负担"[核心字段:智能] OR "家庭经济负担"[核心字段:智能] OR "照护负担"[核心字段:智能] OR "心理负担"[核心字段:智能] OR "社会成本"[核心字段:智能] OR "残疾调整生命年"[核心字段:智能] OR "DALY"[核心字段:智能] OR "寿命损失年"[核心字段:智能] OR "YLL"[核心字段:智能] OR "健康寿命损失年"[核心字段:智能] OR "YLD"[核心字段:智能] OR "生存质量"[核心字段:智能])) AND (人类[特征词])
年代 2001 – 2025
检索历史
全部：155 | 核心期刊：126 | 中华医学会期刊：37 | 循证文献：18

Appendix 2
Minutes of the Structured Expert Panel Discussion

1. Meeting information

Item	Meeting record
**Meeting topic**	Development of an SDH-based multidimensional disease-burden assessment system for children with neuroblastoma
**Study stage**	Framework revision after literature extraction and before Delphi Round 1
**Date and number of meetings**	13 November 2025; one formal structured expert panel meeting. Subsequent internal synthesis by the research team was not counted as a separate meeting round.
**Meeting format**	In-person meeting
**Meeting duration**	Approximately 40 minutes
**Pre-meeting materials**	Discussion topics and a draft framework of 3 first-level, 8 second-level, and 43 third-level indicators; distributed by email in advance
**Number of participants**	10: six research-team members and four invited experts
**Mode of contribution**	Participants contributed in turn or freely around the predefined discussion guide
**Documentation**	The full meeting was audio-recorded and transcribed verbatim; two research-team members took contemporaneous notes; a postgraduate researcher cross-checked the transcript and notes and synthesized the opinions.
**Consent to participate**	Panel members indicated consent to participate by attending the meeting.
**Analytic positioning**	Consensus-oriented structured content synthesis; not a formal qualitative focus group designed for theory generation or thematic saturation

2. Participant composition

The panel comprised 10 participants: six research-team members and four invited experts.

Participant category	n	Disciplinary background	Selection/qualification
**Research-team members**	6	Pediatric nursing, public health, and postgraduate researchers	Core members of the research team
**Invited experts**	2	Diagnosis and treatment of pediatric neuroblastoma	≥10 years in the relevant field; associate-senior professional title or above
**Invited expert**	1	Pediatric neuroblastoma nursing	≥10 years in the relevant field; associate-senior professional title or above
**Invited expert**	1	Health policy	≥10 years in the relevant field; associate-senior professional title or above

3. Pre-meeting preparation and structured discussion guide

After candidate indicators had been extracted from the literature, the research team developed a pre-meeting draft comprising three first-level, eight second-level, and 43 third-level indicators. The discussion topics and draft indicator materials were distributed to participants by email in advance. The meeting was organized around the following three predefined topic groups.

Discussion topic	Core questions
**Diagnosis and treatment of pediatric NB**	Whether the preliminary physiological-health indicators captured the core health impairments; whether disease-specific functional indicators were needed; which indicators should be retained, simplified, or deleted; and how to address difficult-to-access or high-cost medicines and treatments.
**Disease burden and public health**	Healthcare accessibility, diagnostic delay, and regional resource allocation; availability of fiscal/expenditure indicators; medicines outside reimbursement lists, tiered reimbursement, targeted assistance, and cross-regional care.
**Clinical nursing**	Whether caregiving duration and caregivers' physical and psychological burden reflected practice; whether key challenges such as rehabilitation adherence and home-care safety had been omitted.

4. Discussion procedure and opinion-synthesis process

Participants contributed in turn or freely in response to the predefined questions. The entire meeting was audio-recorded and transcribed verbatim. Two research-team members also took contemporaneous notes, and a postgraduate researcher subsequently cross-checked the transcript against those notes and synthesized the opinions by predefined question and indicator item. Using the 43 pre-meeting candidate indicators as the units of analysis, the research team classified each recommendation as retain, rename, retain with a revised operational description, move and rename, split, add, or delete. The post-meeting framework was then developed with reference to the SDH framework and the five principles below. This process was intended to build consensus and preserve an audit trail of decisions; it was not designed to generate theory or achieve thematic saturation.

No.	Indicator-decision principle	Operational meaning
1	Goal orientation	Use the SDH framework to support multidimensional disease-burden assessment in children with NB.
2	Comparability and operability	Use consistent definitions wherever possible; data should be obtainable, measurable, and comparable.
3	Parsimony and scientific validity	Keep the indicator set concise and able to reflect disease burden objectively.
4	Representativeness	Prioritize typical, information-rich indicators and avoid excessive granularity.
5	Independence	Maintain clear distinctions among indicators and avoid substitution or duplication.

5. Main discussion points

No.	Topic	Main discussion points and agreed position
1	Disease heterogeneity and risk stratification	Participants noted that physical impairment, treatment pathways, and family burden differ substantially by NB risk category, disease stage, and treatment intensity; reporting only aggregate findings could obscure the burden among high-risk children. The panel recommended recording standardized stage or risk category in subsequent surveys and reporting stratified results. To avoid duplication with clinical stage, no separate indicator with an overlapping meaning such as "risk-level burden" was created.
2	Physiological health and treatment burden	The panel considered that the physiological-health dimension should prioritize patient-level indicators that are obtainable and measurable, including growth and development, the frequency and duration of healthcare visits, comorbidities, and treatment experience. "Treatment complexity" was moved to personal disease burden/physiological health burden and revised to "degree of treatment complexity." "Total treatment time" was also added to capture the time burden associated with combinations of surgery, chemotherapy, radiotherapy, and repeated healthcare visits.
3	Treatment-related adverse effects	Clinical experts considered that combining all treatment-related adverse effects into a single incidence measure would obscure differences among treatment modalities. The panel therefore split the item into "incidence of chemotherapy-related adverse reactions" and "incidence of surgery-/radiotherapy-related complications." For chemotherapy, particular attention could be given to bone-marrow suppression after multiple treatment cycles, assessed using medical records, complete blood counts, or clinical grading.
4	Diagnostic delay and healthcare accessibility	The panel recommended distinguishing the interval from symptom onset to first healthcare contact, the interval from first contact to confirmed diagnosis, and the total delay combining both intervals. Given the availability of source data, the study would prioritize the total time from first relevant symptom to confirmed diagnosis and retain the continuous duration in months, while using ≥1 month as a research threshold. This threshold was informed by Fang et al.'s single-center study of 63 Chinese children with NB, which reported a median diagnostic delay of 1.0 month (range, 0.2–24.0), with 82.5% exceeding 2 weeks and 22.2% exceeding 2 months. The panel emphasized that this was an operational threshold rather than an internationally standardized cutoff.
5	Access to medicines, treatment, and geographically distant care	Participants noted that high-cost medicines, medicines outside reimbursement lists, or treatments that were not adequately reimbursed could delay, interrupt, or alter treatment. "Access to treatment and medicines" was therefore retained as a separate indicator. The original "treatment accessibility and adherence" item was split to distinguish whether resources could be obtained from whether the child could complete the prescribed treatment. Travel distance and time were also included, while transportation and accommodation costs for cross-regional care were assigned to the family's direct nonmedical burden.
6	Family economic burden and sources of support	The panel considered that family economic burden should not be summarized by total costs alone. Family out-of-pocket medical expenditure, direct nonmedical costs, caregiver productivity loss, and catastrophic health expenditure should be recorded separately. Sources of payment or support should cover basic medical insurance, critical-illness insurance, civil-affairs assistance, commercial insurance, and other social assistance, so that insurance payments were not conflated with the family's actual burden. "Local government expenditure per capita" was deleted because disease-specific data were unavailable and could not be obtained consistently.
7	Family caregiving burden and measurement	Nursing experts noted that caregiving burden includes measurable caregiving time as well as restriction of personal time, financial stress, physical burden, effects on mental health, and distress due to insufficient disease knowledge. "Duration of informal family caregiving" was simplified to "caregiving duration." The panel recommended prioritizing validated standardized instruments for subjective burden, with on-site distribution and clarification by clinicians familiar with the child's condition to reduce missing responses and misinterpretation.
8	Quality of life, education, and social support	The panel considered that burden in children should extend beyond symptoms to psychological and emotional adjustment, social functioning, cognition and learning, family communication, and cancer-related fatigue. At the social level, cumulative school absence or dropout, family social support received, health-insurance reimbursement, and poverty status should be assessed. To avoid overlap between levels, functional impairment in the child was assigned to personal burden, effects of caregiving to family burden, and insufficient educational resources or social support to social burden.

No.	Topic	Main discussion points and agreed position
9	Applicability of composite disease-burden metrics	The panel reviewed years of life lost (YLL), years lived with disability (YLD), disability-adjusted life years (DALY), quality-adjusted life years (QALY), and years of potential life lost (YPLL). Although these metrics are valuable in public health, they require life tables, disability weights, utility values, longitudinal mortality data, or stable population denominators that could not be obtained reliably from the child and family surveys at this stage. They were therefore excluded from the Round 1 Delphi framework. This decision reflected only the operational boundary of the present stage and did not negate their potential use in future population-level burden studies.
10	Operability, independence, and framework reduction	The panel reviewed each indicator's data source, measurement definition, and overlap with adjacent indicators. Priority was given to representative, information-rich items that could be collected consistently, while items that were difficult to obtain or duplicated another construct were removed. Following discussion, the first-level structure retained the personal, family, and social domains; the number of second-level dimensions was reduced from eight to six; and the number of third-level indicators was reduced from 43 to 33. Every retention, revision, relocation, split, addition, or deletion was recorded in the change log.

6. Indicator-framework changes and audit summary

Change category	Recorded changes	Meaning
**Retained**	22	Name and position unchanged
**Retained with revised description**	1	Indicator retained and operational description refined
**Renamed**	4	Wording standardized or clarified without changing the number of indicators
**Moved and renamed**	1	Parent dimension changed and name revised
**Split**	2	Two original indicators were split into four post-meeting indicators, a net increase of two
**Deleted**	13	Thirteen indicators deleted
**Added**	1	One indicator added: "total treatment time"

6.1 Revision of the second-level structure

Of the eight pre-meeting second-level dimensions, "disease progression characteristics" and "loss of life and impairment of quality of life" were not retained as independent second-level dimensions in the post-meeting framework. The remaining six dimensions formed the framework for Round 1 of the Delphi consultation. The first-level structure remained personal disease burden, family disease burden, and social disease burden.

6.2 Deleted items (13 items)

- Clinical/course-related and population-rate indicators: physiological dysfunction, mean disease duration, survival rate, incidence, period prevalence, mortality rate, and case-fatality rate.
- Composite disease-burden indicators: years of life lost (YLL), years lived with disability (YLD), disability-adjusted life years (DALY), quality-adjusted life years (QALY), and years of potential life lost (YPLL). These indicators require external inputs such as life tables, disability weights, utility values, longitudinal mortality data, or population denominators, which were beyond the range of data that could be obtained reliably or operationalized at this stage.

- Resource-input indicator: local government expenditure per capita. The meeting record indicated that disease-specific expenditure data were unavailable and could not be obtained consistently.
- Additional note: survival rate was provisionally excluded after the panel meeting and was subsequently reintroduced as a new indicator in Delphi Round 2; content related to “physiological dysfunction” later re-entered the Delphi framework as “physical functioning.”

6.3 Splits, relocation, additions, and renaming

Split 1: “Incidence of treatment-related adverse reactions” was split into “incidence of chemotherapy-related adverse reactions” and “incidence of surgery-/radiotherapy-related complications.”

Split 2: “Treatment accessibility and adherence” was split into “access to treatment and medicines” and “treatment adherence.”

Moved and renamed: “Treatment complexity” was moved to personal disease burden/physiological health burden and was reworded as “degree of treatment complexity.”

Added: “Total treatment time” was added to capture the time burden generated by receiving multiple treatments each day or week.

Renamed 1: “Incidence of symptoms related to tumor site” was revised to “incidence of symptoms related to primary and metastatic tumor sites.”

Renamed 2: “Duration of informal family caregiving” was revised to “caregiving duration.”

Renamed 3: “Caregiver distress due to insufficient disease knowledge” was revised to “distress due to insufficient disease knowledge.”

Renamed 4: “Family social support” was revised to “family social support received.”

Retained with a revised description: “Growth and developmental status” was retained, with operational guidance expanded to include musculoskeletal adverse events.

7. Post-meeting Round 1 Delphi indicator framework (3/6/33 indicators)

First-level indicator	Second-level indicator	No. of third-level indicators	Third-level indicators
**Personal disease burden**	Physiological health burden	8	Growth and developmental status; frequency and duration of healthcare visits; degree of treatment complexity; total treatment time; incidence of symptoms related to primary and metastatic tumor sites; number of comorbidities; incidence of chemotherapy-related adverse reactions; incidence of surgery-/radiotherapy-related complications
**Personal disease burden**	Quality of life	6	Physical health and symptoms; psychological health and emotional adjustment; social functioning and interpersonal adjustment; cognitive and learning functioning; family support and communication functioning; cancer-related fatigue
**Family disease burden**	Family economic burden	4	Annual direct medical costs (family out-of-pocket medical expenditure); annual direct nonmedical costs; caregiver productivity loss; incidence of catastrophic health expenditure

First-level indicator	Second-level indicator	No. of third-level indicators	Third-level indicators
**Family disease burden**	Family caregiving burden	6	Caregiving duration; loss of personal time; financial stress; physical burden; mental health status; distress due to insufficient disease knowledge
**Social disease burden**	Accessibility of healthcare services	4	Access to treatment and medicines; travel distance and time to care; diagnostic delay; treatment adherence
**Social disease burden**	Children's education and social support	5	Cumulative duration of school absence/dropout; family social support received; health-insurance reimbursement ratio; proportion of impoverished families; expenditure in treatment and care domains

Appendix 3

Original Delphi Expert Consultation Questionnaires — Rounds 1 and 2

Part A. Round 1 questionnaire

Invitation and study context

Dear Expert,

Neuroblastoma is one of the most common extracranial solid tumors in children. Existing assessments predominantly focus on clinical outcomes and do not fully capture the multidimensional burden experienced by affected children, their families, and society. Guided by the five domains of the social determinants of health framework, the study used a literature review and structured group discussion to prepare a preliminary assessment framework.

The preliminary framework submitted for the first round comprised 3 Level 1 indicators, 6 Level 2 indicators, and 33 Level 3 indicators. Experts were invited to evaluate indicator importance and feasibility and to propose modifications, deletions, or additions. The original questionnaire requested return within one week, by 28 November 2025.

Questionnaire instructions

- The questionnaire contains two parts: consultation on the indicator system and a background survey of participating experts.
- For each applicable indicator, provide an importance score and a feasibility score from 1 to 5.
- Use the modification/deletion column to suggest wording changes or deletion. Use the prompts below each group to propose new indicators or provide other comments.
- Level 1 indicators are rated for importance only; Level 2 and Level 3 indicators are rated for both importance and feasibility.

Scoring instructions

Experts were asked to rate each indicator on two 5-point Likert scales. Select one score for each applicable dimension.

Dimension	Construct	Score	Anchors
Importance	Importance of the indicator	1–5	5 = most important; 1 = least important
Feasibility	Practicality and data accessibility/measurability	1–5	5 = most feasible; 1 = least feasible

Part I. Consultation on the preliminary indicator system

Level 1 indicators

Indicator and operational definition	Ratings	Modification / deletion
**A Individual disease burden**<br>Burden experienced by the child with neuroblastoma.	**I: ____ /5**<br>F: N/A	________________
**B Family disease burden**<br>Economic, caregiving, and related burden experienced by the family.	**I: ____ /5**<br>F: N/A	________________
**C Societal disease burden**<br>Burden related to healthcare access, education, and social support.	**I: ____ /5**<br>F: N/A	________________

Proposed additional indicator(s):

____

Other comments: ____

Level 2 indicators

Indicator and operational definition	Ratings	Modification / deletion
**A1 Physical health burden**<br>Physical health consequences associated with neuroblastoma and its treatment.	**I: ____ /5**<br>**F: ____ /5**	________________
**A2 Quality of life**<br>Functional and psychosocial effects on the child.	**I: ____ /5**<br>**F: ____ /5**	________________
**B1 Family economic burden**<br>Direct and indirect economic consequences for the family.	**I: ____ /5**<br>**F: ____ /5**	________________
**B2 Family caregiving burden**<br>Time, health, emotional, and informational burden of caregiving.	**I: ____ /5**<br>**F: ____ /5**	________________
**C1 Healthcare accessibility**<br>Ability to obtain timely diagnosis, treatment, and medicines.	**I: ____ /5**<br>**F: ____ /5**	________________
**C2 Child education and social support**<br>Educational participation and formal or informal support.	**I: ____ /5**<br>**F: ____ /5**	________________

Proposed additional indicator(s):

____

Other comments: ____

Level 3 indicators

A1. Physical health burden

Indicator and operational definition	Ratings	Modification / deletion
**A1.1 Growth and development status**<br>Height and weight; height-for-age z score (HAZ), weight-for-age z score (WAZ), and weight-for-height z score (WHZ); and musculoskeletal adverse events such as radiation-induced scoliosis.	**I: ____ /5**<br>**F: ____ /5**	________________
**A1.2 Frequency and duration of healthcare use**<br>Number of healthcare visits per year and cumulative inpatient duration per year.	**I: ____ /5**<br>**F: ____ /5**	________________
**A1.3 Treatment complexity**<br>Treatment regimens and courses, including surgery, chemotherapy alone, stem-cell transplantation plus immunotherapy, and stem-cell transplantation plus maintenance therapy.	**I: ____ /5**<br>**F: ____ /5**	________________

Indicator and operational definition	Ratings	Modification / deletion
**A1.4 Total treatment time**<br>Total time per day or week required to complete routine treatment, such as intravenous chemotherapy and radiotherapy.	**I: ____ /5**<br>**F: ____ /5**	______________
**A1.5 Incidence of symptoms related to primary and metastatic tumor sites**<br>Proportion of children with symptoms related to the primary tumor site and with symptoms related to bone or bone-marrow metastasis.	**I: ____ /5**<br>**F: ____ /5**	______________
**A1.6 Number of comorbidities**<br>Number of coexisting diseases or health conditions.	**I: ____ /5**<br>**F: ____ /5**	______________
**A1.7 Incidence of chemotherapy-related adverse events**<br>Nausea and vomiting, alopecia, myelosuppression, neurotoxicity, and other events; severity graded using the Common Terminology Criteria for Adverse Events (CTCAE).	**I: ____ /5**<br>**F: ____ /5**	______________
**A1.8 Incidence of surgery- or radiotherapy-related complications**<br>Infection, bleeding, organ impairment, skin reactions, and delayed growth or development.	**I: ____ /5**<br>**F: ____ /5**	______________

Proposed additional indicator(s):

____

Other comments: ____

A2. Quality of life

Indicator and operational definition	Ratings	Modification / deletion
**A2.1 Physical health and symptoms**<br>Physical health, disease-related symptoms, and treatment-related symptoms.	**I: ____ /5**<br>**F: ____ /5**	______________
**A2.2 Mental health and emotional adjustment**<br>Psychological well-being and emotional adaptation.	**I: ____ /5**<br>**F: ____ /5**	______________
**A2.3 Social functioning and interpersonal adjustment**<br>Social participation and interpersonal adaptation.	**I: ____ /5**<br>**F: ____ /5**	______________
**A2.4 Cognitive and learning function**<br>Cognition, learning, and school-related functioning.	**I: ____ /5**<br>**F: ____ /5**	______________
**A2.5 Family support and communication function**<br>Supportive interactions and communication within the family.	**I: ____ /5**<br>**F: ____ /5**	______________
**A2.6 Cancer-related fatigue**<br>Fatigue attributable to cancer or its treatment.	**I: ____ /5**<br>**F: ____ /5**	______________

Proposed additional indicator(s):

____

Other comments: ____

B1. Family economic burden

Indicator and operational definition	Ratings	Modification / deletion
**B1.1 Annual direct medical expenditure**<br>Annual out-of-pocket medical expenditure paid by the family.	**I: ____ /5**<br>**F: ____ /5**	______________
**B1.2 Annual direct non-medical expenditure**<br>Annual transport, accommodation, and nutritional expenditure related to seeking care.	**I: ____ /5**<br>**F: ____ /5**	______________
**B1.3 Caregiver productivity loss**<br>Work time, employment, or income lost because of caregiving.	**I: ____ /5**<br>**F: ____ /5**	______________
**B1.4 Incidence of catastrophic health expenditure**<br>Proportion of families whose out-of-pocket health expenditure divided by disposable income is at least 40%.	**I: ____ /5**<br>**F: ____ /5**	______________

Proposed additional indicator(s):

____

Other comments: ____

B2. Family caregiving burden

Indicator and operational definition	Ratings	Modification / deletion
**B2.1 Caregiving time**<br>Time spent providing care.	**I:** ____ **/5**<br>**F:** ____ **/5**	________
**B2.2 Loss of personal time**<br>Reduction in the caregiver's time for personal life and activities.	**I:** ____ **/5**<br>**F:** ____ **/5**	________
**B2.3 Financial strain**<br>Financial pressure perceived by the caregiver or family.	**I:** ____ **/5**<br>**F:** ____ **/5**	________
**B2.4 Physical burden**<br>Physical health burden associated with caregiving.	**I:** ____ **/5**<br>**F:** ____ **/5**	________
**B2.5 Mental health status**<br>Caregiver psychological and emotional health.	**I:** ____ **/5**<br>**F:** ____ **/5**	________
**B2.6 Distress due to insufficient disease knowledge**<br>Distress caused by inadequate knowledge of the disease and its care.	**I:** ____ **/5**<br>**F:** ____ **/5**	________

Proposed additional indicator(s):

____

Other comments: ____

C1. Healthcare accessibility

Indicator and operational definition	Ratings	Modification / deletion
**C1.1 Access to treatment and medicines**<br>Availability and accessibility of required treatment and medicines.	**I:** ____ **/5**<br>**F:** ____ **/5**	________
**C1.2 Travel distance and time to care**<br>Distance and time required to reach medical services.	**I:** ____ **/5**<br>**F:** ____ **/5**	________
**C1.3 Diagnostic delay**<br>Delay between symptom onset and confirmed diagnosis.	**I:** ____ **/5**<br>**F:** ____ **/5**	________
**C1.4 Treatment adherence**<br>Extent to which the prescribed treatment plan is followed.	**I:** ____ **/5**<br>**F:** ____ **/5**	________

Proposed additional indicator(s):

____

Other comments: ____

C2. Child education and social support

Indicator and operational definition	Ratings	Modification / deletion
**C2.1 Cumulative duration of school suspension or dropout**<br>Total time during which schooling or kindergarten attendance is interrupted.	**I:** ____ **/5**<br>**F:** ____ **/5**	________
**C2.2 Receipt of family social support**<br>Social support received by the child and family.	**I:** ____ **/5**<br>**F:** ____ **/5**	________
**C2.3 Health insurance reimbursement rate**<br>Proportion of eligible medical expenditure reimbursed by health insurance.	**I:** ____ **/5**<br>**F:** ____ **/5**	________
**C2.4 Proportion of households living in poverty**<br>Proportion of affected households meeting the applicable poverty criterion.	**I:** ____ **/5**<br>**F:** ____ **/5**	________
**C2.5 Treatment and care support**<br>Treatment and caregiving support provided by the state or other formal systems.	**I:** ____ **/5**<br>**F:** ____ **/5**	________

Proposed additional indicator(s):

____

Other comments: ____

Part II. Expert background questionnaire

Privacy note: The blank fields are retained to reproduce the original instrument. No completed questionnaire, expert name, affiliation, telephone number, or email address is included in this public supplementary material.

**Anonymous expert ID (public dataset)**	____________________
**Age**	____________________
**Sex**	____________________
**Professional field**	____________________
**Years of professional experience**	____________________
**Professional title**	____________________
**Highest educational qualification**	____________________
**Current position or role**	____________________
**Institution (blank field in the original instrument)**	____________________
**Contact information (blank field in the original instrument)**	____________________

Familiarity with the consultation topic

Select one: ☐ Very familiar ☐ Fairly familiar ☐ Moderately familiar ☐ Slightly familiar ☐ Unfamiliar

Basis for judgment

Basis	High	Moderate	Low
Practical experience	☐	☐	☐
Theoretical analysis	☐	☐	☐
Review of domestic and international literature	☐	☐	☐
Intuitive judgment	☐	☐	☐

Part B. Round 2 questionnaire

Invitation and study context

Dear Expert,

Thank you for completing the first-round consultation. The research team summarized the ratings and written comments, revised the indicator structure, and prepared the second-round questionnaire for renewed assessment. The original questionnaire requested return by 24 December 2025.

Questionnaire instructions

- Review the first-round score summary and the indicator revisions before rating the second-round framework.
- Provide an importance score and a feasibility score from 1 to 5 for each applicable Level 2 and Level 3 indicator.
- Level 1 indicators are rated for importance only.
- Use the modification/deletion column and the prompts below each group to provide suggestions.

Scoring instructions

Experts were asked to rate each indicator on two 5-point Likert scales. Select one score for each applicable dimension.

Dimension	Construct	Score	Anchors
Importance	Importance of the indicator	1–5	5 = most important; 1 = least important
Feasibility	Practicality and data accessibility/measurability	1–5	5 = most feasible; 1 = least feasible

First-round scores used to prepare the second-round questionnaire

Indicators were retained when the importance mean was at least 3.50 and the coefficient of variation (CV) was no greater than 0.25. A single asterisk (*) denotes an indicator modified after the first round; a double asterisk (**) denotes a newly added indicator. A slash indicates that no first-round score was available.

Indicator	Importance			Feasibility		
	Mean	CV	Full-score ratio	Mean	CV	Full-score ratio
**A. Individual disease burden**	4.75	0.09	0.75	/	/	/
**A1. Physiological health burden**	4.85	0.10	0.90	4.55	0.17	0.65
**A1.1 Growth and development status**	4.70	0.12	0.75	4.30	0.15	0.40
**A1.2 Physical functioning***	4.80	0.09	0.80	4.15	0.20	0.40
**A1.3 Tumor stage****	/	/	/	/	/	/

Indicator	Importance			Feasibility		
	Mean	CV	Full-score ratio	Mean	CV	Full-score ratio
**A1.4 Frequency and severity of pain episodes***	4.65	0.14	0.75	4.50	0.15	0.60
**A1.5 Number of comorbidities**	4.70	0.14	0.80	4.30	0.20	0.55
**A1.6 Survival****	/	/	/	/	/	/
**A2. Treatment burden****	/	/	/	/	/	/
**A2.1 Total treatment duration***	4.60	0.15	0.70	4.30	0.20	0.55
**A2.2 Treatment complexity***	4.70	0.14	0.80	4.30	0.20	0.55
**A2.3 Hospitalization frequency***	4.50	0.15	0.60	4.60	0.15	0.70
**A2.4 Incidence of chemotherapy-related adverse events**	4.80	0.11	0.85	4.55	0.13	0.60
**A2.5 Incidence of surgery- or radiotherapy-related complications**	4.60	0.15	0.70	4.40	0.19	0.55
**A3. Impact on psychosocial functioning***	4.70	0.12	0.75	4.20	0.26	0.50
**A3.1 Emotional functioning***	4.75	0.09	0.75	3.95	0.21	0.25
**A3.2 Social functioning***	4.45	0.15	0.55	3.85	0.21	0.20
**A3.3 Enrollment status (school or kindergarten)***	4.60	0.18	0.75	4.65	0.14	0.75
**A3.4 School or kindergarten performance***	4.45	0.15	0.55	3.85	0.19	0.20
**B. Family disease burden**	4.85	0.08	0.85	/	/	/
**B1. Family economic impact***	4.95	0.05	0.95	4.70	0.12	0.75
**B1.1 Family out-of-pocket medical expenditure***	4.95	0.05	0.95	4.50	0.17	0.65
**B1.2 Travel and accommodation expenses for care***	4.70	0.12	0.75	4.30	0.15	0.40
**B1.3 Caregiver productivity loss**	4.65	0.14	0.75	4.10	0.21	0.35
**B1.4 Incidence of catastrophic health expenditure**	4.65	0.14	0.75	4.20	0.23	0.50
**B2. Family caregiving burden**	4.90	0.06	0.90	4.70	0.14	0.80
**B2.1 Mean daily caregiving time***	4.90	0.06	0.90	4.50	0.15	0.60
**B2.2 Restrictions on the caregiver's personal life***	4.60	0.13	0.65	4.00	0.20	0.30
**B2.3 Health impact***	4.50	0.15	0.60	3.80	0.22	0.25
**B2.4 Emotional strain***	4.65	0.14	0.75	4.00	0.20	0.30
**B3. Social support**	4.50	0.22	0.75	4.15	0.20	0.35
**B3.1 Socioeconomic support outside basic medical insurance***	4.45	0.19	0.65	3.85	0.19	0.20
**B3.2 Emotional and psychological support****	/	/	/	/	/	/
**B3.3 Social support needs****	/	/	/	/	/	/
**C. Societal disease burden**	4.70	0.12	0.75	/	/	/
**C1. Healthcare accessibility**	4.40	0.20	0.65	4.30	0.19	0.50
**C1.1 Access to treatment and medicines**	4.70	0.14	0.80	4.05	0.20	0.35
**C1.2 Interprovincial referral***	4.20	0.20	0.45	4.25	0.20	0.50
**C1.3 Diagnostic delay***	4.50	0.20	0.70	4.10	0.19	0.35
**C2. Socioeconomic impact****	/	/	/	/	/	/
**C2.1 Mean annual total cost of diagnosis and treatment****	/	/	/	/	/	/
**C2.2 Medical insurance fund payment intensity***	4.90	0.09	0.95	4.60	0.15	0.70
**C2.3 Proportion of medical insurance expenditure on special medicines and treatments****	/	/	/	/	/	/

Part I. Second-round consultation

Level 1 indicators

Indicator and operational definition	Ratings	Modification / deletion
**A Individual disease burden**<br>Burden experienced by the child with neuroblastoma.	**I: ____ /5**<br>F: N/A	________________
**B Family disease burden**<br>Economic, caregiving, and related burden experienced by the family.	**I: ____ /5**<br>F: N/A	________________
**C Societal disease burden**<br>Burden related to healthcare access, education, and social support.	**I: ____ /5**<br>F: N/A	________________

Proposed additional indicator(s):

____

Other comments: ____

Level 2 indicators

Indicator and operational definition	Ratings	Modification / deletion
**A1 Physiological health burden**<br>Growth, physical functioning, disease severity, pain, comorbidity, and survival.	**I: ____ /5**<br>**F: ____ /5**	________________
**A2 Treatment burden**<br>Duration, complexity, hospitalization, and adverse consequences of treatment.	**I: ____ /5**<br>**F: ____ /5**	________________
**A3 Impact on psychosocial functioning**<br>Emotional, social, and educational functioning.	**I: ____ /5**<br>**F: ____ /5**	________________
**B1 Family economic impact**<br>Direct and indirect economic consequences for the family.	**I: ____ /5**<br>**F: ____ /5**	________________
**B2 Family caregiving burden**<br>Time, health, daily-life, and emotional consequences of caregiving.	**I: ____ /5**<br>**F: ____ /5**	________________
**B3 Social support**<br>Economic, emotional, psychological, and other support.	**I: ____ /5**<br>**F: ____ /5**	________________
**C1 Healthcare accessibility**<br>Access to treatment, referral, and timely diagnosis.	**I: ____ /5**<br>**F: ____ /5**	________________
**C2 Socioeconomic impact**<br>Total cost and medical insurance fund expenditure.	**I: ____ /5**<br>**F: ____ /5**	________________

Proposed additional indicator(s):

____

Other comments: ____

Level 3 indicators

A1. Physiological health burden

Indicator and operational definition	Ratings	Modification / deletion
**A1.1 Growth and development status**<br>Assess height-for-age z score (HAZ), weight-for-age z score (WAZ), and weight-for-height z score (WHZ) in accordance with the World Health Organization Child Growth Standards.	**I: ____ /5**<br>**F: ____ /5**	________________
**A1.2 Physical functioning**<br>Assess walking, exercise, self-care, and fatigue in children aged 2–18 years using the Pediatric Quality of Life Inventory (PedsQL).	**I: ____ /5**<br>**F: ____ /5**	________________

Indicator and operational definition	Ratings	Modification / deletion
**A1.3 Tumor stage**<br>Stage the tumor using the International Neuroblastoma Staging System (INSS), considering the extent of the primary tumor, midline crossing, lymph-node involvement, and distant metastasis.	I: ____ /5<br>F: ____ /5	______________
**A1.4 Frequency and severity of pain episodes**<br>Assess pain during the preceding month (for example, 0, 1–2, or 3–4 episodes per month) and severity using age-appropriate behavioral or self-report pain scales.	I: ____ /5<br>F: ____ /5	______________
**A1.5 Number of comorbidities**<br>Record the number of coexisting diseases or health conditions.	I: ____ /5<br>F: ____ /5	______________
**A1.6 Survival**<br>Assess survival and prognosis.	I: ____ /5<br>F: ____ /5	______________

Proposed additional indicator(s):

____

Other comments: ____

A2. Treatment burden

Indicator and operational definition	Ratings	Modification / deletion
**A2.1 Total treatment duration**<br>Total duration from treatment initiation to completion of the planned treatment course.	I: ____ /5<br>F: ____ /5	______________
**A2.2 Treatment complexity**<br>Complexity of the treatment regimen and the number or combination of treatment modalities.	I: ____ /5<br>F: ____ /5	______________
**A2.3 Hospitalization frequency**<br>Number or frequency of hospital admissions related to neuroblastoma treatment.	I: ____ /5<br>F: ____ /5	______________
**A2.4 Incidence of chemotherapy-related adverse events**<br>Occurrence and severity of chemotherapy-related adverse events, including nausea and vomiting, alopecia, myelosuppression, and neurotoxicity.	I: ____ /5<br>F: ____ /5	______________
**A2.5 Incidence of surgery- or radiotherapy-related complications**<br>Occurrence of complications associated with surgery or radiotherapy, including infection, bleeding, organ impairment, skin reactions, and delayed growth or development.	I: ____ /5<br>F: ____ /5	______________

Proposed additional indicator(s):

____

Other comments: ____

A3. Impact on psychosocial functioning

Indicator and operational definition	Ratings	Modification / deletion
**A3.1 Emotional functioning**<br>Assess emotional problems and adjustment associated with disease and treatment.	I: ____ /5<br>F: ____ /5	______________
**A3.2 Social functioning**<br>Assess social participation, peer interaction, and interpersonal functioning.	I: ____ /5<br>F: ____ /5	______________
**A3.3 Enrollment status (school or kindergarten)**<br>Record whether the child is enrolled in and attending school or kindergarten.	I: ____ /5<br>F: ____ /5	______________
**A3.4 School or kindergarten performance**<br>Assess attendance, learning, and performance at school or kindergarten.	I: ____ /5<br>F: ____ /5	______________

Proposed additional indicator(s):

____

Other comments: ____

B1. Family economic impact

Indicator and operational definition	Ratings	Modification / deletion
**B1.1 Family out-of-pocket medical expenditure**<br>Medical expenditure paid directly by the family.	**I: ____ /5**<br>**F: ____ /5**	____________
**B1.2 Travel and accommodation expenses for care**<br>Transport and accommodation expenditure incurred when seeking diagnosis or treatment.	**I: ____ /5**<br>**F: ____ /5**	____________
**B1.3 Caregiver productivity loss**<br>Work time, employment, or income lost because of caregiving.	**I: ____ /5**<br>**F: ____ /5**	____________
**B1.4 Incidence of catastrophic health expenditure**<br>Proportion of families whose out-of-pocket health expenditure divided by disposable income is at least 40%.	**I: ____ /5**<br>**F: ____ /5**	____________

Proposed additional indicator(s):

____

Other comments: ____

B2. Family caregiving burden

Indicator and operational definition	Ratings	Modification / deletion
**B2.1 Mean daily caregiving time**<br>Average time per day spent providing care.	**I: ____ /5**<br>**F: ____ /5**	____________
**B2.2 Restrictions on the caregiver's personal life**<br>Assess limitations on the caregiver's independent or personal life using relevant items from the Zarit Burden Interview (ZBI).	**I: ____ /5**<br>**F: ____ /5**	____________
**B2.3 Health impact**<br>Assess the impact of caregiving on the caregiver's health using relevant items from the ZBI.	**I: ____ /5**<br>**F: ____ /5**	____________
**B2.4 Emotional strain**<br>Assess emotional pressure associated with caregiving using relevant items from the ZBI.	**I: ____ /5**<br>**F: ____ /5**	____________

Proposed additional indicator(s):

____

Other comments: ____

B3. Social support

Indicator and operational definition	Ratings	Modification / deletion
**B3.1 Socioeconomic support outside basic medical insurance**<br>Economic or material support received outside the basic medical insurance system.	**I: ____ /5**<br>**F: ____ /5**	____________
**B3.2 Emotional and psychological support**<br>Emotional and psychological support available to the child and family.	**I: ____ /5**<br>**F: ____ /5**	____________
**B3.3 Social support needs**<br>Unmet or perceived needs for social support.	**I: ____ /5**<br>**F: ____ /5**	____________

Proposed additional indicator(s):

____

Other comments: ____

C1. Healthcare accessibility

Indicator and operational definition	Ratings	Modification / deletion
**C1.1 Access to treatment and medicines**<br>Availability and accessibility of required treatment and medicines.	I: ____ /5<br>F: ____ /5	______________
**C1.2 Interprovincial referral**<br>Whether diagnosis or treatment requires referral across provincial boundaries.	I: ____ /5<br>F: ____ /5	______________
**C1.3 Diagnostic delay**<br>Time from symptom onset to confirmed diagnosis; a delay of at least 1 month is considered diagnostic delay.	I: ____ /5<br>F: ____ /5	______________

Proposed additional indicator(s):

____

Other comments: ____

C2. Socioeconomic impact

Indicator and operational definition	Ratings	Modification / deletion
**C2.1 Mean annual total cost of diagnosis and treatment**<br>Mean total annual cost of diagnosis and treatment.	I: ____ /5<br>F: ____ /5	______________
**C2.2 Medical insurance fund payment intensity**<br>Medical insurance fund payment divided by total treatment cost, multiplied by 100%.	I: ____ /5<br>F: ____ /5	______________
**C2.3 Proportion of medical insurance expenditure on special medicines and treatments**<br>Reimbursed expenditure for special medicines and treatments divided by total medical insurance fund payment, multiplied by 100%; special treatment may include DFMO.	I: ____ /5<br>F: ____ /5	______________

Proposed additional indicator(s):

____

Other comments: ____

Part II. Overall expert assessment

Anonymous expert ID: ____

Overall comments on the second-round indicator system:

____

___

____

___

____

___

____

___

Appendix to Round 2: changes made after Round 1

The following revision record translates the change notes included in the original second-round questionnaire. It explains how the first-round framework was restructured before rescoring.

Level 2 indicator revisions

- A1 was renamed from Physical health burden to Physiological health burden.
- A2 Treatment burden was added.
- The former A2 Quality of life was reframed as A3 Impact on psychosocial functioning.
- B1 was renamed from Family economic burden to Family economic impact.
- B3 Social support was added.
- The former C2 Child education and social support was removed, and C2 Socioeconomic impact was added.

A1. Physiological health burden

- The operational definition of Growth and development status was revised to specify HAZ, WAZ, WHZ, and the WHO Child Growth Standards.
- Physical health and symptoms was moved from the quality-of-life domain and revised to Physical functioning.
- Tumor stage was added, with assessment using the INSS.
- Incidence of symptoms related to primary and metastatic tumor sites was revised to Frequency and severity of pain episodes.
- Survival was added.

A2. Treatment burden

- Total treatment time was moved and revised to Total treatment duration.
- Treatment complexity was moved to the new Treatment burden domain.
- Frequency and duration of healthcare use was revised to Hospitalization frequency.
- Incidence of chemotherapy-related adverse events was moved to the new Treatment burden domain.
- Incidence of surgery- or radiotherapy-related complications was moved to the new Treatment burden domain.

A3. Impact on psychosocial functioning

- Mental health and emotional adjustment was revised to Emotional functioning.
- Social functioning and interpersonal adjustment was revised to Social functioning.
- Cognitive and learning function was revised to School or kindergarten performance.
- Cumulative duration of school suspension or dropout was moved and revised to Enrollment status (school or kindergarten).

B1. Family economic impact

- Annual direct medical expenditure was revised to Family out-of-pocket medical expenditure.
- Annual direct non-medical expenditure was revised to Travel and accommodation expenses for care.

B2. Family caregiving burden

- Financial strain and Distress due to insufficient disease knowledge were removed.
- Caregiving time was revised to Mean daily caregiving time.
- Loss of personal time was revised to Restrictions on the caregiver's personal life, with reference to the ZBI.
- Physical burden was revised to Health impact, with reference to the ZBI.
- Mental health status was revised to Emotional strain, with reference to the ZBI.

B3. Social support

- Receipt of family social support was moved and revised to Socioeconomic support outside basic medical insurance.
- Emotional and psychological support was added.
- Social support needs was added.

C1. Healthcare accessibility

- Travel distance and time to care was revised to Interprovincial referral.
- The operational definition of Diagnostic delay was revised to specify the interval from symptom onset to confirmed diagnosis and a threshold of at least 1 month.
- Treatment adherence was removed.

C2. Socioeconomic impact

- Proportion of households living in poverty and Treatment and care support were removed.
- Mean annual total cost of diagnosis and treatment was added.
- Health insurance reimbursement rate was revised to Medical insurance fund payment intensity.
- Proportion of medical insurance expenditure on special medicines and treatments was added.

Appendix 4

Results of Two Rounds of Expert Consultation

and Expert Opinion Processing

Part 1. Results of Two Rounds of Expert Consultation

Results of the first round of expert inquiries via letter

			Importance Score																										
**metric**	**metric**	**Indicator Content: Expert Rating**	**1**	**2**	**3**	**4**	**5**	**6**	**7**	**8**	**9**	**10**	**11**	**12**	**13**	**14**	**15**	**16**	**17**	**18**	**19**	**20**	**average value**	**standard error**	**coefficient of variation**	**Full-score coefficient**	**maxima**	**minima**	**Full score**
		**A. Individual burden of disease**	5	5	5	5	4	5	4	5	5	5	4	5	5	5	4	4	5	5	5	5	4.75	0.44	0.09	0.75	5	4	15
		**B. The family burden of illness**	5	5	5	5	4	5	4	5	5	5	4	5	5	5	5	5	5	5	5	5	4.85	0.37	0.08	0.85	5	4	17
		**C. Social burden of disease**	5	5	3	5	4	5	4	5	5	5	4	5	5	5	5	5	4	5	5	5	4.70	0.57	0.12	0.75	5	3	15
	A. Individual burden of disease	**A1. Physical Health Burden**	5	5	5	5	5	5	5	5	5	5	4	5	5	5	5	5	5	3	5	5	4.85	0.49	0.10	0.90	5	3	18
		**A2. Quality of Life**	5	5	5	5	3	5	5	5	5	5	4	5	5	5	4	4	5	5	5	4	4.70	0.57	0.12	0.75	5	3	15
	B. The family burden of illness	**B1. Family Economic Burden**	5	5	5	5	5	5	5	5	5	5	4	5	5	5	5	5	5	5	5	5	4.95	0.22	0.05	0.95	5	4	19
		**B2. Family Care Burden**	5	5	5	5	5	5	5	5	5	5	4	5	5	5	5	5	5	5	5	4	4.90	0.31	0.06	0.90	5	4	18
	C. Social burden of disease	**C1. Accessibility of Medical Services**	5	5	3	5	3	5	3	5	5	5	3	4	5	5	3	5	5	4	5	5	4.40	0.88	0.20	0.65	5	3	13
		**C2. Children's Education and Social Support**	5	5	5	5	2	5	5	5	5	5	3	4	5	5	2	5	5	5	5	4	4.50	1.00	0.22	0.75	5	2	15
A. Individual burden of disease	A1. Physical Health Burden	**1. Growth and Development Status**	5	5	5	5	4	5	5	5	5	5	4	5	5	5	4	5	3	5	5	4	4.70	0.57	0.12	0.75	5	3	15
		**2. Frequency and Duration of Medical Visits**	5	5	3	5	4	5	4	5	5	5	4	4	5	5	4	3	5	5	5	4	4.50	0.69	0.15	0.60	5	3	12
		**3. Complexity of Treatment**	5	5	5	5	3	5	4	5	5	5	3	5	5	5	5	4	5	5	5	5	4.70	0.66	0.14	0.80	5	3	16
		**4. Total treatment duration**	5	5	3	5	3	5	4	5	5	5	4	5	5	5	5	4	4	5	5	5	4.60	0.68	0.15	0.70	5	3	14
		**5. Incidence rate of symptoms related to the primary tumor site and metastatic sites**	5	5	5	5	3	5	4	5	5	5	3	5	5	5	4	4	5	5	5	5	4.65	0.67	0.14	0.75	5	3	15
		**6. Number of comorbidities**	5	5	5	5	3	5	4	5	5	5	3	5	5	5	4	5	5	5	5	5	4.70	0.66	0.14	0.80	5	3	16
		**7. Incidence of chemotherapy-related adverse reactions**	5	5	5	5	4	5	5	5	5	5	3	5	5	5	5	5	4	5	5	5	4.80	0.52	0.11	0.85	5	3	17
		**8. Incidence of surgical/radiotherapy-related complications**	5	5	5	5	3	5	4	5	5	5	3	4	5	5	4	5	4	5	5	5	4.60	0.68	0.15	0.70	5	3	14
	A2. Quality of Life	**1. Physiological Health and Symptoms**	5	5	5	5	4	5	5	5	5	5	4	5	5	5	4	5	4	5	5	5	4.80	0.41	0.09	0.80	5	4	16

		**2. Mental Health and Emotional Adaptation**	5	5	5	5	4	4	5	5	5	5	4	5	5	5	5	5	4	5	5	4	4.75	0.44	0.09	0.75	5	4	15
		**3. Social Functions and Interpersonal Adaptation**	5	5	5	5	3	4	5	5	4	5	4	4	5	5	4	4	3	5	5	4	4.45	0.69	0.15	0.55	5	3	11
		**4. Cognitive and Learning Functions**	5	5	4	5	3	4	5	5	5	5	3	4	5	5	4	4	4	5	5	4	4.45	0.69	0.15	0.55	5	3	11
		**5. Family Support and Communication Function**	5	5	5	5	4	5	5	5	5	5	3	5	5	5	5	5	4	5	5	4	4.75	0.55	0.12	0.80	5	3	16
		**6. Cancer-related fatigue**	5	5	3	3	4	4	5	5	4	5	3	4	5	5	4	5	3	5	4	4	4.25	0.79	0.19	0.45	5	3	9
B. The family burden of illness	B1. Family Economic Burden	**1. Annual direct medical expenses (family-paid medical costs)**	5	5	5	5	4	5	5	5	5	5	5	5	5	5	5	5	5	5	5	5	4.95	0.22	0.05	0.95	5	4	19
		**2. Annual Direct Non-Medical Expenses**	5	5	5	5	3	5	4	5	5	5	5	5	5	5	4	5	4	5	5	4	4.70	0.57	0.12	0.75	5	3	15
		**3. Loss of productivity for caregivers**	5	4	5	5	3	5	5	5	5	5	5	5	5	5	3	4	5	5	5	4	4.65	0.67	0.14	0.75	5	3	15
		**4. Incidence of catastrophic medical expenditures**	5	5	5	5	3	5	4	5	4	5	5	5	5	5	3	4	5	5	5	5	4.65	0.67	0.14	0.75	5	3	15
	B2. Family Care Burden	**1. Care duration**	5	5	5	5	4	5	5	5	5	5	5	5	5	5	5	5	5	5	5	4	4.90	0.31	0.06	0.90	5	4	18
		**2. Personal time loss**	5	4	5	5	4	5	4	5	5	5	5	5	5	5	3	4	4	5	5	4	4.60	0.60	0.13	0.65	5	3	13
		**3. Economic Pressure**	5	5	5	5	4	5	5	5	5	5	4	5	5	5	3	3	5	5	5	5	4.70	0.66	0.14	0.80	5	3	16
		**4. Physical Burden**	5	5	5	5	3	5	4	5	5	5	4	4	5	5	3	4	4	5	5	4	4.50	0.69	0.15	0.60	5	3	12
		**5. Mental Health Status**	5	5	5	5	3	5	5	5	5	5	3	5	5	5	4	5	4	5	5	4	4.65	0.67	0.14	0.75	5	3	15
		**6. Insufficient disease awareness poses a challenge**	5	5	5	5	3	5	4	5	5	5	3	4	3	5	3	5	3	5	4	4	4.30	0.86	0.20	0.55	5	3	11
C. Social burden of disease	C1. Accessibility of Medical Services	**1. Treatment and Drug Accessibility**	5	5	5	5	4	5	4	5	5	5	3	5	5	5	5	3	5	5	5	5	4.70	0.66	0.14	0.80	5	3	16
		**2. Distance and Time to Medical Care**	5	5	3	5	4	5	4	5	3	5	3	4	5	5	4	3	3	5	4	4	4.20	0.83	0.20	0.45	5	3	9
		**3. Delayed Diagnosis**	5	5	5	5	4	5	5	5	5	5	3	5	5	5	3	2	4	5	5	4	4.50	0.89	0.20	0.70	5	2	14
		**4. Treatment Adherence**	5	5	5	5	4	5	5	5	5	2	3	4	3	5	4	2	4	5	4	5	4.25	1.02	0.24	0.55	5	2	11
	C2. Children's Education and Social Support	**1. Total leave/absence duration**	5	5	5	5	3	5	4	5	5	5	5	5	5	5	5	2	4	5	5	4	4.60	0.82	0.18	0.75	5	2	15
		**2. Access to Family and Social Support**	5	5	5	5	3	4	5	5	5	3	3	5	3	5	5	5	4	5	5	4	4.45	0.83	0.19	0.65	5	3	13
		**3. Medical Insurance Reimbursement Rate**	5	5	5	5	3	5	5	5	5	5	5	5	5	5	5	5	5	5	5	5	4.90	0.45	0.09	0.95	5	3	19
		**4. Proportion of impoverished households**	5	5	3	5	3	5	5	5	4	5	4	5	5	5	5	4	4	5	5	4	4.55	0.69	0.15	0.65	5	3	13
		**5. Expenditure in the areas of treatment and care**	5	5	5	5	4	5	5	5	5	3	3	5	3	5	5	4	3	5	5	5	4.50	0.83	0.18	0.70	5	3	14

**Secondary indicator:**	w=0.45	w=0.45	w=0.45
**Kendall coefficient**	2	2	2
**Third-level indicator**	w=0.12	w=0.12	w=0.12
**Kendall coefficient**	3	3	3

crest value	4.95	1.02	0.24	0.95
least value	4.20	0.22	0.05	0.45

	**Feasibility Score**																										
**Entry Content \Expert Rating**	**1**	**2**	**3**	**4**	**5**	**6**	**7**	**8**	**9**	**10**	**11**	**12**	**13**	**14**	**15**	**16**	**17**	**18**	**19**	**20**	**average value**	**standard error**	**coefficient of variation**	**Full-score coefficient**	**maxima**	**minima**	**Full score**
**A. Individual burden of disease**																											
**B. The family burden of illness**																											
**C. Social burden of disease**																											
**A1. Physical Health Burden**	5	5	4	2	4	5	5	5	5	5	4	5	5	4	5	4	5	4	5	5	4.55	0.76	0.17	0.65	5	2	13
**A2. Quality of Life**	4	4	1	2	3	5	5	5	5	5	4	4	5	4	5	4	5	5	4	5	4.20	1.11	0.26	0.50	5	1	10
**B1. Family Economic Burden**	5	4	3	5	4	5	5	5	5	5	4	5	5	5	5	4	5	5	5	5	4.70	0.57	0.12	0.75	5	3	15
**B2. Family Care Burden**	5	5	3	5	4	5	3	5	5	5	4	5	5	5	5	5	5	5	5	5	4.70	0.66	0.14	0.80	5	3	16
**C1. Accessibility of Medical Services**	4	3	4	5	3	5	3	5	5	5	4	4	5	5	3	5	4	4	5	5	4.30	0.80	0.19	0.50	5	3	10
**C2. Children's Education and Social Support**	4	3	4	4	3	5	2	5	5	5	4	4	5	4	4	5	4	5	4	4	4.15	0.81	0.20	0.35	5	2	7
**1. Growth and Development Status**	4	4	5	5	4	5	3	3	5	4	4	4	4	4	4	4	5	5	5	5	4.30	0.66	0.15	0.40	5	3	8
**2. Frequency and Duration of Medical Visits**	5	5	3	5	4	5	3	5	5	5	4	5	5	5	4	4	5	5	5	5	4.60	0.68	0.15	0.70	5	3	14
**3. Complexity of Treatment**	4	5	5	5	3	5	3	3	5	3	4	4	3	5	5	4	5	5	5	5	4.30	0.86	0.20	0.55	5	3	11
**4. Total treatment duration**	4	3	5	5	3	5	3	5	5	3	4	4	3	5	5	4	5	5	5	5	4.30	0.86	0.20	0.55	5	3	11
**5. Incidence rate of symptoms related to the primary tumor site and metastatic sites**	5	5	5	5	3	5	4	4	5	4	3	5	4	5	5	4	5	5	5	4	4.50	0.69	0.15	0.60	5	3	12
**6. Number of comorbidities**	4	5	5	5	3	5	3	4	5	3	4	4	3	5	5	5	5	5	5	3	4.30	0.86	0.20	0.55	5	3	11
**7. Incidence of chemotherapy-related adverse reactions**	5	5	5	5	4	5	3	4	5	4	4	5	4	5	4	5	5	5	5	4	4.55	0.60	0.13	0.60	5	3	12

**8. Incidence of surgical/radiotherapy-related complications**	4	5	5	5	4	5	2	4	5	4	4	4	4	5	5	5	5	5	5	3	4.40	0.82	0.19	0.55	5	2	11
**1. Physiological Health and Symptoms**	4	5	5	5	4	5	3	4	5	4	3	4	3	3	3	5	4	5	5	4	4.15	0.81	0.20	0.40	5	3	8
**2. Mental Health and Emotional Adaptation**	4	4	5	5	4	4	2	4	5	4	3	4	3	4	4	5	3	5	4	3	3.95	0.83	0.21	0.25	5	2	5
**3. Social Functions and Interpersonal Adaptation**	4	3	5	5	3	4	2	4	4	4	3	4	3	3	5	4	4	5	4	4	3.85	0.81	0.21	0.20	5	2	4
**4. Cognitive and Learning Functions**	4	3	4	5	3	4	3	4	5	5	3	4	3	4	4	3	4	5	4	3	3.85	0.75	0.19	0.20	5	3	4
**5. Family Support and Communication Function**	4	3	5	5	3	5	4	4	5	4	3	4	3	4	4	4	4	5	5	5	4.15	0.75	0.18	0.35	5	3	7
**6. Cancer-related fatigue**	4	5	3	3	3	4	2	4	4	5	3	4	5	5	5	4	3	5	4	5	4.00	0.92	0.23	0.35	5	2	7
**1. Annual direct medical expenses (family-paid medical costs)**	5	5	5	5	4	5	3	5	5	3	4	5	3	4	5	4	5	5	5	5	4.50	0.76	0.17	0.65	5	3	13
**2. Annual Direct Non-Medical Expenses**	4	4	5	5	4	5	3	5	5	4	4	5	4	4	4	4	4	5	5	3	4.30	0.66	0.15	0.40	5	3	8
**3. Loss of productivity for caregivers**	4	3	5	5	3	5	3	4	5	4	4	4	4	4	4	2	5	5	4	5	4.10	0.85	0.21	0.35	5	2	7
**4. Incidence of catastrophic medical expenditures**	4	3	5	5	3	5	3	4	2	5	4	4	5	5	4	3	5	5	5	5	4.20	0.95	0.23	0.50	5	2	10
**1. Care duration**	4	5	3	5	4	5	3	4	5	5	4	5	5	5	5	4	4	5	5	5	4.50	0.69	0.15	0.60	5	3	12
**2. Personal time loss**	4	3	5	5	3	5	3	4	5	3	4	4	3	5	3	4	4	5	4	4	4.00	0.79	0.20	0.30	5	3	6
**3. Economic Pressure**	4	4	5	5	4	5	3	4	5	3	3	4	3	5	3	3	5	5	4	4	4.05	0.83	0.20	0.35	5	3	7
**4. Physical Burden**	4	4	4	5	3	5	3	4	5	3	3	4	3	5	3	3	3	5	4	3	3.80	0.83	0.22	0.25	5	3	5
**5. Mental Health Status**	4	4	3	5	3	5	3	4	5	3	3	4	5	5	4	4	4	5	4	3	4.00	0.79	0.20	0.30	5	3	6
**6. Insufficient disease awareness poses a challenge**	4	4	3	4	3	5	4	4	5	3	3	4	3	5	3	4	3	5	4	4	3.85	0.75	0.19	0.20	5	3	4
**1. Treatment and Drug Accessibility**	4	3	5	5	4	5	3	4	5	3	3	4	3	3	5	4	4	5	4	5	4.05	0.83	0.20	0.35	5	3	7
**2. Distance and Time to Medical Care**	4	5	5	5	4	5	3	4	3	5	3	4	3	5	5	3	4	5	5	5	4.25	0.85	0.20	0.50	5	3	10
**3. Delayed Diagnosis**	4	4	5	5	4	5	3	4	5	3	3	4	3	5	4	3	4	5	5	4	4.10	0.79	0.19	0.35	5	3	7
**4. Treatment Adherence**	4	4	5	3	4	5	3	4	5	2	3	4	3	4	5	3	4	5	4	4	3.90	0.85	0.22	0.25	5	2	5
**1. Total leave/absence duration**	5	5	5	5	4	5	3	5	5	5	4	5	5	4	5	3	5	5	5	5	4.65	0.67	0.14	0.75	5	3	15
**2. Access to Family and Social Support**	4	3	3	5	4	4	3	3	5	3	3	4	3	4	5	4	4	5	4	4	3.85	0.75	0.19	0.20	5	3	4

	1	2	3	4	5	6	7	8	9	10	11	12	13	14	15	16	17	18	19	20	average value	standard error	coefficient of variation	Full-score coefficient	maxima	minima	Full score
**3. Medical Insurance Reimbursement Rate**	5	4	5	5	4	5	3	4	5	5	5	5	5	3	5	4	5	5	5	5	4.60	0.68	0.15	0.70	5	3	14
**4. Proportion of impoverished households**	4	5	3	5	4	5	3	5	4	5	3	4	5	4	5	3	5	5	5	4	4.30	0.80	0.19	0.50	5	3	10
**5. Expenditure in the areas of treatment and care**	4	4	5	5	4	5	3	4	5	3	3	4	3	5	5	4	3	5	4	4	4.10	0.79	0.19	0.35	5	3	7

crest value	4.70	1.11	0.26	0.80	
least value	3.80	0.57	0.12	0.20	

Secondary indicator: Kendall coefficient w=0.186 w=0.186 w=0.186

Third-level indicator Kendall coefficient w=0.188 w=0.188 w=0.188

Results of the second round of expert inquiries via letter

			Importance Score																											
**metric**	**metric**	**Indicator Content: Expert Rating**	**1**	**2**	**3**	**4**	**5**	**6**	**7**	**8**	**9**	**10**	**11**	**12**	**13**	**14**	**15**	**16**	**17**	**18**	**19**	**20**	**average value**	**standard error**	**coefficient of variation**	**Full-score coefficient**	**maxima**	**minima**	**Full score**	
		**A. Individual burden of disease**	5	5	5	5	5	5	5	5	5	5	5	5	4	5	4	5	5	5	5	5	4.90	0.31	0.06	0.90	5	4	18	4.9 ± 0.31
		**B. The family burden of illness**	4	5	5	5	5	5	5	5	5	5	5	5	5	5	5	5	5	5	5	5	4.95	0.22	0.05	0.95	5	4	19	4.95 ± 0.22
		**C. Social burden of disease**	4	5	4	3	5	5	4	5	5	5	5	4	3	5	3	4	4	5	5	5	4.40	0.75	0.17	0.55	5	3	11	4.4 ± 0.75
		**A1. Physiological Health Burden***	3	5	5	5	5	5	4	5	5	5	5	5	5	5	5	5	5	5	5	5	4.85	0.49	0.10	0.90	5	3	18	4.85 ± 0.49
	A. Individual burden of disease	**A2. Treatment-related burden***	4	5	5	5	5	5	4	5	5	5	5	5	5	5	5	5	5	5	5	5	4.90	0.31	0.06	0.90	5	4	18	4.9 ± 0.31
		**A3. Impact of psychosocial functions***	4	5	5	5	4	5	4	5	5	5	4	5	4	5	4	4	5	5	5	5	4.65	0.49	0.11	0.65	5	4	13	4.65 ± 0.49
		**B1. Impact on Family Economy***	5	5	5	5	4	5	4	5	5	5	5	5	4	5	5	5	5	5	5	5	4.85	0.37	0.08	0.85	5	4	17	4.85 ± 0.37
	B. The family burden of illness	**B2. Family Care Burden**	4	5	5	5	4	5	4	5	5	5	5	5	4	5	5	5	5	5	5	5	4.80	0.41	0.09	0.80	5	4	16	4.8 ± 0.41
		**B3. Social Support****	4	5	3	5	4	5	4	5	5	5	3	5	5	4	5	4	5	4	5	5	4.50	0.69	0.15	0.60	5	3	12	4.5 ± 0.69
	C. Social burden of disease	**C1. Accessibility of Medical Services**	3	5	5	5	4	5	5	5	5	5	4	4	5	5	5	4	5	4	5	5	4.65	0.59	0.13	0.70	5	3	14	4.65 ± 0.59
		**C2. Socioeconomic Impact****	4	5	4	5	3	5	4	5	5	5	4	3	3	4	4	4	5	5	4	5	4.30	0.73	0.17	0.45	5	3	9	4.3 ± 0.73
A. Individual burden	A1. Physiological Health Burden	**1.1 Growth and Development Status**	5	5	5	5	5	5	5	5	5	5	4	5	3	5	5	5	4	5	5	5	4.80	0.52	0.11	0.85	5	3	17	4.8 ± 0.52
		1.2 Physical Function*	4	5	5	5	5	5	4	5	5	5	4	5	4	5	5	5	5	5	5	5	4.80	0.41	0.09	0.80	5	4	16	4.8 ± 0.41

of disease		1.3 Tumor Stage **	5	5	5	5	5	5	4	5	5	5	4	5	4	5	5	5	5	5	5	5	4.85	0.37	0.08	0.85	5	4	17	4.85 ± 0.37
		1.4 Frequency and Severity of Pain Symptoms*	4	5	5	5	5	5	4	5	5	5	4	5	4	5	4	5	4	5	4	5	4.65	0.49	0.11	0.65	5	4	13	4.65 ± 0.49
		**1.5 Number of Comorbidities**	4	5	5	5	4	5	4	5	3	5	4	5	4	5	5	4	5	5	4	4	4.50	0.61	0.13	0.55	5	3	11	4.5 ± 0.61
		1.6 Survival Rate**	4	5	4	5	5	4	4	5	5	5	4	5	3	5	4	5	5	5	5	5	4.60	0.60	0.13	0.65	5	3	13	4.6 ± 0.6
	A2. Treatment Burden	2.1 Total Treatment Cycle*	5	5	5	5	5	4	5	5	4	5	4	5	4	5	4	4	4	5	5	5	4.65	0.49	0.11	0.65	5	4	13	4.65 ± 0.49
		2.2 Treatment Complexity*	5	5	5	5	5	5	4	5	4	5	4	5	4	5	5	5	4	5	5	5	4.75	0.44	0.09	0.75	5	4	15	4.75 ± 0.44
		2.3 Frequency of hospitalization*	4	5	4	5	4	5	3	5	5	5	4	5	4	4	5	4	5	5	5	5	4.55	0.60	0.13	0.60	5	3	12	4.55 ± 0.6
		**2.4 Incidence Rate of Chemotherapy-Related Adverse Reactions**	4	5	4	5	5	5	4	5	5	5	4	5	4	5	4	5	4	5	5	5	4.65	0.49	0.11	0.65	5	4	13	4.65 ± 0.49
		**2.5 Incidence of Surgery/Radiotherapy-Related Complications**	5	5	5	5	5	5	4	5	5	5	4	5	4	5	4	5	5	5	5	5	4.80	0.41	0.09	0.80	5	4	16	4.8 ± 0.41
	A3. Psychosocial adaptation burden	3.1 Emotional Function*	4	5	4	5	5	5	5	5	5	5	5	5	3	5	5	5	4	5	5	4	4.70	0.57	0.12	0.75	5	3	15	4.7 ± 0.57
		3.2 Social Functions*	4	5	4	4	4	5	3	5	5	5	5	5	4	5	5	5	4	5	4	5	4.55	0.60	0.13	0.60	5	3	12	4.55 ± 0.6
		3.3 Enrollment/Enrollment Status*	4	5	4	5	4	5	3	5	4	5	5	5	4	5	5	4	4	5	4	4	4.45	0.60	0.14	0.50	5	3	10	4.45 ± 0.6
		3.4 School/Kindergarten Performance*	4	5	4	5	4	5	3	5	4	5	4	5	4	5	4	4	4	5	4	5	4.40	0.60	0.14	0.45	5	3	9	4.4 ± 0.6
B. The family burden of illness	B1. Impact on Family Economy	1.1 Family-paid medical expenses*	5	5	4	5	5	5	5	5	5	5	5	5	5	5	5	5	5	5	5	5	4.95	0.22	0.05	0.95	5	4	19	4.95 ± 0.22
		1.2 Transportation/Accommodation Costs for Medical Visits*	4	5	4	5	5	5	4	5	5	5	5	5	3	5	5	4	4	4	5	4	4.55	0.60	0.13	0.60	5	3	12	4.55 ± 0.6
		**1.3 Loss of Productivity for Caregivers**	5	5	4	5	5	5	4	5	5	5	5	5	4	5	4	5	5	5	5	5	4.80	0.41	0.09	0.80	5	4	16	4.8 ± 0.41
		**1.4 Incidence of Catastrophic Medical Expenditures**	4	5	5	5	5	5	4	5	5	5	5	4	4	5	5	4	5	5	4	5	4.70	0.47	0.10	0.70	5	4	14	4.7 ± 0.47
	B2. Family Care Burden	2.1 Average daily care time*	4	5	4	5	4	5	5	5	5	5	5	4	4	4	5	5	5	5	5	4	4.65	0.49	0.11	0.65	5	4	13	4.65 ± 0.49
		2.2 Limited autonomy in daily living for caregivers*	5	5	4	5	5	5	5	5	5	5	5	5	5	5	5	5	4	5	5	4	4.85	0.37	0.08	0.85	5	4	17	4.85 ± 0.37
		2.3 Health Impacts*	5	5	4	5	5	5	5	5	5	5	5	4	4	5	4	5	4	4	5	4	4.65	0.49	0.11	0.65	5	4	13	4.65 ± 0.49
		2.4 Emotional Stress*	5	5	5	5	5	5	5	5	5	5	5	5	4	5	5	5	4	5	5	5	4.90	0.31	0.06	0.90	5	4	18	4.9 ± 0.31
	B3. Social Support and	3.1 Social and Economic Support Outside Medical Insurance*	4	5	5	5	5	5	5	5	5	5	5	5	5	4	5	4	4	4	5	5	4.75	0.44	0.09	0.75	5	4	15	4.75 ± 0.44

	Resource Access	3.2 Emotional and Psychological Support**	4	5	5	5	4	5	5	5	5	5	4	4	4	5	5	5	3	4	4	5	4.55	0.60	0.13	0.60	5	3	12	4.55 ± 0.6
		3.3 Unmet social support needs**	4	5	5	5	4	5	4	5	4	5	3	4	4	5	5	4	3	4	4	5	4.35	0.67	0.15	0.45	5	3	9	4.35 ± 0.67
C. Social burden of disease	C1. Accessibility of Medical Services	**1.1 Treatment and Drug Accessibility**	5	5	5	5	5	5	4	5	5	5	4	5	3	5	4	5	5	5	5	5	4.75	0.55	0.12	0.80	5	3	16	4.75 ± 0.55
		1.2 Inter-provincial Referral*	4	5	5	5	4	4	5	5	4	5	4	3	4	5	4	4	3	5	5	4	4.35	0.67	0.15	0.45	5	3	9	4.35 ± 0.67
		1.3 Delay in Diagnosis*	4	5	4	5	5	5	3	5	5	5	4	5	3	5	4	5	4	5	5	5	4.55	0.69	0.15	0.65	5	3	13	4.55 ± 0.69
	C2. Socioeconomic Impact	2.1 Annual Average Total Diagnosis and Treatment Cost**	4	5	5	5	5	5	3	5	5	5	5	4	4	5	5	4	4	5	5	5	4.65	0.59	0.13	0.70	5	3	14	4.65 ± 0.59
		2.2 Payment intensity of the medical insurance fund*	5	5	5	5	5	5	3	5	5	5	5	4	5	5	5	5	5	5	5	5	4.85	0.49	0.10	0.90	5	3	18	4.85 ± 0.49
		2.3 Proportion of Medical Insurance Expenditure on Special Drugs and Treatments**	5	5	5	5	5	5	3	5	4	5	5	4	4	5	5	4	4	5	5	5	4.65	0.59	0.13	0.70	5	3	14	4.65 ± 0.59

**Secondary indicator: Kendall coefficient**	**w=0.203**	**w=0.203**	**w=0.203**	crest value	4.95	0.75	0.17	0.95
**Third-level indicator Kendall coefficient**	**w=0.118**	**w=0.118**	**w=0.118**	least value	4.30	0.22	0.05	0.45

	**Feasibility Score**																											
**Entry Content \Expert Rating**	**1**	**2**	**3**	**4**	**5**	**6**	**7**	**8**	**9**	**10**	**11**	**12**	**13**	**14**	**15**	**16**	**17**	**18**	**19**	**20**	**average value**	**standard error**	**coefficient of variation**	**Full-score coefficient**	**maxima**	**minima**	**Full score**	
**A. Individual burden of disease**																												
**B. The family burden of illness**																												
**C. Social burden of disease**																												
**A1. Physiological Health Burden***	4	5	4	5	4	5	2	5	5	5	5	5	5	5	5	5	5	5	5	5	4.70	0.73	0.16	0.80	5	2	16	4.7 ± 0.73
**A2. Treatment Burden***	4	5	4	5	4	5	2	5	5	5	5	5	5	5	4	4	5	5	5	5	4.60	0.75	0.16	0.70	5	2	14	4.6 ± 0.75
**A3. Impact of psychosocial functions***	4	4	3	4	4	4	2	5	5	5	5	5	4	5	3	4	5	5	5	5	4.30	0.86	0.20	0.50	5	2	10	4.3 ± 0.86
**B1. Impact on Family**	4	5	3	5	4	5	2	5	5	5	5	5	5	5	4	5	5	5	5	5	4.60	0.82	0.18	0.75	5	2	15	4.6 ± 0.82

**Economy***																												
**B2. Family Care Burden**	4	5	4	5	4	5	5	5	5	5	5	5	4	5	5	5	5	5	5	5	4.80	0.41	0.09	0.80	5	4	16	4.8 ± 0.41
**B3. Social Support****	2	5	3	5	4	4	2	5	5	5	5	5	4	4	4	3	4	5	5	5	4.20	1.01	0.24	0.50	5	2	10	4.2 ± 1.01
**C1. Accessibility of Medical Services**	2	4	4	5	4	5	4	5	5	5	4	4	5	5	4	4	4	5	5	5	4.40	0.75	0.17	0.50	5	2	10	4.4 ± 0.75
**C2. Socioeconomic Impact****	3	4	4	5	3	5	3	5	5	5	4	4	4	4	4	3	4	5	4	5	4.15	0.75	0.18	0.35	5	3	7	4.15 ± 0.75
**1.1 Growth and Development Status**	5	5	5	5	4	5	4	5	5	5	5	4	4	5	5	5	5	5	5	5	4.80	0.41	0.09	0.80	5	4	16	4.8 ± 0.41
1.2 Physical Function*	4	5	5	5	4	4	4	4	5	5	5	5	4	5	5	4	5	5	5	4	4.60	0.50	0.11	0.60	5	4	12	4.6 ± 0.5
1.3 Tumor Stage **	4	5	5	5	4	5	4	4	5	4	5	5	4	5	4	4	5	5	5	5	4.60	0.50	0.11	0.60	5	4	12	4.6 ± 0.5
1.4 Frequency and Severity of Pain Symptoms*	2	5	5	5	4	4	3	4	5	5	5	5	4	5	3	4	5	5	4	5	4.35	0.88	0.20	0.55	5	2	11	4.35 ± 0.88
**1.5 Number of Comorbidities**	3	4	5	5	4	5	4	5	5	5	5	5	4	5	5	4	5	5	4	5	4.60	0.60	0.13	0.65	5	3	13	4.6 ± 0.6
1.6 Survival Rate**	4	5	4	5	5	4	3	5	5	3	4	5	4	5	3	4	5	5	5	5	4.40	0.75	0.17	0.55	5	3	11	4.4 ± 0.75
2.1 Total Treatment Cycle*	5	5	3	5	4	4	5	5	5	5	5	5	4	5	4	4	5	5	5	5	4.65	0.59	0.13	0.70	5	3	14	4.65 ± 0.59
2.2 Treatment Complexity*	4	5	4	5	3	5	4	5	3	4	4	5	4	5	4	4	5	5	5	5	4.40	0.68	0.15	0.50	5	3	10	4.4 ± 0.68
2.3 Frequency of hospitalization*	4	4	5	5	5	5	4	5	5	5	5	5	4	4	5	5	5	5	5	5	4.75	0.44	0.09	0.75	5	4	15	4.75 ± 0.44
**2.4 Incidence Rate of Chemotherapy-Related Adverse Reactions**	4	5	5	5	4	5	3	5	5	4	5	5	4	5	4	4	5	5	5	4	4.55	0.60	0.13	0.60	5	3	12	4.55 ± 0.6
**2.5 Incidence of Surgery/Radiotherapy-Related Complications**	5	5	5	5	4	5	5	5	5	4	5	5	4	5	4	4	5	5	5	4	4.70	0.47	0.10	0.70	5	4	14	4.7 ± 0.47
3.1 Emotional Function*	4	5	4	5	2	4	4	4	5	5	5	5	3	5	4	4	5	4	5	4	4.30	0.80	0.19	0.45	5	2	9	4.3 ± 0.8
3.2 Social Functions*	4	5	4	5	3	4	4	4	5	5	5	5	4	5	4	3	5	5	4	5	4.40	0.68	0.15	0.50	5	3	10	4.4 ± 0.68
3.3 Enrollment/Enrollment Status*	5	5	4	5	4	5	3	5	5	5	5	3	4	5	5	4	5	5	4	5	4.55	0.69	0.15	0.65	5	3	13	4.55 ± 0.69
3.4 School/Kindergarten Performance*	4	5	4	5	4	4	3	5	4	4	4	3	4	5	5	4	5	4	4	5	4.25	0.64	0.15	0.35	5	3	7	4.25 ± 0.64
1.1 Family-paid medical expenses*	5	5	4	5	5	5	4	5	5	5	5	5	4	5	5	4	5	4	5	5	4.75	0.44	0.09	0.75	5	4	15	4.75 ± 0.44
1.2 Transportation/Accommodation Costs for Medical Visits*	4	5	4	5	5	4	4	5	5	5	5	5	4	5	4	4	5	4	5	5	4.60	0.50	0.11	0.60	5	4	12	4.6 ± 0.5
**1.3 Loss of Productivity for Caregivers**	4	4	4	5	3	4	5	4	5	4	4	5	4	5	3	5	5	4	4	4	4.25	0.64	0.15	0.35	5	3	7	4.25 ± 0.64
**1.4 Incidence of Catastrophic Medical Expenditures**	4	5	5	5	3	5	4	5	4	4	4	4	4	5	4	3	5	4	4	5	4.30	0.66	0.15	0.40	5	3	8	4.3 ± 0.66

2.1 Average daily care time*	4	5	3	5	4	5	3	5	5	5	5	4	4	4	4	3	4	5	5	4	4.30	0.73	0.17	0.45	5	3	9	4.3 ± 0.73
2.2 Limited autonomy in daily living for caregivers*	4	5	3	5	4	5	3	5	5	4	4	5	4	5	3	4	5	5	5	4	4.35	0.75	0.17	0.50	5	3	10	4.35 ± 0.75
2.3 Health Impacts*	5	5	4	5	4	5	5	5	5	4	4	4	4	5	4	4	5	4	5	4	4.50	0.51	0.11	0.50	5	4	10	4.5 ± 0.51
2.4 Emotional Stress*	4	4	5	5	3	5	3	4	5	3	4	5	4	5	4	4	4	5	4	4	4.20	0.70	0.17	0.35	5	3	7	4.2 ± 0.7
3.1 Social and Economic Support Outside Medical Insurance*	5	5	5	5	4	5	3	5	5	5	5	5	4	4	5	3	5	4	5	5	4.60	0.68	0.15	0.70	5	3	14	4.6 ± 0.68
3.2 Emotional and Psychological Support**	4	3	5	5	4	5	3	4	5	3	5	4	3	5	4	3	4	4	4	4	4.05	0.76	0.19	0.30	5	3	6	4.05 ± 0.76
3.3 Needs for Social Support**	4	5	5	5	4	5	2	4	4	3	5	4	3	5	4	3	4	4	4	5	4.10	0.85	0.21	0.35	5	2	7	4.1 ± 0.85
**1.1 Treatment and Drug Accessibility**	4	5	5	5	5	5	3	5	5	4	4	4	3	5	4	3	4	4	5	5	4.35	0.75	0.17	0.50	5	3	10	4.35 ± 0.75
1.2 Inter-provincial Referral*	5	5	5	5	4	4	3	5	4	4	4	3	4	5	5	4	4	5	5	5	4.40	0.68	0.15	0.50	5	3	10	4.4 ± 0.68
1.3 Delay in Diagnosis*	5	5	4	5	5	5	3	5	4	5	4	5	3	5	5	4	4	5	5	5	4.55	0.69	0.15	0.65	5	3	13	4.55 ± 0.69
2.1 Annual Average Total Diagnosis and Treatment Cost**	4	4	5	5	5	5	3	5	5	5	5	4	4	5	5	2	5	5	5	5	4.55	0.83	0.18	0.70	5	2	14	4.55 ± 0.83
2.2 Payment intensity of the medical insurance fund*	4	5	5	5	5	5	2	5	5	5	5	4	5	5	5	3	5	4	5	5	4.60	0.82	0.18	0.75	5	2	15	4.6 ± 0.82
2.3 Proportion of Medical Insurance Expenditure on Special Drugs and Treatments**	4	5	5	5	5	5	2	5	3	5	5	4	4	5	5	2	5	4	5	5	4.40	0.99	0.23	0.65	5	2	13	4.4 ± 0.99

Secondary indicator: w=0.23 w=0.23 w=0.23
Kendall coefficient 6 6 6
Third-level indicator w=0.12 w=0.12 w=0.12
Kendall coefficient 1 1 1

crest value	4.80	1.01	0.24	0.80
least value	4.05	0.41	0.09	0.30

Basic Information Survey for Experts

	1	2	3	4	5	6	7	8	9	10	11	12	13	14	15	16	17	18	19	20
**sex**	man	woman	woman	man	man	man	woman	woman	woman	woman	woman	woman	man	woman	woman	woman	woman	woman	woman	man
**age**	58	42	49	56	40	55	64	44	52	43	58	42	42	43	44	40	35	38	57	51
**Education**	doctor	undergraduate course	undergraduate course	undergraduate course	doctor	doctor	doctor	doctor	undergraduate course	doctor	Master	Master	doctor	undergraduate course	undergraduate course	undergraduate course	Master	doctor	undergraduate course	Master

professional field	Clinical Diagnosis and Treatment	pediatric nursing	Clinical Diagnosis and Treatment	Clinical Diagnosis and Treatment	health policy	Clinical Diagnosis and Treatment	health policy	public health	health policy	public health	bedside care	pediatric nursing	public health	pediatric nursing	pediatric nursing	pediatric nursing	Clinical Diagnosis and Treatment	health policy	pediatric nursing	Clinical Diagnosis and Treatment
**professional ranks and titles**	Senior Professional Level	Senior Associate	Senior Professional Level	Senior Professional Level	Senior Professional Level	Senior Professional Level	Senior Professional Level	Senior Associate	Senior Professional Level	Senior Associate	Senior Professional Level	Senior Associate	Senior Associate	Senior Associate	Senior Associate	middle rank	middle rank	Senior Associate	Senior Professional Level	Senior Associate
**working life**	34 years	23 years	26 years	32 years	15 years	33 years	41 years	2016	29 years	15 years	35 years	20 years	10 years	23 years	22 years	20 years	11 years	7 years	38 years	24 years
**unit**	Nanjing Medical University Affiliated Children's Hospital	Nanjing Medical University Affiliated Children's Hospital	Nanjing Medical University Affiliated Children's Hospital	Nanjing Medical University Affiliated Children's Hospital	Nanjing Medical University Affiliated Children's Hospital	Jiangsu Provincial Maternal and Child Health Hospital	Nanjing Medical University Affiliated Children's Hospital	Nanjing Medical University Affiliated Children's Hospital	Nanjing Medical University Affiliated Children's Hospital	Nanjing Medical University	Jiangsu Provincial People's Hospital	Nanjing Medical University Affiliated Children's Hospital	Nanjing Medical University	Zhejiang University School of Medicine Affiliated Children's Hospital	Zhejiang University School of Medicine Affiliated Children's Hospital	Fudan University Affiliated Children's Hospital	Fudan University Affiliated Children's Hospital	Nanjing Medical University	Zhejiang University School of Medicine Affiliated Children's Hospital	Nanjing Medical University Affiliated Children's Hospital

First Round of Expert Information

	**1**	**2**	**3**	**4**	**5**	**6**	**7**	**8**	**9**	**10**	**11**	**12**	**13**	**14**	**15**	**16**	**17**	**18**	**19**	**20**
**Your level of familiarity with the investigation content**	1	1	0.8	1	0.8	0.8	0.8	0.8	0.8	0.5	0.8	1	0.5	0.8	1	0.8	0.8	0.8	0.8	1
**Expert Self-Evaluation (Based on Level)**																				
**hands-on background**	0.3	0.3	0.3	0.3	0.3	0.3	0.2	0.3	0.3	0.2	0.3	0.3	0.2	0.3	0.3	0.2	0.3	0.2	0.3	0.3
**theoretical analysis**	0.5	0.5	0.5	0.5	0.4	0.5	0.4	0.5	0.5	0.5	0.4	0.5	0.4	0.4	0.4	0.5	0.4	0.5	0.5	0.5
**Refer to domestic and international materials**	0.1	0.1	0.1	0.1	0.1	0.1	0.1	0.1	0.1	0.1	0.1	0.1	0.1	0.1	0.1	0.1	0.1	0.1	0.1	0.1
**Intuitive Choice**	0.1	0.1	0.1	0.1	0.1	0.1	0.1	0.1	0.1	0.1	0.1	0.1	0.1	0.1	0.1	0.1	0.1	0.1	0.1	0.1
**Review Suggestions/Comments**	have	have	have	have	not have	not have	not have	not have	have	have	have	have	have	not have	have	have	have	have	have	have
**Add entry**	have	have	have	have	not have	not have	not have	not have	not have	have	have	have	have	not have	not have	have	have	not have	have	have

	1	2	3	4	5	6	7	8	9	10	11	12	13	14	15	16	17	18	19	20
Your level of familiarity with the investigation content	Very familiar	Very familiar	Relatively familiar	Very familiar	Relatively familiar	Relatively familiar	Relatively familiar	Relatively familiar	Relatively familiar	Generally familiar	Relatively familiar	Very familiar	Generally familiar	Relatively familiar	Very familiar	Relatively familiar	Relatively familiar	Relatively familiar	Relatively familiar	Very familiar
Expert Self-Evaluation (Based on Level)																				
hands-on background	big	big	big	big	big	big	Middle	big	big	Middle	big	big	Middle	big	big	Middle	big	Middle	big	big
theoretical analysis	big	big	big	big	Middle	big	Middle	big	big	big	Middle	big	Middle	Middle	Middle	big	Middle	big	big	big
Refer to domestic and international materials	big	Middle	big	small	big	big	small	big	Middle	Middle	Middle	big	Middle	Middle	Middle	big	small	big	Middle	Middle
Intuitive Choice	Middle	small	big	big	Middle	Middle	Middle	Middle	big	small	small	Middle	Middle	Middle	Middle	small	Middle	small	Middle	small

first round		
Judgment Basis Ca	18.8	0.94
degree of familiarity Cs	0.83	
Expert Authority Coefficient Cr	0.885	

Results of the handling of opinions obtained from two rounds of expert inquiries via correspondence

1. Results of the first round of expert inquiry responses

The analysis results indicate that all six secondary indicators and thirty-three tertiary indicators from the first round of written consultations meet the criteria for quantitative inclusion. In accordance with the principles of indicator system development, this round of expert consultations synthesized 5 6 expert recommendations: 3 7 were fully adopted, 10 partially adoptedand 9 not adopte d.Following deliberations by the research team, the connotations and formulations ofcertain indicators were revised, resulting in the second round of expert consultati onquestionnaires comprising three primary indicators, eight secondary indicators, a ndthirty-two tertiary indicators. Specific results are presented in Tables 1 and 2.

Table 1: Results of Processing Opinions on Secondary Indicators from the First Round of Expert Consultations

content	expert opinion	handle
metric A1	1. It is recommended to amend the term to "physiological health burden".	**Original Indicator A1: Physical Health Burden**<br>Adopted and revised to "physiological health burden".
metric A2	1. It is recommended to revise this indicator to avoid overlap with the "physiological health burden" indicator, as "quality of life" is a multidimensional metric encompassing physiological function, mental health, and other aspects.<br>2. The evaluation of this indicator must account for age differences; given the young age of the children, quantifying their psychological and social functioning may pose challenges.	**Original Indicator A2: Quality of Life**<br>The original term was adopted and revised to 'psychosocial function impact.' This dimension is assessed using the Child Quality of Life Scale, which employs a 5-point Likert scale with clearly defined ratings for each level. The scale consists of a parent-rated version for children aged 2 – 8 years and a self-rated version for children aged 8 – 18 years.
metric C1	1. It is recommended to revise this indicator, as "accessibility of medical services" refers to its impact on disease management.	**Original Indicator C1: Accessibility of Medical Services**<br>Not adopted: Relevant literature uses this indicator to reflect the diagnostic and therapeutic challenges of rare tumors, as well as the implications for social resource allocation.
metric C2	1. It is recommended to revise the phrase to "social and economic impacts".<br>2. The section "Access to Family and Social Support" has been moved under the dimension "Family Burden of Disease".<br>3. It is recommended to relocate "child education" under the dimension of "individual disease burden," while	**Original Indicator C2: Children's Education and Social Support**<br>The original terms were adopted and revised as "socioeconomic impact," while "access to family and social support" was moved under the dimension of "family burden of disease." Additionally, "child education" was relocated to the dimension of "individual burden of disease."

	considering the age of affected children, as most NB patients are younger and may not yet reach school age.	
It is recommended to add indicators	1. It is recommended to add an "social support" indicator.	Adopted, adding "social support" and categorizing it under the dimension of "family burden of illness."
Other suggestions or opinions	1. Some indicators may overlap; it is recommended to reclassify primary indicators, streamline redundant or highly correlated ones, and clearly define the definitions and evaluation methods for each indicator.	Partially adopted, the classification of primary indicators was based on the definition of disease burden and the SDH theory. After discussion, the research team decided not to make any modifications and reclassified the secondary indicators to ensure their independence.
	2. Children's disease burden should be assessed by age group.	accept 。
	3. Children generally do not consider economic factors.	Not adopted. Disease burden refers to the impact of diseases on individuals, families, and society, which can affect the economic conditions of families and society.

Table 2: Results of Processing Opinions on the Third-Level Indicators from the First Round of Expert Consultations

content	expert opinion	handle
metric A1.1	1. It is recommended to specify whether this indicator is compared with healthy children or with children diagnosed with other types of tumors.	**Original indicator: Growth and development status**<br>Adopted: The indicator description was revised to "Measure height and body mass data, calculate age-specific height Z-score (HAZ), age-specific weight Z-score (WAZ), and height-specific weight Z-score (WHZ), and evaluate according to WHO standards."
metric A1.2	1. This indicator shows low correlation with individual physiological health.<br>2. Both are standard treatments, and no additional records are required for the number of medical visits. It is recommended to evaluate any additional hospitalizations, such as those caused by complications.	**Original metric: Frequency and duration of medical visits**<br>Partially adopted and moved under the "Treatment Burden" indicator, revised to "Hospitalization Frequency." The indicator description was modified to: "The frequency of hospitalizations within 1 year due to neuroblastoma and its complications in pediatric patients, stratified as 0 times, 1 − 2 times, and ⩾3 times." This indicator reflects the treatment burden on pediatric patients and can be obtained based on hospital data and family feedback, making it more reliable than data on "Additional Hospitalizations."
metric A1.3	1. It is recommended to clearly identify which treatment regimen is the most complex.	**Original metric: Complexity of treatment**<br>The adoption was approved, and the indicator description was revised to: "The types of core modalities included in the statistical treatment regimen (e.g., single modality, two-modality combination, three-modality or higher combination). Core modalities include: surgical treatment, chemotherapy, radiotherapy, targeted therapy, immunotherapy, stem cell transplantation, etc."
metric A1.4	1. It is recommended to revise the terms to "total treatment cycle" and "number of hospitalization/outpatient visits," as	**Original metric: Total treatment duration**<br>Adopted and revised to "Total Treatment Cycle," moved under the "Treatment Burden" indicator. The

	this facilitates easier data retrieval.<br>2. This indicator shows low correlation with individual physiological health.	indicator description was amended to "The total duration required from diagnosis confirmation to completion of core treatment." This indicator reflects the treatment burden on pediatric patients.
metric A1.5	1. It is recommended to emphasize the pain burden of NB patients.	**Original indicator: Incidence rate of symptoms related to the primary tumor site and metastatic sites**<br>Adopted and revised to "frequency and severity of pain symptom episodes"; the indicator description was modified to "the frequency of pain symptoms in the child over 1 month, stratified by 0 episodes/month, 1-2 episodes/month, 3-4 episodes/month, etc.; pain severity was assessed using the behavioral observation scale and self-report scale according to age groups."
metric A2.1	1. It is recommended to relocate this indicator under the "Physiological Health Burden" category.	**Original Index: Physical Health and Symptoms**<br>Adopted and moved under the "Physiological Health Burden" indicator.
metric A2.4	1. It is recommended to modify the indicators into easily assessable metrics.	**Original Index: Cognitive and Learning Functions**<br>Adopt and revise to "School/Kindergarten Performance"; revise the indicator description to "School performance of enrolled/kindergarten-enrolled children and attendance absence due to illness".
metric A2.5	1. It is recommended to delete this section, as young children have limited expressive abilities, making assessment challenging.	**Original Indicator: Family Support and Communication Function**<br>Adopt; delete this metric.
metric A2.6	1. It is recommended to delete, as the assessment of cancer-related fatigue is challenging.	**Original indicator: Cancer-related fatigue**<br>Adopt; delete this metric.
metric B1.1	1. It is recommended to amend the statement: Direct medical expenses should represent the total cost, rather than merely referring to the out-of-pocket portion borne by the family.	**Original indicator: Annual direct medical expenses (family out-of-pocket medical expenses)**<br>Adopted and revised to "Family-paid medical expenses".
metric B1.3	1. The daily average income is difficult to calculate accurately, which may lead to data discrepancies; it is recommended to introduce more flexible methods for data collection.	**Original metric: Loss of caregiver productivity**<br>Partially adopted: The average daily income can be calculated by dividing the monthly income by the number of days in a month.
metric B2.1	1. This indicator overlaps with indirect costs.<br>2. It is recommended to include time period options for caregivers to select from.	**Original metric: Duration of care**<br>Partially adopted. This indicator emphasizes the caregiving difficulty for caregivers, without considering economic factors.
metric B2.2	1. The work absenteeism rate is classified as "productivity loss for caregivers".	**Original metric: Personal time loss**<br>Adopted and revised to "limited autonomy of caregivers," with the indicator description amended to: "Use the Zarit Caregiver Burden Scale to assess whether caregivers experience restricted autonomy due to patient care, such as о т к а з ing from personal leisure or social activities, or changes in lifestyle."

metric B2.3	1. It is recommended to make modifications; allowing caregivers to define and check the options independently may lead everyone to perceive themselves as financially struggling.<br>2. It is recommended to delete this indicator, as it overlaps with the "Catastrophic Health Expenditures" indicator.	**Original Indicator: Economic Pressure**<br>Accept, delete.
metric B2.4	1. There may be difficulties in quantification.	**Original indicator: Physical burden**<br>Adopted and revised to "Health Impact"; the indicator description was amended to read: "The Zarit Caregiver Burden Scale assesses whether caregivers' health is affected by patient care."
metric B2.5	1. There may be difficulties in quantification.	**Original indicator: Mental health status**<br>Adopted and revised to "emotional stress"; the indicator description was amended to state: "The Zarit Caregiver Burden Scale assesses whether caregivers experience psychological conditions such as anxiety, depression, fatigue, helplessness, or emotional exhaustion, particularly the persistent emotional stress resulting from the long-term and complex nature of neuroblastoma care."
metric B2.6	1. Lack of objective evaluation indicators; deletion is recommended.	**Original indicator: Impaired disease awareness**<br>Accept, delete.
metric C1.2	1. The one-way travel time depends on the mode of transportation and is economically related.	**Original metric: Distance and time to healthcare facility**<br>Adopted and revised to "Inter-provincial Referral"; the indicator description was amended to state: "Whether referral across provinces is required due to inability to establish a definitive diagnosis or obtain appropriate treatment, with the primary referral reason clearly identified as diagnostic or therapeutic needs."
metric C1.3	1. Define the concept of delay clearly.<br>2. Amend to "average diagnostic delay duration".	**Original indicator: Delayed diagnosis**<br>Partially adopted; the indicator description has been revised to: "The time interval from the onset of initial symptoms to diagnosis is defined as the diagnostic time. Referencing previous literature, a diagnostic time $\geqslant$1 month is defined as diagnostic delay."
metric C1.4	1. This indicator is unreasonable; it is recommended to remove it.	**Original metric: Treatment adherence**<br>Accept, delete.
metric C2.1	1. It is recommended to conduct assessments by age group.<br>2. This indicator should not be classified under this category; instead, it should be categorized under "Personal Disease Burden".	**Original metric: Total leave duration**<br>Adopted and moved to the "Personal Disease Burden" dimension, renamed as "Enrollment/Admission Status". The indicator description was revised to: "The PedsQL scale evaluates the school performance and illness-related absenteeism of enrolled/adenored children."
metric C2.2	1. It is recommended to revise the statement to read: "Is there a need for assistance?" and "The level of assistance received."<br>2. It is recommended to move it under the dimension of "Family Burden of	**Original indicator: Access to family and social support**<br>Partially adopted and moved to the "Family Burden of Disease" dimension, revised as "Social and Economic Support Outside Medical Insurance," with the indicator description modified to "Whether the

	Disease".	individual receives other economic support beyond medical insurance funds, such as special subsidies for major illnesses, charitable assistance, social donations, crowdfunding, and financial subsidies for cross-regional medical treatment." Additionally, a new indicator titled "Social Support Needs" has been introduced.
metric C2.3	1. It is recommended to differentiate the "medical insurance reimbursement rate" into rates for conventional treatments and for expensive medications (including immunotherapies and DFMO).	**Original indicator: Medical insurance reimbursement rate**<br>The term was adopted and revised to "Medical Insurance Fund Payment Intensity." The indicator description was amended to read: "The proportion of medical insurance fund expenditures for pediatric treatment to the total treatment costs of the child, calculated as (Medical Insurance Fund Payment Amount ÷ Total Treatment Costs of the Child) × 100%." Additionally, a new indicator titled "Proportion of Medical Insurance Expenditures on Special Drugs and Treatments" was introduced.
metric C2.4	1. It is difficult to obtain objective and accurate indicators.	**Original indicator: Proportion of impoverished households**<br>Adopted; this indicator partially overlaps with household catastrophic expenditures, so it has been removed.
metric C2.5	1. Regional/area differences may exist.<br>2. The definition of this indicator is too broad and needs to be refined.<br>3. It can be combined with "social support".	**Original Indicator: Treatment and Care**<br>Adopt this approach by refining the "social support" indicator and accounting for regional differences.
It is recommended to add indicators	1. It is recommended to add "population covered by medical insurance or commercial insurance".	Not adopted; this content serves as part of the general family background survey.
	2. It is recommended to add "tumor staging".	accept 。
	3. It is recommended to add "nutritional status".	Not adopted; the "growth and development status" indicator can reflect nutritional status.
	4. It is recommended to add survival assessment indicators, such as "survival rate," which serves as a critical metric for evaluating therapeutic efficacy and the prognosis of pediatric cancer patients.	Adopted, adding "survival rate" and categorizing it under the "physiological health burden" indicator.
	5. It is recommended to add "urban/rural households" and "parents' educational attainment level".	Not adopted; this content serves as part of the general family background survey.
	6. It is recommended to add "social support".	Adopted, adding "social support" and categorizing it under the dimension of "family burden of illness."
	7. It is recommended to add "the primary family member involved in caregiving."	Not adopted; this content serves as part of the general family background survey.
	8. It is recommended to add the criterion of "number of individuals in the family requiring simultaneous care," specifically assessing whether major family members are in poor health or experience unexpected physical conditions during the child's treatment period, thereby increasing the	Not adopted; this content serves as part of the general family background survey.

	caregiving burden or financial burden.	
	9. It is recommended to enhance the assessment of the child's sleep, diet, and physical activity.	Not adopted; the "body function" indicator has already assessed this aspect.
	10. It is recommended to enhance families' willingness to provide educational support for affected children and to improve access to learning resources during the treatment period.	The finding was not adopted, as this study primarily examines the impact of NB on affected children, their families, and society; therefore, this indicator is not representative.
Other suggestions or opinions	1. Indicators such as "access to family and social support," "medical insurance reimbursement rates," and "treatment and care" all pertain to the social support dimension, exhibiting certain logical overlaps. It is recommended to clarify the core focus of each indicator.	The adopted approach involves modifying and reclassifying all three indicators to ensure their independence.

2. Results of the second round of expert inquiries and opinion processing

The analysis results indicate that all 8 secondary indicators and 32 tertiary indicators from the first round of written inquiries meet the criteria for quantitative inclusion. In accordance with the principles of indicator system construction, this round's expert inquiries synthesized 14 expert recommendations: 5 were fully adopted, 2 partially adopted, and 7 not adopted. Following deliberations by the research team, the connotations and formulations of certain indicators were revised to finalize the evaluation system, comprising 3 primary indicators, 8 secondary indicators, and 32 tertiary indicators. Specific results are presented in Tables 3 and 4.

Table 3: Results of Processing Opinions on Secondary Indicators from the Second Round of Expert Letter Consultations

content	expert opinion	handle
metric A2	1. It is recommended to amend the phrase to "treatment-related burden".	**Original metric: Treatment burden**<br>Adopted and revised to "treatment-related burden".
metric A3	1. It is recommended to revise the term to "psychosocial adaptation burden".	**Original Indicator: Impact on Psychosocial Function**<br>Adopted and revised to "psychosocial adaptation burden".
metric B3	1. It is recommended to revise the phrase to "Social Support and Resource Access".	**Original indicator: Social support**<br>Adopted: Modified to "Social Support and Resource Access".
metric C2	1. Integration with B1 is recommended.	**Original indicator: Socioeconomic impact**<br>Not adopted: The impact of medical treatment costs on families is more pronounced, while C2

reflects the societal impact of disease diagnosis and treatment costs; the separate evaluations emphasize different aspects.

Table 4: Results of Processing Opinions on Tier-3 Indicators from the Second Round of Expert Letter Consultations

content	expert opinion	handle
metric A3.4	1. It is recommended to merge with 2.3.	**Original Indicator: School/Kindergarten Performance**<br>The proposal was not adopted; indicators should be representative and serve distinct purposes. 3.3 Assess the incidence of school absenteeism due to illness, 3.4 Evaluate school performance.
metric B1.4	1. This indicator adopts the WHO definition; does it align with national conditions?	**Original indicator: Incidence of catastrophic health expenditures**<br>Not adopted; the World Health Organization (WHO), considering the national conditions of developing countries, has recommended 40% as the international reference standard.
metric B3.3	1. It is recommended to revise the phrase to "unmet social needs".<br>2. This indicator is identical to B3.1 and B3.2.	**Original indicator: Social support**<br>Partially adopted and revised to "unmet social support needs"; no duplication. B3.3 Investigates the unmet social support needs of the child's family.
metric C1.2	1. It is recommended to distinguish between proactive medical consultation and passive referral.<br>2. Add indicators such as the smoothness of transfer, timely reception, and waiting time.	**Original indicator: Inter-provincial referral**<br>Partial adoption: The indicator description is revised to state, "Whether referral across provinces occurred due to inability to establish a definitive diagnosis or obtain appropriate treatment, categorized into voluntary referral and involuntary referral; the core referral reason is clearly defined as diagnostic or therapeutic needs." Indicators should not be excessive or overly detailed; representative indicators should be selected.
metric C2.2	1. It is recommended to delete this section, as medical insurance policies vary across provinces and cities, with differing disease-specific reimbursement ratios, resulting in variations in treatment costs.	**Original indicator: Medical insurance fund payment intensity**<br>Not adopted; this indicator reflects disparities in medical insurance coverage across different regions.
It is recommended to add indicators	1. It is recommended to include "quality of life".<br>2. It is recommended to add a "graduation of chemotherapy-related toxic reactions" to reflect severity levels.<br>3. It is recommended to add "post-traumatic emergency state".	Not adopted; relevant evaluation indicators already exist. Indicators should be concise rather than complex.<br>Not adopted. Indicators should be representative; they should not be excessive or overly detailed.<br>Not adopted; this indicator does not meet the representativeness principle.

Appendix 5

Analysis Report on Weight of Evaluation System Indicators

1. Detailed weighting method and reproducibility

1.1 Source data and hierarchy

The weighting stage was performed after completion of the second Delphi round. Twenty experts rated indicator importance. The final indicator hierarchy contained three first-level indicators, eight second-level indicators, and 32 third-level indicators. Pairwise comparisons were conducted only among indicators sharing the same immediate parent.

1.2 Construction of reciprocal judgment matrices

Second-round mean importance scores were used to guide the ordering and relative magnitude of comparisons. Final judgments were expressed on Saaty's discrete 1–9 scale: 1 indicated equal importance; 3, 5, 7, and 9 indicated moderate, strong, very strong, and extreme importance; and 2, 4, 6, and 8 were intermediate judgments. Reverse comparisons used reciprocals, so $a_{ji} = 1/a_{ij}$ and $a_{ii} = 1$. The complete archived matrices, rather than rounded mean-score ratios, are the definitive computational inputs and are reproduced in Section 4.

1.3 Translation from second-round mean importance scores to Saaty scale values

The translation followed a two-step procedure: (1) scores were rank-ordered to determine the direction of pairwise dominance; (2) the magnitude of score differences guided the selection of discrete Saaty values. In practice, the study adopted a conservative scaling strategy: only the values 1, 2, 3 and their reciprocals were used. When the absolute difference $|M_i - M_j| = 0$, the scale was set to 1; for differences of 0.01–0.05, a value of 2 (or 1/2) was assigned; for differences ≥ 0.10, a value of 3 (or 1/3) was assigned. One exception occurred for B1.3 versus B1.4 ($|M_i - M_j| = 0.10$), where professional judgment overrode the score difference and a value of 1 was assigned, reflecting the researchers' assessment that caregiver productivity loss and catastrophic health expenditure are equally critical components of family economic impact. Table 1 presents the complete pairwise translation record for all 59 comparisons across 12 judgment matrices.

Table 1. Pairwise translation from mean importance scores to Saaty scale values

Matrix	Indicator i	Indicator j	Mean i	Mean j	Diff |i-j|	Ratio	Saaty a_{ij}	Direction
Matrix 2: A — Personal disease burden	A1	A2	4.85	4.90	0.05	1.0103	1/2	A1 < A2
Matrix 2: A — Personal disease burden	A1	A3	4.85	4.65	0.20	1.0430	3	A1 > A3
Matrix 2: A — Personal disease burden	A2	A3	4.90	4.65	0.25	1.0538	3	A2 > A3
Matrix 3: B — Family disease burden	B1	B2	4.85	4.80	0.05	1.0104	2	B1 > B2
Matrix 3: B — Family disease burden	B1	B3	4.85	4.50	0.35	1.0778	3	B1 > B3
Matrix 3: B — Family disease burden	B2	B3	4.80	4.50	0.30	1.0667	3	B2 > B3
Matrix 4: C — Social disease burden	C1	C2	4.65	4.30	0.35	1.0814	3	C1 > C2
Matrix 5: A1 — Physiological health burden	A1.1	A1.2	4.80	4.80	0.00	1.0000	1	A1.1 = A1.2
Matrix 5: A1 — Physiological health burden	A1.1	A1.3	4.80	4.85	0.05	1.0104	1/2	A1.1 < A1.3
Matrix 5: A1 — Physiological health burden	A1.1	A1.4	4.80	4.65	0.15	1.0323	3	A1.1 > A1.4

Matrix	Indicator i	Indicator j	Mean i	Mean j	Diff |i-j|	Ratio	Saaty a_{ij}	Direction
Matrix 5: A1 — Physiological health burden	A1.1	A1.5	4.80	4.50	0.30	1.0667	3	A1.1 > A1.5
Matrix 5: A1 — Physiological health burden	A1.1	A1.6	4.80	4.60	0.20	1.0435	3	A1.1 > A1.6
Matrix 5: A1 — Physiological health burden	A1.2	A1.3	4.80	4.85	0.05	1.0104	1/2	A1.2 < A1.3
Matrix 5: A1 — Physiological health burden	A1.2	A1.4	4.80	4.65	0.15	1.0323	3	A1.2 > A1.4
Matrix 5: A1 — Physiological health burden	A1.2	A1.5	4.80	4.50	0.30	1.0667	3	A1.2 > A1.5
Matrix 5: A1 — Physiological health burden	A1.2	A1.6	4.80	4.60	0.20	1.0435	3	A1.2 > A1.6
Matrix 5: A1 — Physiological health burden	A1.3	A1.4	4.85	4.65	0.20	1.0430	3	A1.3 > A1.4
Matrix 5: A1 — Physiological health burden	A1.3	A1.5	4.85	4.50	0.35	1.0778	3	A1.3 > A1.5
Matrix 5: A1 — Physiological health burden	A1.3	A1.6	4.85	4.60	0.25	1.0543	3	A1.3 > A1.6
Matrix 5: A1 — Physiological health burden	A1.4	A1.5	4.65	4.50	0.15	1.0333	3	A1.4 > A1.5
Matrix 5: A1 — Physiological health burden	A1.4	A1.6	4.65	4.60	0.05	1.0109	2	A1.4 > A1.6
Matrix 5: A1 — Physiological health burden	A1.5	A1.6	4.50	4.60	0.10	1.0222	1/2	A1.5 < A1.6
Matrix 6: A2 — Treatment-related burden	A2.1	A2.2	4.65	4.75	0.10	1.0215	1/3	A2.1 < A2.2
Matrix 6: A2 — Treatment-related burden	A2.1	A2.3	4.65	4.55	0.10	1.0220	3	A2.1 > A2.3
Matrix 6: A2 — Treatment-related burden	A2.1	A2.4	4.65	4.65	0.00	1.0000	1	A2.1 = A2.4
Matrix 6: A2 — Treatment-related burden	A2.1	A2.5	4.65	4.80	0.15	1.0323	1/3	A2.1 < A2.5
Matrix 6: A2 — Treatment-related burden	A2.2	A2.3	4.75	4.55	0.20	1.0440	3	A2.2 > A2.3
Matrix 6: A2 — Treatment-related burden	A2.2	A2.4	4.75	4.65	0.10	1.0215	3	A2.2 > A2.4
Matrix 6: A2 — Treatment-related burden	A2.2	A2.5	4.75	4.80	0.05	1.0105	1/2	A2.2 < A2.5
Matrix 6: A2 — Treatment-related burden	A2.3	A2.4	4.55	4.65	0.10	1.0220	1/3	A2.3 < A2.4
Matrix 6: A2 — Treatment-related burden	A2.3	A2.5	4.55	4.80	0.25	1.0549	1/3	A2.3 < A2.5
Matrix 6: A2 — Treatment-related burden	A2.4	A2.5	4.65	4.80	0.15	1.0323	1/3	A2.4 < A2.5
Matrix 7: A3 — Psychosocial adaptation burden	A3.1	A3.2	4.70	4.55	0.15	1.0330	3	A3.1 > A3.2
Matrix 7: A3 — Psychosocial adaptation burden	A3.1	A3.3	4.70	4.45	0.25	1.0562	3	A3.1 > A3.3
Matrix 7: A3 — Psychosocial adaptation burden	A3.1	A3.4	4.70	4.40	0.30	1.0682	3	A3.1 > A3.4
Matrix 7: A3 — Psychosocial adaptation burden	A3.2	A3.3	4.55	4.45	0.10	1.0225	3	A3.2 > A3.3
Matrix 7: A3 — Psychosocial adaptation burden	A3.2	A3.4	4.55	4.40	0.15	1.0341	3	A3.2 > A3.4
Matrix 7: A3 — Psychosocial adaptation burden	A3.3	A3.4	4.45	4.40	0.05	1.0114	2	A3.3 > A3.4
Matrix 8: B1 — Family economic impact	B1.1	B1.2	4.95	4.55	0.40	1.0879	3	B1.1 > B1.2
Matrix 8: B1 — Family economic impact	B1.1	B1.3	4.95	4.80	0.15	1.0312	3	B1.1 > B1.3
Matrix 8: B1 — Family economic impact	B1.1	B1.4	4.95	4.70	0.25	1.0532	3	B1.1 > B1.4
Matrix 8: B1 — Family economic impact	B1.2	B1.3	4.55	4.80	0.25	1.0549	1/3	B1.2 < B1.3
Matrix 8: B1 — Family economic impact	B1.2	B1.4	4.55	4.70	0.15	1.0330	1/3	B1.2 < B1.4
Matrix 8: B1 — Family economic impact	B1.3	B1.4	4.80	4.70	0.10	1.0213	1	B1.3 > B1.4
Matrix 9: B2 — Family caregiving burden	B2.1	B2.2	4.65	4.85	0.20	1.0430	1/3	B2.1 < B2.2
Matrix 9: B2 — Family caregiving burden	B2.1	B2.3	4.65	4.65	0.00	1.0000	1	B2.1 = B2.3
Matrix 9: B2 — Family caregiving burden	B2.1	B2.4	4.65	4.90	0.25	1.0538	1/3	B2.1 < B2.4
Matrix 9: B2 — Family caregiving burden	B2.2	B2.3	4.85	4.65	0.20	1.0430	3	B2.2 > B2.3

Matrix	Indicator i	Indicator j	Mean i	Mean j	Diff |i-j|	Ratio	Saaty a_{ij}	Direction
Matrix 9: B2 — Family caregiving burden	B2.2	B2.4	4.85	4.90	0.05	1.0103	1/2	B2.2 < B2.4
Matrix 9: B2 — Family caregiving burden	B2.3	B2.4	4.65	4.90	0.25	1.0538	1/3	B2.3 < B2.4
Matrix 10: B3 — Social support and resource access	B3.1	B3.2	4.75	4.55	0.20	1.0440	3	B3.1 > B3.2
Matrix 10: B3 — Social support and resource access	B3.1	B3.3	4.75	4.35	0.40	1.0920	3	B3.1 > B3.3
Matrix 10: B3 — Social support and resource access	B3.2	B3.3	4.55	4.35	0.20	1.0460	2	B3.2 > B3.3
Matrix 11: C1 — Accessibility of healthcare services	C1.1	C1.2	4.75	4.35	0.40	1.0920	3	C1.1 > C1.2
Matrix 11: C1 — Accessibility of healthcare services	C1.1	C1.3	4.75	4.55	0.20	1.0440	2	C1.1 > C1.3
Matrix 11: C1 — Accessibility of healthcare services	C1.2	C1.3	4.35	4.55	0.20	1.0460	1/3	C1.2 < C1.3
Matrix 12: C2 — Socioeconomic impact	C2.1	C2.2	4.65	4.85	0.20	1.0430	1/3	C2.1 < C2.2
Matrix 12: C2 — Socioeconomic impact	C2.1	C2.3	4.65	4.65	0.00	1.0000	1	C2.1 = C2.3
Matrix 12: C2 — Socioeconomic impact	C2.2	C2.3	4.85	4.65	0.20	1.0430	3	C2.2 > C2.3

1.4 Local weights

For each positive reciprocal matrix **A**, the local priority vector **w** was obtained from the principal right eigenvector: $\mathbf{A}\,\mathbf{w} = \lambda_{max}\,\mathbf{w}$. The vector was normalized so that the local weights within each sibling set summed to 1.

1.5 Consistency testing

For a matrix of order n, the consistency index was $CI = (\lambda_{max} - n)/(n - 1)$, and the consistency ratio was CR = CI/RI, where RI is the random-index constant used by Yaahp for the same matrix order. A matrix was accepted when CR < 0.10. Two-item matrices are perfectly reciprocal by construction and were reported as CR = 0.0000. The RI constants below reproduce the archived software output.

Matrix order (<i>n</i>)	2	3	4	5	6
Random index (RI)	0.00	0.52	0.89	1.12	1.26

2. Ranked third-level indicator weights

The ranked presentation below responds directly to the need to identify which components of the assessment system received the greatest priority. Global weights sum to 1.0000 across all 32 third-level indicators.

Rank	Code	Third-level indicator	Parent	Global	Local
1	B1.1	Family out-of-pocket medical expenditure	B1	0.1358	0.4874
2	B2.4	Emotional stress	B2	0.0778	0.4435
3	A2.5	Incidence of surgery- or radiotherapy-related complications	A2	0.0657	0.3742
4	B1.3	Caregiver productivity loss	B1	0.0581	0.2085
5	B1.4	Incidence of catastrophic health expenditure	B1	0.0581	0.2085
6	C1.1	Access to treatment and medications	C1	0.0553	0.5278
7	B2.2	Restriction of caregivers' independent life	B2	0.0548	0.3121
8	A2.2	Treatment complexity	A2	0.0499	0.2845
9	B3.1	Socioeconomic support beyond medical insurance	B3	0.0438	0.5936
10	C1.3	Diagnostic delay	C1	0.0348	0.3325
11	A1.3	Tumor staging	A1	0.0343	0.3104
12	B1.2	Transportation and accommodation costs for medical visits	B1	0.0266	0.0956
13	A1.1	Growth and developmental status	A1	0.0239	0.2166
14	A1.2	Physical functioning	A1	0.0239	0.2166
15	A2.1	Total treatment duration	A2	0.0236	0.1343
16	A2.4	Incidence of chemotherapy-related adverse reactions	A2	0.0236	0.1343
17	A3.1	Emotional functioning	A3	0.0224	0.4827
18	B2.1	Average daily caregiving time	B2	0.0215	0.1222
19	B2.3	Health impact on caregivers	B2	0.0215	0.1222
20	C2.2	Intensity of medical insurance fund payment	C2	0.0209	0.6000
21	B3.2	Emotional and psychological support	B3	0.0184	0.2493
22	C1.2	Cross-provincial referral	C1	0.0146	0.1396
23	A3.2	Social functioning	A3	0.0128	0.2756
24	A2.3	Hospitalization frequency	A2	0.0127	0.0726
25	A1.4	Frequency and severity of pain episodes	A1	0.0125	0.1132

Rank	Code	Third-level indicator	Parent	Global	Local
26	B3.3	Unmet social support needs	B3	0.0116	0.1571
27	A1.6	Survival status	A1	0.0090	0.0813
28	C2.1	Average annual total diagnostic and treatment cost	C2	0.0070	0.2000
29	C2.3	Proportion of insurance expenditure for special drugs and treatments	C2	0.0070	0.2000
30	A1.5	Number of comorbidities	A1	0.0068	0.0619
31	A3.3	Kindergarten or school enrollment status	A3	0.0066	0.1412
32	A3.4	Kindergarten or school performance	A3	0.0047	0.1006
		**Total global weight**		**1.0000**	

3. Consistency audit across all judgment matrices

All 12 matrices passed the prespecified CR < 0.10 criterion. The highest CR was 0.0806 for psychosocial adaptation burden. This result supports internal coherence of the comparisons but should not be interpreted as external validation of the indicator system.

Matrix	Parent	<i>n</i>	λ_{max}	CR	Decision
1	Goal: Overall assessment system	3	3.0536	0.0516	Accept
2	A: Personal disease burden	3	3.0536	0.0516	Accept
3	B: Family disease burden	3	3.0536	0.0516	Accept
4	C: Social disease burden	2	2.0000	0.0000	Accept
5	A1: Physiological health burden	6	6.2683	0.0426	Accept
6	A2: Treatment-related burden	5	5.2565	0.0573	Accept
7	A3: Psychosocial adaptation burden	4	4.2153	0.0806	Accept
8	B1: Family economic impact	4	4.1545	0.0579	Accept
9	B2: Family caregiving burden	4	4.0606	0.0227	Accept
10	B3: Social support and resource access	3	3.0536	0.0516	Accept
11	C1: Accessibility of healthcare services	3	3.0536	0.0516	Accept
12	C2: Socioeconomic impact	3	3.0000	0.0000	Accept
	**Combined CR for second-level indicators**			**0.0484****Accept**	

4. Complete archived pairwise judgment matrices

The following tables provide the full reciprocal inputs required to reproduce every local weight and consistency result. Fractions are retained exactly as recorded in the archived calculation report.

Matrix 1. Goal: Overall assessment system
Parent global weight = 1.0000; n = 3; λ_{max} = 3.0536; CR = 0.0516 (acceptable).
A = Personal disease burden; B = Family disease burden; C = Social disease burden

Item	A	B	C	Local weight
A	1	1/2	3	0.3325
B	2	1	3	0.5278
C	1/3	1/3	1	0.1396

Matrix 2. A: Personal disease burden
Parent global weight = 0.3325; n = 3; λ_{max} = 3.0536; CR = 0.0516 (acceptable).
A1 = Physiological health burden; A2 = Treatment-related burden; A3 = Psychosocial adaptation burden

Item	A1	A2	A3	Local weight
A1	1	1/2	3	0.3325
A2	2	1	3	0.5278
A3	1/3	1/3	1	0.1396

Matrix 3. B: Family disease burden

Parent global weight = 0.5278; n = 3; λ_{max} = 3.0536; CR = 0.0516 (acceptable).

B1 = Family economic impact; B2 = Family caregiving burden; B3 = Social support and resource access

Item	B1	B2	B3	Local weight
B1	1	2	3	0.5278
B2	1/2	1	3	0.3325
B3	1/3	1/3	1	0.1396

Matrix 4. C: Social disease burden

Parent global weight = 0.1396; n = 2; λ_{max} = 2.0000; CR = 0.0000 (acceptable).

C1 = Accessibility of healthcare services; C2 = Socioeconomic impact

Item	C1	C2	Local weight
C1	1	3	0.7500
C2	1/3	1	0.2500

Matrix 5. A1: Physiological health burden

Parent global weight = 0.1106; *n* = 6; λ_{max} = 6.2683; CR = 0.0426 (acceptable).

A1.1 = Growth and developmental status; A1.2 = Physical functioning; A1.3 = Tumor staging; A1.4 = Frequency and severity of pain episodes; A1.5 = Number of comorbidities; A1.6 = Survival status

Item	A1.1	A1.2	A1.3	A1.4	A1.5	A1.6	Local weight
A1.1	1	1	1/2	3	3	3	0.2166
A1.2	1	1	1/2	3	3	3	0.2166
A1.3	2	2	1	3	3	3	0.3104
A1.4	1/3	1/3	1/3	1	3	2	0.1132
A1.5	1/3	1/3	1/3	1/3	1	1/2	0.0619
A1.6	1/3	1/3	1/3	1/2	2	1	0.0813

Matrix 6. A2: Treatment-related burden

Parent global weight = 0.1755; *n* = 5; λ_{max} = 5.2565; CR = 0.0573 (acceptable).

A2.1 = Total treatment duration; A2.2 = Treatment complexity; A2.3 = Hospitalization frequency; A2.4 = Incidence of chemotherapy-related adverse reactions; A2.5 = Incidence of surgery- or radiotherapy-related complications

Item	A2.1	A2.2	A2.3	A2.4	A2.5	Local weight
A2.1	1	1/3	3	1	1/3	0.1343
A2.2	3	1	3	3	1/2	0.2845
A2.3	1/3	1/3	1	1/3	1/3	0.0726
A2.4	1	1/3	3	1	1/3	0.1343
A2.5	3	2	3	3	1	0.3742

Matrix 7. A3: Psychosocial adaptation burden

Parent global weight = 0.0464; *n* = 4; λ_{max} = 4.2153; CR = 0.0806 (acceptable).

A3.1 = Emotional functioning; A3.2 = Social functioning; A3.3 = Kindergarten or school enrollment status; A3.4 = Kindergarten or school performance

Item	A3.1	A3.2	A3.3	A3.4	Local weight
A3.1	1	3	3	3	0.4827
A3.2	1/3	1	3	3	0.2756
A3.3	1/3	1/3	1	2	0.1412
A3.4	1/3	1/3	1/2	1	0.1006

Matrix 8. B1: Family economic impact

Parent global weight = 0.2786; *n* = 4; λ_{max} = 4.1545; CR = 0.0579 (acceptable).

B1.1 = Family out-of-pocket medical expenditure; B1.2 = Transportation and accommodation costs for medical visits; B1.3 = Caregiver productivity loss; B1.4 = Incidence of catastrophic health expenditure

Item	B1.1	B1.2	B1.3	B1.4	Local weight
B1.1	1	3	3	3	0.4874
B1.2	1/3	1	1/3	1/3	0.0956
B1.3	1/3	3	1	1	0.2085

Item	B1.1	B1.2	B1.3	B1.4	Local weight
B1.4	1/3	3	1	1	0.2085

Matrix 9. B2: Family caregiving burden

Parent global weight = 0.1755; *n* = 4; λ_{max} = 4.0606; CR = 0.0227 (acceptable).

B2.1 = Average daily caregiving time; B2.2 = Restriction of caregivers' independent life; B2.3 = Health impact on caregivers; B2.4 = Emotional stress

Item	B2.1	B2.2	B2.3	B2.4	Local weight
B2.1	1	1/3	1	1/3	0.1222
B2.2	3	1	3	1/2	0.3121
B2.3	1	1/3	1	1/3	0.1222
B2.4	3	2	3	1	0.4435

Matrix 10. B3: Social support and resource access

Parent global weight = 0.0737; *n* = 3; λ_{max} = 3.0536; CR = 0.0516 (acceptable).

B3.1 = Socioeconomic support beyond medical insurance; B3.2 = Emotional and psychological support; B3.3 = Unmet social support needs

Item	B3.1	B3.2	B3.3	Local weight
B3.1	1	3	3	0.5936
B3.2	1/3	1	2	0.2493
B3.3	1/3	1/2	1	0.1571

Matrix 11. C1: Accessibility of healthcare services

Parent global weight = 0.1047; *n* = 3; λ_{max} = 3.0536; CR = 0.0516 (acceptable).

C1.1 = Access to treatment and medications; C1.2 = Cross-provincial referral; C1.3 = Diagnostic delay

Item	C1.1	C1.2	C1.3	Local weight
C1.1	1	3	2	0.5278
C1.2	1/3	1	1/3	0.1396
C1.3	1/2	3	1	0.3325

Matrix 12. C2: Socioeconomic impact

Parent global weight = 0.0349; *n* = 3; λ_{max} = 3.0000; CR = 0.0000 (acceptable).

C2.1 = Average annual total diagnostic and treatment cost; C2.2 = Intensity of medical insurance fund payment; C2.3 = Proportion of insurance expenditure for special drugs and treatments

Item	C2.1	C2.2	C2.3	Local weight
C2.1	1	1/3	1	0.2000
C2.2	3	1	3	0.6000
C2.3	1	1/3	1	0.2000
